# Discovery of a PCAF Bromodomain Chemical Probe

**DOI:** 10.1002/anie.201610816

**Published:** 2016-12-14

**Authors:** Moses Moustakim, Peter G. K. Clark, Laura Trulli, Angel L. Fuentes de Arriba, Matthias T. Ehebauer, Apirat Chaikuad, Emma J. Murphy, Jacqui Mendez‐Johnson, Danette Daniels, Chun‐Feng D. Hou, Yu‐Hui Lin, John R. Walker, Raymond Hui, Hongbing Yang, Lucy Dorrell, Catherine M. Rogers, Octovia P. Monteiro, Oleg Fedorov, Kilian V. M. Huber, Stefan Knapp, Jag Heer, Darren J. Dixon, Paul E. Brennan

**Affiliations:** ^1^ Structural Genomics Consortium & Target Discovery InstituteUniversity of OxfordNDM Research BuildingRoosevelt DriveOxfordOX3 7DQ and OX3 7FZUK; ^2^ Department of ChemistryChemistry Research LaboratoryUniversity of OxfordMansfield RoadOxfordOX1 3TAUK; ^3^ Department of ChemistrySimon Fraser UniversityBurnabyV5A 1S6Canada; ^4^ Dipartimento di ChimicaUniversità degli Studi di Roma “La Sapienza”Piazzale Aldo Moro 500185RomaItaly; ^5^ ARUK Oxford Drug Discovery InstituteUniversity of OxfordOxfordOX3 7FZUK; ^6^ Johann Wolfgang Goethe-UniversityInstitute for Pharmaceutical Chemistry and Buchmann Institute for Life Sciences60438Frankfurt am MainGermany; ^7^ Promega Corporation2800 Woods Hollow RoadMadisonWI153611USA; ^8^ Structural Genomics ConsortiumMaRS South Tower, Suite 732101 College StreetTorontoOntarioM5G 1LZCanada; ^9^ Nuffield Department of Medicine and Oxford NIHR Biomedical Research CentreUniversity of OxfordOxfordOX3 7FZUK; ^10^ UCB Pharma LtdSloughSL1 3WEUK

**Keywords:** bromodomains, chemical probes, epigenetics, medicinal chemistry, structure-based design

## Abstract

The p300/CBP‐associated factor (PCAF) and related GCN5 bromodomain‐containing lysine acetyl transferases are members of subfamily I of the bromodomain phylogenetic tree. Iterative cycles of rational inhibitor design and biophysical characterization led to the discovery of the triazolopthalazine‐based **L‐45** (dubbed **L‐Moses**) as the first potent, selective, and cell‐active PCAF bromodomain (Brd) inhibitor. Synthesis from readily available (1R,2S)‐(−)‐norephedrine furnished **L‐45** in enantiopure form. **L‐45** was shown to disrupt PCAF‐Brd histone H3.3 interaction in cells using a nanoBRET assay, and a co‐crystal structure of **L‐45** with the homologous Brd PfGCN5 from Plasmodium falciparum rationalizes the high selectivity for PCAF and GCN5 bromodomains. Compound **L‐45** shows no observable cytotoxicity in peripheral blood mononuclear cells (PBMC), good cell‐permeability, and metabolic stability in human and mouse liver microsomes, supporting its potential for in vivo use.

Bromodomains proteins (Brds) bind to acetylated lysines (KAc) through the Brd acetyllysine‐binding site. Misregulation of these proteins is linked to the onset and progression of multiple disease states, such as cancer.^1^ Significant efforts have been made recently to interrogate the role of these targets through the development of chemical probes and inhibitors.^2^ Considerable work has focused on the BET family (Brd sub‐family II),^3^ however non‐BET^4^ Brds are increasingly receiving the attention of small molecule intervention efforts, with the disclosure of more than 10 new chemical probes/inhibitors in 2016.^5^

The p300/CBP‐associated factor, PCAF (KAT2B), is a multi‐domain protein containing a single Brd, an N‐terminal domain, and a histone acetyltransferase (HAT) domain. Known to associate with CBP^6^ and p3006b during transcription, misregulation of PCAF has been linked to cancer,^7^ HIV infection,7a, 8 and neuroinflammation.7a, 9 Despite predictions of high druggability^10^ and links with inflammatory disease,7a, 11 the role of PCAF and, more specifically, contributions of the Brd in such disease states are poorly understood. The development of a small molecule modulator of PCAF Brd would provide a useful tool for interrogating this potential therapeutic target and allow for dissociation of the roles of the Brd and enzymatic domains in disease. Initial reports of PCAF Brd inhibitors were focused on disrupting interactions between the HIV‐1 peptide TAT‐1 and PCAF Brd.8a,8d Wang et al. reported the first PCAF Brd inhibitor, compound **1** (PCAF IC_50_ 1.6 μm, Figure 1), which was effective at disrupting HIV‐1 replication (EC_50_ 2.8 μm).8c Further efforts made by Hu et al.^12^ towards more potent compounds such as **2** were described without significant increases in potency or indication of selectivity (PCAF IC_50_ 0.93 μm, EC_50_ 11.5 μm, Figure 1). Additional chemotypes have been disclosed from fragment‐based screening by Chaikuad et al.5l Concurrent to this work, Constellation/Genentech reported compound **3** 13 and others, which are potent PCAF inhibitors (AlphaLISA IC_50_ 13 nm) but lack reported selectivity over other Brds (Figure 1).7b,7c Despite recent developments of PCAF Brd inhibitors, a potent, selective, and cell‐active chemical probe has not been reported. The work herein describes the discovery of such a probe.

**Figure 1:**
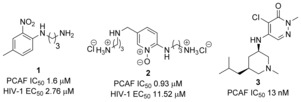
Reported PCAF bromodomain inhibitors.

Our first line of inquiry towards the first PCAF Brd chemical probe was focused on the core of non‐selective Brd inhibitors, bromosporine^14^ (PCAF isothermal titration calorimetry (ITC) *K*_D_: 5 μm) and [1,2,4]triazolo[4,3‐a]phthalazine^15^ derivatives as starting points. Small amine substituents, as in compounds **7**–**9** (Table 1), were designed to extend out of the narrow PCAF pocket and target glutamic acid residues E750 and E756 at the edge of the KAc‐binding pocket through amine–acid salt bridge interactions (PDB: 5FE0).5l Commercially available 1,4‐dichlorophthalazine **4** underwent a scalable (up to 20 g) tandem S_N_Ar/condensation reaction to furnish corresponding triazole intermediate **5** in good yields (Scheme 1). Significant efforts were employed to screen conditions using Pd‐catalyzed couplings of **5** with various amine nucleophiles; disappointing yields or lack of reactivity were observed in all of these cases. It was found that a KI/HCl‐catalyzed S_N_Ar reaction allowed for a tractable divergent synthesis of various N‐linked derivatives (Scheme 1).

**Scheme 1:**
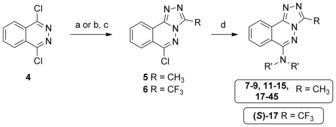
Synthesis of [1,2,4]triazolo[4,3‐a]phthalazine derivatives. Reagents and conditions: a) Acetohydrazide, DMF 120 °C 16 h, 62 %; b) N_2_H_4_ **⋅**H_2_O, EtOH, 120 °C, 10 min, *quant*.; c) TFA, 100 °C, 2 h, 43 %; d) R′_2_NH (1.5–2.0 equiv) KI (0.1 equiv), HCl (0.05 equiv), EtOH or *i*PrOH, reflux, 3 days 8–94 %.

**Table 1:** Amino‐substituted triazolophthalazine are potent PCAF Brd inhibitors. 
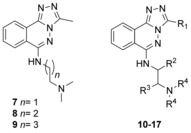

Compound	R^1^	R^2^	R^3^	R^4^	n	Δ*T*_m_ [°C]^[a]^	*K*_D_ [μm] (ITC)
**7**	Me	H	H	Me	1	8.5^[b]^	8.0±0.65
**8**	Me	H	H	Me	2	*ND*	>30
**9**	Me	H	H	Me	3	*ND*	>30
**10**	Me	H	Ph	Me	1	1.7	1.0
**11**	Me	Me	H	Me	1	5.6	0.30±0.039
**(*S*)‐11**	Me	Me	H	Me	1	7.4	0.28±0.029
**12**	Me	Et	H	Me	1	3.3	1.8±0.23
**13**	Me	*i*Bu	H	Me	1	0.85	>30
**14**	Me	Me	H	Et	1	0.0	>30
**15**	Me	H	Me	Me	1	4.6	7.3±1.1
**16**	Me	H	Et	Me	1	*ND*	6.9±1.4
**(*S*)‐17**	CF_3_	Me	H	Me	1	0.65	>30

[a] Compound concentration 10 μm, unless stated otherwise; [b] Compound concentration 100 μm; *ND*: not determined.

After the synthesis of a focused set of 20 compounds, screening conducted using a differential scanning fluorimetry (DSF) assay revealed two hits, dimethylamino compounds **7** and **10** (Table 1). It was found that compounds **8** and **9** featuring a longer amine chain were less potent. With the 2‐(dimethylamino)ethyl group of compounds **7** and **10** identified as optimal substituents, a virtual library of ∼12k compounds was constructed by in silico reaction of compound **5** with commercial compounds containing the 2‐(dimethylamino)ethyl motif.^16^ Over 60 compounds bearing a tethered 1,2‐diamine motif were chosen for synthesis based on docking score, diversity, and potential for new interactions with the PCAF Brd (Table 1, compounds **11**–**16** and Tables S1 and S2).

Derivatives were screened for PCAF Brd affinity by ITC, leading to the discovery of compound **11** (Table 1). By ITC, the stoichiometry of binding showed that all of the activity of the racemate lay in a single enantiomer, later found to have (*S*)‐configuration after synthesis using enantiopure building blocks (**11** ITC *K*_D_ 0.30 μm, Brd/**11** 2:1; (*S*)‐**11** *K*_D_ 0.28 μm, Brd/(*S*)‐**11** 1:1). Groups larger than a methyl substituent at R^2^ were detrimental to activity (compounds **12**, **13**) as was a bulkier *N*,*N*‐diethyl substituent (compound **14**). Although a phenyl substituent at R^3^ conferred potency to compound **10**, compounds **15** and **16** with smaller methyl and ethyl groups were less potent. Compound (*S*)‐**17** featuring a trifluoromethyl group at position R^1^ caused a loss in activity consistent with previously reported Brd SAR of the [1,2,4]triazolo[4,3‐a]phthalazines.^15^

In a DSF panel of 48 human Brds, compound (*S*)‐**11** showed binding to PCAF and GCN5 with no observable activity against other Brds (Figure S1). To improve the potency of (*S*)‐**11**, it was rationalized that a combination of appropriate substituents at R^2^/R^3^ might improve the avidity of binding interactions and addition of an aryl group at R^3^ would serve as a chemical handle for introduction of new functionality. The R^2^/R^3^‐substituted compounds would be a hybrid of the most potent analogues **10** and (*S*)‐**11**.

Synthesis of aryl substituted compounds was achieved through a non‐selective aza‐Henry reaction with *p*‐substituted benzaldehydes (Scheme 2). *p*‐Substituted benzaldehydes were chosen as provisional in silico scoring of potential inhibitors suggested that *o*‐ or *m*‐substitutions would be less tolerated for binding. Highly unstable olefins **18**–**24** were telescoped through a diastereoselective (d.r. 4.6:1–33:1) nitro–olefin conjugate addition furnishing racemic (S*,S*)‐configured^17^ compounds **25**–**31**, then reduced to corresponding amines, **32**–**38**, using either Pd/C‐ or Raney/Ni‐catalyzed hydrogenation. Compounds **32**–**38** were isolated as single diastereomers and submitted to the aforementioned KI‐catalyzed S_N_Ar reaction (Scheme 2) to produce compounds **39**–**45** in low to good yields (16–79 %). Racemic compounds were screened by ITC for PCAF‐binding affinity (Table 2). All of the compounds showed an increase in potency compared to compound (*S*)‐**11**, with the simple unsubstituted derivative **45** having highest potency.

**Scheme 2:**
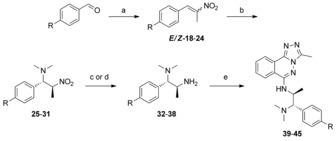
Synthesis of *threo*‐substituted derivatives **39**–**45**. Reagents and conditions: a) NH_4_OAc (0.2 equiv), EtNO_2_, reflux, 1:1 *E*/*Z*, *quant*.; b) Me_2_NH (5 equiv), THF, RT, 16 h, d.r. 4.6:1–33:1; c) H_2_ (1 atm), Pd/C (10 %), MeOH, RT, 16 h, 11–15 % over two steps, single diastereomer; d) H_2_ (1 atm), Ra/Ni (0.3 equiv), MeOH, RT, 16 h, 25–28 %, over two steps, single diastereomer; e) **5** (0.8 equiv) KI (0.1 equiv), HCl (0.05 equiv), EtOH or *i*PrOH, reflux, 3 days 16–79 %.

**Table 2:** PCAF Brd‐binding affinity of compounds **39**–**45** measured by ITC. 
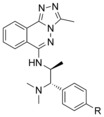

Compound	R	Configuration	*K*_D_ (nm) (ITC)
**39**	F	(1S*, 2S*)	195±23
**40**	CO_2_Me	(1S*, 2S*)	133±15
**41**	Me	(1S*, 2S*)	160±54
**42**	Cl	(1S*, 2S*)	223±78
**43**	CF_3_	(1S*, 2S*)	163±117
**44**	OMe	(1S*, 2S*)	179±48
**45**	H	(1S*, 2S*)	168±27
***L*** **‐45/*L*‐Moses**	**H**	**(1*S*, 2*S*)**	**126±15**
***D*** **‐45**	H	(1*R*, 2*R*)	Inactive

Pleasingly, it was found following resolution by preparative chiral stationary phase HPLC, that active enantiomer ***L*** **‐45**, which was dubbed ***L*** **‐Moses**, showed good binding affinity for PCAF Brd (PCAF K_*D*_ 126 nm, ITC). The other enantiomer ***D*** **‐45** showed no observable binding, implying its utility as an inactive control compound. Having achieved good potency against PCAF Brd, ***L*** **‐45** was then screened for selectivity against the panel of 48 human bromodomains using DSF (Figure 2 B). Homologous Brd of GCN5 was the only other Brd that showed any affinity for ***L*** **‐45**, confirmed by ITC (Δ*T*_m_+3.6 °C, K_*D*_ 0.55 μm). ***L*** **‐45** competitively displaced a biotinylated tool derivative, compound **46** (Supporting Information) in a homogeneous time‐resolved resonance fluorescence (HTRF) assay (PCAF K_*i*_ 47 nm), corresponding to exquisite selectivity over BRD4 (>4500‐fold selective).

**Figure 2:**
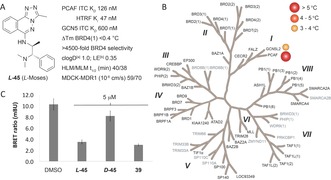
A) Profile of ***L*** **‐45**. B) ***L*** **‐45** is selective in a DSF assay panel of 48 Brds (black text). C) Displacement of PCAF‐Brd from H3.3‐nanoLuc in live HEK‐293 cells using the nanoBRET assay. [a] clogD was calculated using ChemAxon.^18^ [b] Ligand efficiency.^19^

In a cellular context, ***L*** **‐45** was shown to displace nanoLuciferase‐tagged PCAF‐Brd from halo‐tagged‐H3.3 in a nanoBRET target engagement assay at a single digit μm concentration (Figure 2 C).^20^ Inactive enantiomer ***D*** **‐45** had no effect in the same assay.

Compounds ***DL‐*** **45** and *p*‐fluoro derivative **39** were then tested for liver microsomal stability in vitro. ***DL‐*** **45** showed good metabolic stability in both human (*t*_1/2_ 40 min) and mouse (*t*_1/2_ 38 min) liver microsome preparations. *para*‐F derivative **39** showed a slightly increased metabolic stability in both human (*t*_1/2_ 48 min) and mouse (*t*_1/2_ 65 min) liver enzymes, likely due to metabolic protection of the *para*‐substituted aryl ring. ***DL‐*** **45** showed good kinetic solubility (>200 μm) and permeability in MDCK‐MDR1 cells with low efflux (Figure 2 A). ***L*** **‐45** was also tested in peripheral blood mononuclear cells and showed no observable cytotoxicity after treatment at 10 μm for 24 hours.

Although attempts to obtain a co‐crystal structure of recombinant PCAF with ***L*** **‐45** were unsuccessful, which was surprising given that numerous structures of less potent PCAF fragments have been reported recently.5l A structure using highly homologous (64 % identity) Brd from *Plasmodium falciparum*, *Pf*GCN5, of which ***L*** **‐45** is also a potent ligand (ITC K_*D*_ 280 nm), was successfully obtained (PDB: 5TPX, Figure 3). ***L*** **‐45** bound as expected in the KAc‐binding site of *Pf*GCN5 with key interactions that include a salt bridge between E1389 (conserved in PCAF as E756) and the dimethylamino motif of ***L*** **‐45** (Figure 3 A). Additional contacts are also observed in the form of an edge‐to‐face π‐π stacking interaction between W1379 (conserved in PCAF as W746) and the phenyl substituent of ***L*** **‐45** (average distance 4.5 Å); a π–π stacking interaction between Y1442 (conserved in PCAF as Y809) and pyridazo ring of the triazolophthalazine motif (average distance 3.7 Å); and characteristic H‐bonds from the triazolophthalazine group and N1436 residue (conserved in PCAF as N803) and a water molecule. Intolerance of substitution of ***L*** **‐45** in R^2^ and R^3^ positions (compounds **12**–**16,** Table 1) was rationalized by the tight fit of the alkyl amine chain of ***L*** **‐45** (Figure 3 B). Interestingly, K1383 in *Pf*GCN5 is substituted with E750 in human PCAF, and as such the *Plasmodium* homologue features a slightly open KAc‐binding site (Figure 3 B). Targeting this difference may allow for design of *Plasmodium*‐selective Brd inhibitors. As previously supported by SAR, the absolute configuration of ***L*** **‐45** was confirmed to be (1*S*,2*S*).

**Figure 3:**
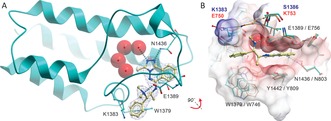
Co‐crystal structure of ***L*** **‐45** with *Pf*GCN5 (PDB ID 5TPX). A) ***L*** **‐45** (pale sticks) binds in the KAc‐binding pocket of *Pf*GCN (blue ribbon and sticks) and makes H‐bonds (dotted lines) through the triazole to N1436 and the first of a network of four water molecules (red spheres). The dimethylamino group forms a salt bridge with E1389. Blue mesh: 2 *F*_o_ *F*_c_ omitted map contoured at 2.5 σ. B) Surface view of complex of *Pf*GCN5 (surface, blue sticks) and ***L*** **‐45** (pale sticks). The phenyl group of ***L*** **‐45** lies in a hydrophobic groove between W1379 and the alkyl linker of K1383. The structure of PCAF (orange sticks, PDB ID 5FTZ) is superimposed to show key residue similarities (black text *Pf*GCN5/PCAF) and differences (blue text *Pf*GCN5, red text PCAF).

For the asymmetric synthesis of ***L*** **‐45**, commercially available (1*R*,2*S*)‐(−)‐norephedrine was Boc‐protected and cyclized to a sulfamidite and then directly oxidized using sodium periodate to boc‐protected sulfamidate **46** in reasonable yields (Scheme 3). Subsequent treatment with dimethylamine facilitated regio‐selective ring opening of sulfamidate **46**,^21^ extruding SO_3_ and furnishing protected diamine **47** as a single diastereoisomer with inversion of configuration at the benzylic centre. Following a deprotection of **47** to the free amine and S_N_Ar with aryl chloride **5**, ***L*** **‐45** was furnished in six steps as a single stereoisomer.

**Scheme 3:**
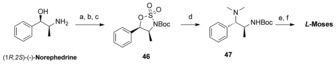
Asymmetric synthesis of ***L*** **‐45**. Reagents and conditions: a) Boc_2_O, DIPEA, CH_2_Cl_2_, RT, 16 h, 51 % b) SOCl_2_, Pyridine, MeCN, 2 h, −40 °C to 0 °C; c) NaIO_4_ (1.5 equiv), RuCl_3_⋅3 H_2_O (0.05 equiv), MeCN, 1 h, 0 °C, 48 % (over two steps); d) Me_2_NH (3 equiv), THF, RT, 16 h, 63 %; e) TFA, CH_2_Cl_2_, *quant*.; f) **5** (0.8 equiv) KI (0.1 equiv), HCl (0.05 equiv), iPrOH, reflux, 3 days, 30 %.

In conclusion, we report the discovery of ***L*** **‐45**, the first nanomolar, selective, and cell‐active chemical probe of the PCAF bromodomain. Iterative cycles of rational inhibitor design, in silico docking studies, and synthesis furnished ***L*** **‐45** after generation of a focused PCAF inhibitor library. ***L*** **‐45** shows a clean toxicity profile in primary PBMCs, and disrupts interactions between PCAF Brd and H3.3 in HEK293 cells, indicating cellular target engagement.

Good cell permeability in a MDCK‐MDR1 assay and stability to metabolism in both human and mouse liver microsomes indicate that ***L*** **‐45,** dubbed ***L*** **‐Moses**, may also have utility in vivo. ***L*** **‐Moses** will allow for robust interrogation of PCAF Brd inhibition and pharmacological effects in relevant diseases models. Future work will investigate the use of ***L*** **‐Moses** in functional assays pertaining to PCAF‐associated diseases.

## Conflict of interest

The authors declare no conflict of interest.

## Supporting information

As a service to our authors and readers, this journal provides supporting information supplied by the authors. Such materials are peer reviewed and may be re‐organized for online delivery, but are not copy‐edited or typeset. Technical support issues arising from supporting information (other than missing files) should be addressed to the authors.

Supplementary
