# Supplementary material for: Discovery of a PCAF Bromodomain Chemical Probe
Source: Angew Chem Int Ed Engl. 2016 Dec 14;56(3):827–31. doi: 10.1002/anie.201610816 (PMC5412877; doi:10.1002/anie.201610816)
Supplement: Supplementary file 1 — Supplementary [file ANIE-56-827-s001.pdf]

Angewandte Chemie
Eine Zeitschrift der Gesellschaft Deutscher Chemiker

Supporting Information

Discovery of a PCAF Bromodomain Chemical Probe

*Moses Moustakim, Peter G. K. Clark, Laura Trulli, Angel L. Fuentes de Arriba, Matthias T. Ehebauer, Apirat Chaikuad, Emma J. Murphy, Jacqui Mendez-Johnson, Danette Daniels, Chun-Feng D. Hou, Yu-Hui Lin, John R. Walker, Raymond Hui, Hongbing Yang, Lucy Dorrell, Catherine M. Rogers, Octovia P. Monteiro, Oleg Fedorov, Kilian V. M. Huber, Stefan Knapp, Jag Heer, Darren J. Dixon,** * and *Paul E. Brennan**

anie_201610816_sm_miscellaneous_information.pdf

Contents

I **General Experimental**

I.I Solvents and reagents

I.II Purification

I.III Characterization

II **Practical Experimental**

II.I **Synthetic Procedures**

II.II **Protein Expression and Purification**

II.III **Differential Scanning Calorimetry (DSF)**

Supplemental Figure 1. DSF selectivity panel of **(*S*)-11** against 48 human Brds

Supplemental Table 1. DSF results of PCAF and GCN5 with **(*S*)-11**.

Supplemental Table 2. DSF results of selected compounds with PCAF and GCN5

II.IV **Isothermal Titration Calorimetry (ITC)**

Supplementary Figure 2. ITC trace of ***L***-**-45**/***L*-Moses** and PCAF

Supplementary Figure 3. ITC trace of ***D***-**45**/***D*-Moses** and PCAF

Supplementary Figure 4. ITC trace of ***L***-**45**/***L*-Moses** and *H*GCN5

Supplementary Figure 5. ITC trace of ***L***-**45**/***L*-Moses** and *Pf*GCN5

Supplemental Table 3. Binding affinity of selected compounds with PCAF

II.V **NanoLuciferase Bioluminescent Resonance Energy Transfer (nanoBRET™) Assay**

Methods

Supplemental Figure 6. Displacement of H3.3-NanoLuc from BD-construct PCAF in live cells using the nanoBRET™ assay

II.VI ***In vitro* metabolic studies**

Supplemental Table 4. *In vitro* metabolism of compounds **45**/***DL*-Moses** and **39**.

II.VII **Cytotoxicity Studies**

Supplemental Figure 7. Cytotoxicity studies on PMBC cells of ***L*-45** and ***D*-45**.

II.VIII **High Throughput Time Resolved Fluorescence (HTRF)**

II.IX **Crystallography**

II.X **Chiral HPLC Traces**

Supplemental Figure 8. HPLC trace of ***L*-45** after asymmetric synthesis from (1*R*,2*S*)-(-)-norephedrine.

Supplemental Figure 9. HPLC trace of **45**.

Supplemental Figure 10. HPLC trace of ***L*-45** spiked with ~15% **45**.

III **Supplemental References**

I General Experimental

I.I Solvents and reagents

All solvents were purchased from commercial sources and used without purification (HPLC or analytical grade). Anhydrous solvents were purchased from Acros Organics stored under a nitrogen atmosphere with activated molecular sieves. Standard vacuum line techniques were used and glassware was flame dried prior to use. Deionised water was sourced using an Elga DV 25 system. Organic sovents were dried during workup using anhydrous Na_2SO_4.

I.II Purification and chromatography

Thin Layer Chromatography (TLC) was carried out using aluminium plates coated with 60 F_{254} silica gel. Plates were visualised using UV light (254 or 365 nm) or staining with Ninhydrin (1 M, EtOH) or 1% aq. $KMnO_4$. Normal-phase silica gel chromatography was carried out using Biotage Isolera One flash column chromatography system (LPLC). Reverse-phase high pressure liquid chromatography (RP-HPLC) was performed using a Waters system equipped with a Waters 2545 Binary Gradient Module, a SecurityGuard™ ULTRA cartridges for EVO-C18 UHPLC HPLC, Kinetex 5 µM EVO C18 100 Å 100 x 3.0 mm column and a Waters SQ Detector 2 using the stated eluent system.

I.II Characterisation

Infrared spectroscopy was carried out with a Thermo Scientific Nicolet iS5 FT-IR spectrometer fitted with an iD7-ATR accessory, selected absorption maxima (ν_{max}) recorded in wavenumbers (cm^{-1}). NMR spectra were recorded using a Bruker Avance 400 MHz spectrometer using the deuterated solvent stated. Chemical shifts (δ) quoted in parts per million (ppm) and referenced to the residual solvent peak. Multiplicities are denoted as s- singlet, d- doublet, t- triplet, q- quartet and quin- quintet and derivatives thereof (br denotes a broad resonance peak). Coupling constants recorded as Hz and round to the nearest 0.1 Hz. Two-dimensional NMR experiments (COSY, HSQC, HMBC) were used to aid the assignment of ^1H and ^{13}C spectra. Low Resolution mass spectra were recorded on a Waters SQ Detector 2 (LC-MS). High Resolution Mass Spectrometry (HRMS) was recorded using an Agilent 6530 QTOF. Melting points were obtained using a Stuart SMP40 apparatus and are reported uncorrected in °C. Optical rotations were recorded using a Perkin Elmer 341 polarimeter; absolute optical rotations are quoted as $[\alpha]_D^T$ at 23 °C, concentration (c) is reported as g/100 mL. Enantiomeric excesses were determined by HPLC analysis on an Agilent 1200 series instrument using chiral stationary phase columns. The same instrument was used in the separation of enantiomers using the stated semipreparative chiral stationary phase column. Compound names were generated using ChemBioDraw Ultra v14 systematic naming. Atom numbering in structures is purely for the purposes of assignment and does not reflect IUPAC numbering conventions.

II. Practical Experimental

II.I Synthetic Procedures

6-chloro-3-methyl-[1,2,4]triazolo[3,4-a]phthalazine (5)

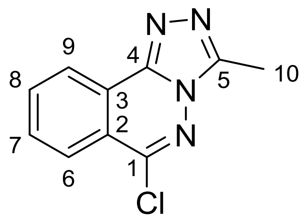

To a stirred solution of 1,4-dichlorophthalazine (18.4 g, 92 mmol, 1 eq) in DMF (anhydrous) (95 mL, 0.97 M) was added acetohydrazide (12.84 g, 139 mmol, 1.5 eq). The solution was stirred at 120 °C with a reflux condenser attached for 24 h after which TLC/LCMS analysis confirmed consumption of the starting material. The reaction mixture was cooled to 4 °C for 2 h after which an off white precipitate had formed. The precipitate was filtered off, washed with EA and dried under vacuum to afford **5** (12.478 g, 57.1 mmol, 61.7%) as an off white solid.

Mpt: 142.0-144.0 °C; **ν_{max} (cm^{-1})** 3401, 3000, 2870, 1659, 1256, 1003, 767; **^{1}H-NMR (400MHz, $CDCl_3$): δ_H** 8.69 (d, *J* = 7.8 Hz, 1H, *H*-6), 8.27 (d, *J* = 8.1 Hz, 1H, *H*-9), 8.01 (t, *J*= 8.1, 1H, *H*-8), 7.87 (t, *J*= 7.8, 1H, *H*-7); **^{13}C-NMR (100 MHz $CDCl_3$): δ_C** 149.8 (*C*-1), 147.8 (*C*-4), 142.3 (*C*-5), 134.8 (*C*-8), 131.2 (*C*-7), 127.5 (*C*-6), 124.1 (*C*-2), 123.6 (*C*-6), 122.0 (*C*-3), 9.8 (*C*-10); **LR-ESI-MS**: $C_{10}H_8ClN_4$ $[M+H]^+$ *m/z* found 219.27, cald 219.04; **HR-ESI-MS**: $C_{10}H_8ClN_4$ $[M+H]^+$ *m/z* found 219.0467, cald 219.0437.

6-chloro-3-(trifluoromethyl)-[1,2,4]triazolo[3,4-a]phthalazine (6)

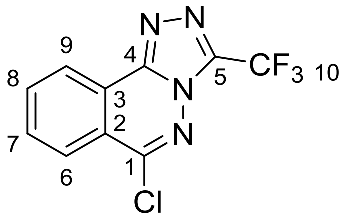

79 (860 mg, 4.42 mmol, 1 eq) was dissolved in TFA (2 mL, 26.5 mmol, 6 eq) at room temperature and stirred at 100 °C under an inert atmosphere (N_2) for 2 h. The reaction mixture was then cooled and concentrated to dryness to give a crude pink solid. The crude material was then dissolved in EA and washed with $NaHCO_3$ sat. solution (x 3). The organic layer was then washed with brine and dried over Na_2SO_4 before being concentrated and purified by Isolera Biotage LPLC (DCM/MeOH 90:10) to give **6** (519 mg, 1.903 mmol, 43%) as an off white crystalline solid.

Mpt: 170.7-172.7 °C; **ν_{max} (cm^{-1})** 1498, 1448, 1123, 1018, 962, 774, 643, 504; **^{19}F-NMR (376 MHz, $CDCl_3$): δ_F** -63.55; **^{1}H-NMR (400MHz, $CDCl_3$): δ_H** 8.79 (dd, 1H, *J*= 7.9, 0.5 Hz, *H*-9), 8.37 (d, 1H, *J*= 8.3 Hz, *H*-6), 7.83 (td, 1H, *J*= 7.7, 1.1 Hz, *H*-8), 8.01 (m, 1H, *H*-7); **^{13}C-NMR (100MHz $CDCl_3$): δ_C** 152.3 (*C*-1), 144.3 (*C*-4), 140.2 (q, *J*= 42.5 Hz, *C*-5), 135.5 (*C*-8), 132.6 (*C*-7), 127.8 (*C*-9), 124.2 (*C*-3), 123.0 (*C*-6), 122.6 (*C*-2), 117.6 (q, *J*= 271.4 Hz, *C*-10); **LR-ESI-MS:** $C_{10}H_5F_3N_4$ $[M+H]^+$ *m/z* found 272.9, cald 273.0; **HR-ESI-MS:** $C_{10}H_5F_3N_4$ $[M+H]^+$ *m/z* found 273.0172, cald 273.0155.

General Procedure A

To a stirred solution of **5** (1 eq) in EtOH (0.46 M, anhydrous) was added amine (2 eq), KI (0.1 eq) and conc. HCl (0.05 eq). The reaction mixture was then stirred at reflux for 72 h before being cooled to 4 °C at which point any precipitate formed was filtered off and washed with EA/H_2O before drying to give the desired product. In the absence of precipitated product, the reaction mixture was

concentrated to dryness and purified by Isolera Biotage LPLC (DCM/MeOH 90:10 then DCM/MeOH/NH_3 9:1:0.5) to furnish the desired compound.

N_1,N_1-dimethyl-N_2-(3-methyl-[1,2,4]triazolo[3,4-a]phthalazin-6-yl)ethane-1,2-diamine (7)

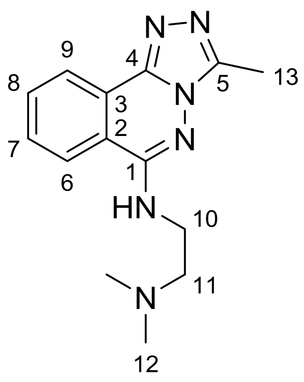

5 (100 mg, 0.457 mmol, 1 eq) and N_1,N_1-dimethylethane-1,2-diamine (81 mg, 0.915 mmol, 2 eq) were reacted according to general procedure **A**. The crude material was purified by Isolera Biotage LPLC (DCM/MeOH 90:10 then DCM/MeOH/NH_3 9:1:0.5) to give **7** (52 mg, 0.192 mmol 41.9%) as a white solid.

Mpt: 225.9-227.9 °C; **ν_{max} (cm^{-1})** 3227, 1566, 1519, 702; **^{1}H-NMR (400MHz, DMSO-*d*^{6}): δ_H** 8.34 (dd, 1H, *J*= 7.9, 0.9 Hz, *H*-9), 8.25 (d, 1H, *J*= 8.2 Hz, *H*-6), 7.88 (m, 1H, *H*-8), 7.78 (m, 1H, *H*-7), 7.50 (m, 1H, N*H*), 3.50 (m, 2H, *H*-10), 2.57 (t, 2H, *J*= 6.9 Hz, *H*-11), 2.54 (s, 3H, *H*-13), 2.22 (s, 6H, *H*-12); **^{13}C-NMR (100 MHz, DMSO-*d*^{6}): δ_C** 151.1 (*C*-1), 145.9 (*C*-4), 141.1 (*C*-5), 132.8 (*C*-8), 130.1 (*C*-7), 124.1 (*C*-9), 123.3 (*C*-3), 122.5 (*C*-6), 118.2 (*C*-2), 57.0 (*C*-10), 45.3 (*C*-11; *C*-12), 9.3 (*C*-13); **LR-ESI-MS:** $C_{14}H_{19}N_6$ [M+H]$^+$ *m/z* found 271.4, cald 271.2; **HR-ESI-MS:** $C_{14}H_{19}N_6$ [M+H]$^+$ *m/z* found 271.1682, cald 371.1671.

N_1,N_1-dimethyl-N_3-(3-methyl-[1,2,4]triazolo[3,4-a]phthalazin-6-yl)propane-1,3-diamine (8)

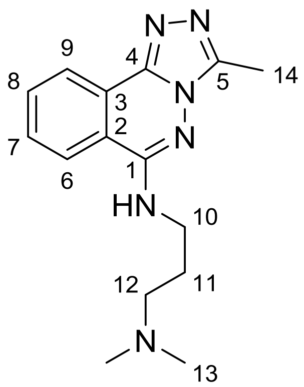

5 (200 mg, 0.915 mmol, 1 eq) and N_1,N_1-dimethylpropane-1,3-diamine (187 mg, 1.829 mmol, 2 eq) were reacted according to general procedure **A**. The crude material was purified by Isolera Biotage LPLC (DCM/MeOH 90:10 then DCM/MeOH/NH_3 9:1:0.5) to give **8** (174 mg, 0.613 mmol 67%) as a white solid.

Mpt: 118.5-120.5 °C; **ν_{max} (cm^{-1})** 3229, 2777, 1565, 1513, 1369, 1266, 1160, 783; **^{1}H-NMR (400MHz, DMSO-*d*^{6}): δ_H** 8.35 (dd, 1H, *J*= 7.9, 0.9 Hz, *H*-9), 8.23 (d, 1H, *J*= 8.1 Hz, *H*-6), 7.89 (m, 1H, *H*-8), 7.79 (td, 1H, *J*= 7.7, 1.3 Hz, *H*-7), 7.73 (m, 1H, N*H*), 3.43 (m, 2H, *H*-10), 2.54 (s, 3H, *H*-14), 2.34 (t, 2H, *H*-12), 2.17 (s, 6H, *H*-13), 1.84 (m, 2H, *H*-11); **^{13}C-NMR (100 MHz, DMSO-*d*^{6}): δ_C** 151.2 (*C*-1), 145.9 (*C*-4), 141.1 (*C*-5), 132.7 (*C*-8), 130.1 (*C*-7), 124.0 (*C*-9), 123.3 (*C*-3), 122.4 (*C*-6), 118.3 (*C*-2), 57.3 (*C*-10), 45.2 (*C*-12; *C*-13), 25.6 (*C*-11), 9.3 (*C*-14); **LR-ESI-MS:** $C_{15}H_{21}N_6$ [M+H]$^+$ *m/z* found 285.2, cald 285.2; **HR-ESI-MS:** $C_{15}H_{21}N_6$ [M+H]$^+$ *m/z* found 285.1845, cald 285.1828.

N_1,N_1-dimethyl-N_4-(3-methyl-[1,2,4]triazolo[3,4-a]phthalazin-6-yl)butane-1,4-diamine (9)

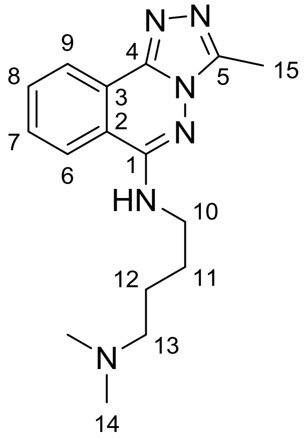

5 (200 mg, 0.915 mmol, 1 eq) and *N_1*,*N_1*-dimethylbutane-1,4-diamine (213 mg, 1.829 mmol, 2 eq) were reacted according to general procedure **A**. The crude material was purified by Isolera Biotage LPLC (DCM/MeOH 90:10 then DCM/MeOH/NH_3 9:1:0.5) to give **9** (225 mg, 0.755 mmol 83%) as a white waxy solid.

Mpt: 158.3-160.3 °C; **ν_{max} (cm^{-1})** 3261, 2865, 1591, 1512, 1476, 654; **^{1}H-NMR (400MHz, DMSO-d^6): δ_H** 8.35 (dd, J = 7.9, 0.9 Hz, 1H, *H*-9), 8.30 (d, J= 8 Hz, 1H, *H*-6), 7.89 (m, 1H *H*-8), 7.79 (td, J= 7.9, 1.3 Hz, 1H *H*-7), 7.61 (t, J= 5.2 Hz, 1H, N*H*) 3.41 (m, 2H, *H*-10), 2.55 (s, 3H, *H*-15), 2.26 (m, 2H, *H*-13), 2.12 (s, 6H, *H*-14), 1.71 (quin, J= 7.3 Hz, 2H, *H*-11), 1.51 (quin, J= 7.3 Hz, 2H, *H*-12); **^{13}C-NMR (100 MHz DMSO-d^6): δ_C** 151.2 (*C*-1), 145.9 (*C*-4), 141.1 (*C*-5), 132.7 (*C*-8), 130.1 (*C*-7), 124.2 (*C*-9), 123.3 (*C*-3), 122.4 (*C*-6), 118.3 (*C*-2), 58.7 (*C*-13), 45.0 (*C*-14), 41.2 (*C*-10), 25.7 (*C*-11), 24.7 (*C*-12), 9.3 (*C*-15); **LR-ESI-MS:** $C_{16}H_{23}N_6$ $[M+H]^+$ m/z found 299.3, cald 299.2; **HR-ESI-MS:** $C_{16}H_{23}N_6$ $[M+H]^+$ m/z found 299.2011, cald 299.1984.

N_1,N_1-dimethyl-N_2-(3-methyl-[1,2,4]triazolo[3,4-a]phthalazin-6-yl)propane-1,2-diamine (11)

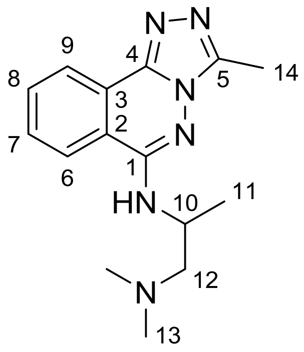

5 (200 mg, 0.915 mmol, 1 eq) and *N_1*,*N_1*-dimethylpropane-1,2-diamine (187 mg, 1.829 mmol, 2 eq) were reacted according to general procedure **A**. The crude material was purified by Isolera Biotage LPLC (DCM/MeOH 90:10 then DCM/MeOH/NH_3 9:1:0.5) to give **11** (72 mg, 0.254 mmol 27.8%) as a white solid.

Mpt: 142.3-144.3 °C; **ν_{max} (cm^{-1})** 3247, 2773, 1509, 1454, 1033, 699; **^{1}H-NMR (400MHz, DMSO-*d^6*): δ_H** 8.37 (d, 2H, *J*= 8.3 Hz, *H*-6; *H*-9), 7.91 (m, 1H, *H*-8), 7.81 (m, 1H, *H*-7), 7.18 (d, 1H, *J*=7.5 Hz, N*H*), 4.28 (m, 1H, *H*-10), 2.58 (m, 1H, *H*-12”), 2.56 (s, 3H, *H*-14), 2.27 (m, 1H, *H*-12’), 2.23 (s, 6H, *H*-13), 1.3 (d, 3H, *J*= 6.5 Hz, *H*-11); **^{13}C-NMR (100 MHz, DMSO-*d^6*):** δ_C 150.6 (*C*-1), 145.9 (*C*-4), 141.0 (*C*-5), 132.8 (*C*-8), 130.0 (*C*-7), 124.3 (*C*-9), 123.4 (*C*-3), 122.4 (*C*-6), 118.2 (*C*-2), 64.0 (*C*-10), 45.6 (*C*-13), 44.8 (*C*-12), 18.4 (*C*-11), 9.3 (*C*-14); **LR-ESI-MS:** $C_{15}H_{21}N_6$ $[M+H]^+$ *m/z* found 285.2, cald 285.2; **HR-ESI-MS:** $C_{15}H_{21}N_6$ $[M+H]^+$ *m/z* found 285.1850, cald 285.1828.

(*S*)-N_1,N_1-dimethyl-N_2-(3-methyl-[1,2,4]triazolo[3,4-a]phthalazin-6-yl)propane-1,2-diamine ((*S*)-11)

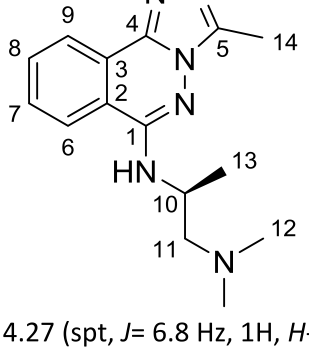

5 (300 mg, 1.372 mmol, 1 eq) and (*S*)-*N_1*,*N_1*-dimethylpropane-1,2-diamine (280 mg, 2.74 mmol, 2 eq) were reacted according to general procedure **A**. The crude material was purified by Isolera Biotage LPLC (DCM/MeOH 90:10 then DCM/MeOH/NH_3 9:1:0.5) to give **(*S*)-11** (86 mg, 0.303 mmol 22.1%) as a white solid.

Mpt: 147.4-149.4 °C; **ν_{max} (cm^{-1})** 3293, 2974, 2951, 2772, 1690, 1407, 1463, 1032, 700; **^{1}H-NMR (400MHz, DMSO-*d^6*): δ_H** 8.37 (d, *J* = 8.3 Hz, 2H, *H*-9; *H*-6), 7.91 (m, 1H, *H*-8), 7.81 (m, 1H, *H*-7), 7.17 (d, *J*= 7.3 Hz, N*H*), 4.27 (spt, *J*= 6.8 Hz, 1H, *H*-10), 2.56 (m, 1H, *H*-11”), 2.55 (s, 3H, *H*-14), 2.27 (m, 1H, *H*-11’), 2.22 (s, 6H, *H*-12), 1.3 (d, *J*= 6.5 Hz, 3H, *H*-13); **^{13}C-NMR (100 MHz DMSO-*d^6*): δ_C** 150.6 (*C*-1), 145.9 (*C*-4), 141.0

(*C*-5), 132.8 (*C*-8), 130.0 (*C*-7), 124.3 (*C*-9), 123.4 (*C*-3), 122.5 (*C*-6), 118.2 (*C*-2), 64.1 (*C*-11), 45.6 (*C*-10), 44.8 (*C*-12), 18.5 (*C*-13), 9.3 (*C*-14); **LR-ESI-MS:** $C_{15}H_{21}N_6$ $[M+H]^+$ *m/z* found 285.3, cald 285.2; **HR-ESI-MS:** $C_{15}H_{21}N_6$ $[M+H]^+$ *m/z* found 285.1829, cald 285.1828.

***N*₁,*N*₁-dimethyl-*N*₂-(3-methyl-[1,2,4]triazolo[3,4-a]phthalazin-6-yl)butane-1,2-diamine (12)**

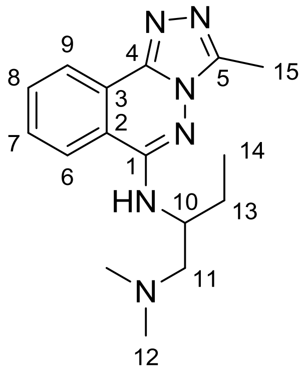

5 (400 mg, 1.829 mmol, 1 eq) and *N*₁,*N*₁-dimethylbutane-1,2-diamine (425 mg, 3.66 mmol, 2 eq) were reacted according to general procedure **A**. The crude material was purified by Isolera Biotage LPLC (DCM/MeOH 90:10 then DCM/MeOH/NH_3 9:1:0.5) to give **12** (185 mg, 0.621 mmol 33.9%) as a white solid.

Mpt: 153.5-155.5 °C; **v$_{max}$ (cm^{-1})** 3246, 2928, 2756, 1599, 1458, 1391, 1190; **^{1}H-NMR (400MHz, DMSO-*d*^{6}): δ$_H$** 8.43 (d, *J* = 8.2 Hz, 1H, *H*-9), 8.37 (d, *J*= 7.8 Hz, 1H, *H*-6), 7.92 (m, 1H, *H*-8), 7.81 (m, 1H, *H*-7), 7.11 (d, *J*= 7.8 Hz, 1H, N*H*), 4.14 (m, 1H, *H*-10), 2.55 (m, 1H, *H*-11”), 2.54 (s, 3H, *H*-15), 2.30 (m, 1H, *H*-11’), 2.22 (s, 6H, *H*-12), 1.81 (m, 1H, *H*-13”), 1.62 (m, 1H, *H*-13’), 0.94 (m, 3H, *H*-14); **^{13}C-NMR (100 MHz, DMSO-*d*^{6}): δ$_C$** 151.2 (*C*-1), 145.9 (*C*-4), 141.0 (*C*-5), 132.7 (*C*-8), 130.0 (*C*-7), 124.3 (*C*-9), 123.3 (*C*-3), 122.5 (*C*-6), 118.3 (*C*-2), 62.7 (*C*-11), 50.6 (*C*-10), 45.7 (*C*-12), 25.1 (*C*-13), 10.8 (*C*-14), 9.3 (*C*-15); **LR-ESI-MS:** $C_{16}H_{23}N_6$ $[M+H]^+$ *m/z* found 299.2, cald 299.2; **HR-ESI-MS:** $C_{16}H_{23}N_6$ $[M+H]^+$ *m/z* found 299.2001, cald 299.1984.

***N*₁,*N*₁-4-trimethyl-*N*₂-(3-methyl-[1,2,4]triazolo[3,4-a]phthalazin-6-yl)pentane-1,2-diamine (13)**

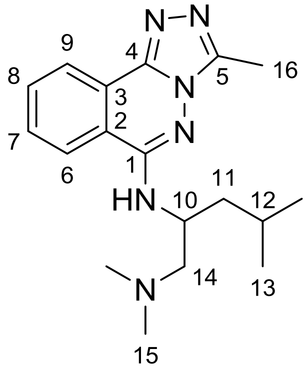

5 (300 mg, 1.372 mmol, 1 eq) and *N*₁,*N*₁,4-trimethylpentane-1,2-diamine (297 mg, 2.06 mmol, 1.5 eq) were reacted according to general procedure **A**. The crude material was purified by Isolera Biotage LPLC (DCM/MeOH 90:10 then DCM/MeOH/NH_3 9:1:0.5) to give **13** (183 mg, 0.561 mmol 40.9%) as a white solid.

Mpt: 172.4-174.4 °C; **v$_{max}$ (cm^{-1})** 3242, 2951, 2758, 1457, 1394, 1166, 1026; **^{1}H-NMR (400MHz, $CDCl_3$): δ$_H$** 8.55 (dd, *J* = 7.9, 0.7 Hz, 1H, *H*-9), 7.85 (d, *J* = 8.2 Hz, 1H, *H*-6), 7.80 (m, 1H, *H*-8), 7.68 (m, 1H, *H*-7), 5.97 (d, *J*= 4.3 Hz, 1H, N*H*), 4.17 (m, 1H, *H*-10), 2.69 (s, 3H, *H*-16), 2.66 (m, 1H, *H*-14”), 2.51 (m, 1H, *H*-14’), 2.33 (s, 6H, *H*-15), 1.84 (m, 2H, *H*-11”; *H*-12), 1.40 (m, 1H, *H*-11’), 1.04 (d, *J*= 6.4 Hz, 3H, *H*-13”), 0.97 (d, *J*= 6.5 Hz, 3H, *H*-13’); **^{13}C-NMR (100 MHz $CDCl_3$): δ$_C$** 151.1 (*C*-1), 147.2 (*C*-4), 142.0 (*C*-5), 132.4 (*C*-8), 129.9 (*C*-7), 124.2 (*C*-9), 123.6 (*C*-3), 122.7 (*C*-6), 118.8 (*C*-2), 62.9 (*C*-14), 47.5 (*C*-10), 45.6 (*C*-15), 42.6 (*C*-11), 25.1 (*C*-12), 23.3 (*C*-13”), 22.6 (*C*-13’), 9.9 (*C*-16); **LR-ESI-MS:** $C_{18}H_{27}N_6$ $[M+H]^+$ *m/z* found 327.4, cald 327.2; **HR-ESI-MS:** $C_{18}H_{27}N_6$ $[M+H]^+$ *m/z* found 327.2319, cald 327.2297.

N_1,N_1-diethyl-N_2-(3-methyl-[1,2,4]triazolo[3,4-a]phthalazin-6-yl)propane-1,2-diamine (14)

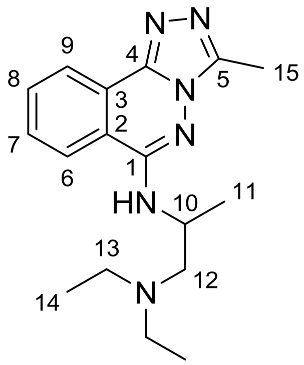

5 (400 mg, 1.829 mmol, 1 eq) and N_1,N_1-diethylpropane-1,2-diamine (477 mg, 0.571 mmol, 2 eq) were reacted according to general procedure **A**. The crude material was purified by Isolera Biotage LPLC (DCM/MeOH 90:10 then DCM/MeOH/NH_3 9:1:0.5) to give **14** (178 mg, 0.571 mmol 31.2%) as a white solid.

Mpt: 167.9-169.9 °C; **ν_{max} (cm^{-1})** 3243, 3070, 2965, 2930, 1592, 1293, 699; **^{1}H-NMR (400MHz, DMSO-*d*^{6}): δ_H** 8.36 (m, 2H, *H*-6; *H*-9), 7.90 (m, 1H, *H*-8), 7.81 (m, 1H, *H*-7), 7.15 (s, 1H, N*H*), 4.21 (m, 1H, *H*-10), 2.63 (m, 5H, *H*-12"; *H*-13), 2.54 (s, 3H, *H*-15), 2.38 (m, 1H, *H*-12'), 1.32 (d, 3H, *J*= 6.5 Hz, *H*-11), 0.97 (s, 6H, *H*-14); **^{13}C-NMR (100 MHz, DMSO-*d*^{6}): δ_C** 150.7 (*C*-1), 145.9 (*C*-4), 141.0 (*C*-5), 132.7 (*C*-8), 130.0 (*C*-7), 124.3 (*C*-9), 123.4 (*C*-3), 122.4 (*C*-6), 118.3 (*C*-2), 57.7 (*C*-10), 47.2 (*C*-13), 45.5 (*C*-12), 18.3 (*C*-11), 12.0 (*C*-14), 9.3 (*C*-15); **LR-ESI-MS:** $C_{17}H_{25}N_6$ $[M+H]^+$ *m/z* found 313.3, cald 313.2; **HR-ESI-MS:** $C_{17}H_{25}N_6$ $[M+H]^+$ *m/z* found 313.2164, cald 313.2141.

N_2,N_2-dimethyl-N_1-(3-methyl-[1,2,4]triazolo[3,4-a]phthalazin-6-yl)propane-1,2-diamine (15)

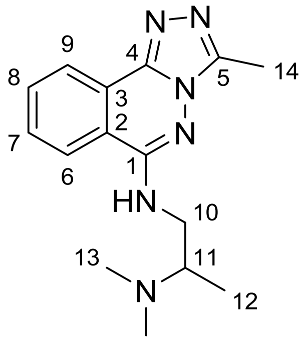

5 (200 mg, 0.915 mmol, 1 eq) and N_2,N_2-dimethylpropane-1,2-diamine (187 mg, 1.829 mmol, 2 eq) were reacted according to general procedure **A**. The crude material was purified by Isolera Biotage LPLC (DCM/MeOH 90:10 then DCM/MeOH/NH_3 9:1:0.5) to give **15** (45 mg, 0.159 mmol 17.3%) as a white solid.

Mpt: 227.1-229.1 °C; **ν_{max} (cm^{-1})** 3237, 3084, 2962, 1595, 1514, 770, 697; **^{1}H-NMR (400MHz, DMSO-*d*^{6}): δ_H** 8.36 (d, 1H, *J*= 7.8 Hz, *H*-9), 8.29 (d, 1H, *J*= 8.1 Hz, *H*-6), 7.91 (m, 1H, *H*-8), 7.81 (m, 1H, *H*-7), 7.48 (t, 1H, *J*= 4.8 Hz, N*H*), 3.56 (dd, 1H, *J*= 12.9, 6.3 Hz, *H*-10"), 3.23 (dt, 1H, *J*= 13.1, 6.4 Hz, *H*-10'), 3.02 (m, 1H, *H*-11), 2.55 (s, 3H, *H*-14), 2.26 (s, 6H, *H*-12), 1.00 (d, 1H, *J*= 6.6 Hz, *H*-12); **^{13}C-NMR (100 MHz, DMSO-*d*^{6}): δ_C** 151.1 (*C*-1), 146.0 (*C*-4), 141.0 (*C*-5), 132.8 (*C*-8), 130.2 (*C*-7), 124.1 (*C*-9), 123.3 (*C*-3), 122.5 (*C*-6), 118.2 (*C*-2), 56.6 (*C*-11), 43.9 (*C*-10), 40.4 (*C*-13), 12.3 (*C*-12), 9.3 (*C*-14); **LR-ESI-MS:** $C_{15}H_{21}N_6$ $[M+H]^+$ *m/z* found 285.2, cald 285.2; **HR-ESI-MS:** $C_{15}H_{21}N_6$ $[M+H]^+$ *m/z* found 285.1841, cald 285.1828.

N_2,N_2-dimethyl-N_1-(3-methyl-[1,2,4]triazolo[3,4-a]phthalazin-6-yl)butane-1,2-diamine (16)

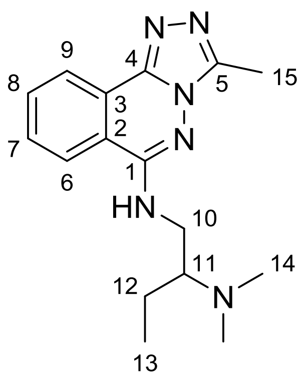

5 (200 mg, 0.915 mmol, 1 eq) and N_2,N_2-dimethylbutane-1,2-diamine (213 mg, 1.829 mmol, 2 eq) were reacted according to general procedure **A**. The crude material was purified by Isolera Biotage LPLC (DCM/MeOH 90:10 then DCM/MeOH/NH_3 9:1:0.5) to give **16** (36 mg, 0.122 mmol 13.3%) as a white solid.

Mpt: 211.5-213.5 °C; **ν_{max} (cm^{-1})** 3227, 2927, 2776, 1510, 1451, 994, 698; **^{1}H-NMR (400MHz, DMSO-*d*^{6}): δ_H** 8.37 (dd, 1H, *J*= 7.9, 0.9 Hz, *H*-9), 8.30 (d, 1H, *J*= 8.2 Hz, *H*-6), 7.91 (m, 1H, *H*-8), 7.81 (m, 1H, *H*-7), 7.52 (m, 1H, N*H*), 3.57 (m, 1H, *H*-11), 3.29 (m, 1H, *H*-10"), 2.82 (m, 1H, *H*-10'), 2.55 (s,

3H, *H*-15), 2.31 (s, 6H, *H*-14), 1.45 (m, 2H, *H*-12), 0.94 (t, 3H, *J*= 7.4 Hz, *H*-13); **^{13}C-NMR (100 MHz, DMSO-*d^6*): δ$_C$** 150.9 (*C*-1), 145.9 (*C*-4), 141.1 (*C*-5), 132.8 (*C*-8), 130.2 (*C*-7), 124.1 (*C*-9), 123.3 (*C*-3), 122.5 (*C*-6), 118.3 (*C*-2), 62.7 (*C*-11), 40.5 (*C*-10), 40.2 (*C*-14), 20.9 (*C*-12), 11.5 (*C*-13), 9.3 (*C*-15); **LR-ESI-MS:** $C_{16}H_{23}N_6$ $[M+H]^+$ *m/z* found 299.4, cald 299.2; **HR-ESI-MS:** $C_{16}H_{23}N_6$ $[M+H]^+$ *m/z* found 299.1990, cald 299.1984.

(*S*)-*N*$_1$,*N*$_1$-dimethyl-N2-(3-(trifluoromethyl)-[1,2,4]triazolo[3,4-a]phthalazin-6-yl)propane-1,2-diamine ((*S*)-17)

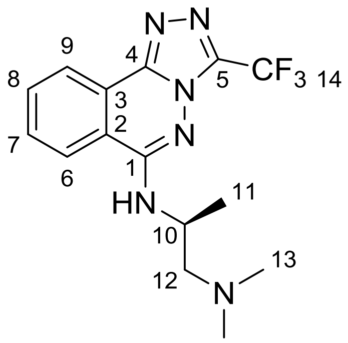

79 (85 mg, 0.312 mmol, 1 eq) and (*S*)-*N*$_1$,*N*$_1$-dimethylpropane-1,2-diamine (48 mg, 0.468 mmol, 1.5 eq) were reacted according to general procedure **A**. The crude material was purified by Isolera Biotage LPLC (DCM/MeOH 90:10 then DCM/MeOH/NH_3 9:1:0.5) to give **(*S*)-17** (67 mg, 0.199 mmol, 64%) as a white solid.

Mpt: 129.5-131.5 °C; v_{max} (cm^{-1}) 3272, 2978, 1501, 1420, 1218, 1033, 979, 720; **^{19}F-NMR (376 MHz, $CDCl_3$): δ$_F$** -64.17; **^{1}H-NMR (400MHz, $CDCl_3$): δ$_H$** 8.49 (dd, 1H, *J*= 8.1, 1 Hz, *H*-9), 7.90 (m, 2H, *H*-6; *H*-8), 7.82 (m, 1H, *H*-7), 6.52 (br-s, 1H, N*H*), 4.01 (m, 1H, *H*-10), 2.68 (m, 1H, *H*-12”), 2.42 (dd, 1H, *J*= 12.3, 4.9 Hz, *H*-12’), 2.33 (s, 6H, *H*-13), 1.41 (d, 3H, *J*= 6.1 Hz, *H*-11); **^{13}C-NMR (100MHz $CDCl_3$): δ$_C$** 152.2 (*C*-1), 143.9 (*C*-4), 139.9 (q, *J*= 40.3 Hz, *C*-5), 132.9 (*C*-8), 131.3 (*C*-7), 124.3 (*C*-9), 123.1 (*C*-3), 122.9 (*C*-6), 119.2 (*C*-2), 118.6 (q, *J*= 270.0 Hz, *C*-14), 63.9 (*C*-10), 45.3 (*C*-13), 45.2 (*C*-12), 18.1 (*C*-11); **LR-ESI-MS:** $C_{15}H_{18}F_3N_6$ $[M+H]^+$ *m/z* found 339.1, cald 339.2; **HR-ESI-MS:** $C_{15}H_{18}F_3N_6$ $[M+H]^+$ *m/z* found 339.1553, cald 339.1545.

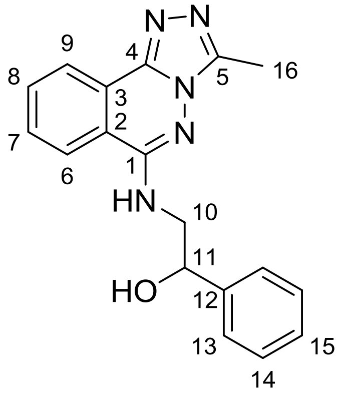

2-((3-methyl-[1,2,4]triazolo[3,4-a]phthalazin-6-yl)amino)-1-phenylethan-1-ol (96)

5 (10 g, 45.7 mmol, 1 eq) and 2-amino-1-phenylethan-1-ol (12.55 g, 91 mmol, 2 eq) were reacted according to general procedure **A**. After cooling to 4 °C a precipitate had formed which was washed according to **A** to give **96** as a white solid (9.6585 g, 30.2 mmol 66.1%).

Mpt: 172.1-174.1 °C; **v_{max} (cm^{-1})** 3309, 3061, 1515, 1265, 1052, 759, 699, 558; **^{1}H-NMR (400MHz, DMSO-*d^6*): δ$_H$** 8.37 (m, 2H, *H*-6; *H*-9), 7.92 (m, 1H, *H*-8), 7.84 (m, 2H, *H*-7; N*H*), 7.46 (d, *J*= 7.3 Hz, 2H, *H*-13), 7.35 (m, 2H, *H*-14), 7.25 (m, 1H, *H*-15), 5.55 (d, *J*= 4.3 Hz, 1H, O*H*), 5.12 (dt, *J*= 8.2, 4, 4 Hz, 1H, *H*-11), 3.71 (m, 1H, *H*-10”), 3.39 (m, 1H, *H*-10’), 2.60 (s, 3H, *H*-16); **^{13}C-NMR (100 MHz DMSO-*d^6*): δ$_C$** 151.1 (*C*-1), 145.9 (*C*-4), 144.1 (*C*-12), 141.1 (*C*-5), 132.8 (*C*-8), 130.2 (*C*-7), 128.1 (*C*-14), 126.9 (*C*-15), 125.8 (*C*-13), 124.3 (*C*-9), 123.3 (*C*-3), 122.4 (*C*-6), 118.3 (*C*-2), 69.4 (*C*-11), 50.0 (*C*-10), 9.4 (*C*-16); **LR-ESI-MS:** $C_{18}H_{18}N_5O$ $[M+H]^+$ *m/z* found 320.4, cald 320.2; **HR-ESI-MS:** $C_{18}H_{18}N_5O$ $[M+H]^+$ *m/z* found 320.1041, cald 320.1511.

3-methyl-*N*-((1-methylpyrrolidin-2-yl)methyl)-[1,2,4]triazolo[3,4-a]phthalazin-6-amine (97)

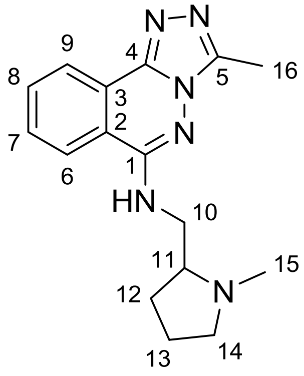

5 (200 mg, 0.915 mmol, 1 eq) and (1-methylpyrrolidin-2-yl)methanamine (209 mg, 1.829 mmol, 2 eq) were reacted according to general procedure **A**. The crude material was purified by Isolera Biotage LPLC (DCM/MeOH 90:10 then DCM/MeOH/NH_3 9:1:0.5) to give **97** (221 mg, 0.746 mmol 82%) as an off white powder.

Mpt: 248.6-250.6 °C; v_{max} (cm^{-1}) 3223, 3078, 2964, 2934, 2768, 1452, 698; **^{1}H-NMR (400MHz, DMSO-*d^6*): δ_H** 8.34 (m, 2H, *H*-9; *H*-6), 7.92 (t *J*= 7.2 Hz, 1H, *H*-8), 7.82 (t, *J*= 7.2 Hz, 1H, *H*-7), 7.63 (s, 1H, N*H*), 3.68 (m, 1H, *H*-10'), 3.23 (m, 1H, *H*-10"), 3.17 (m, 1H, *H*-11), 2.98 (m, 2H, *H*-14), 2.56 (s, 3H, *H*-16), 2.41 (m, 3H, *H*-15), 2.16 (m, 1H, *H*-13'), 1.92 (m, 1H, *H*-12'), 1.66 (m, 2H, *H*-12"; *H*-13"); **^{13}C-NMR (100 MHz DMSO-*d^6*): δ_C** 151.2 (*C*-1), 145.9 (*C*-4), 141.1 (*C*-5), 132.8 (*C*-8), 130.2 (*C*-7), 124.1 (*C*-9), 123.3 (*C*-3), 122.5 (*C*-6), 118.2 (*C*-2), 63.1 (*C*-10), 56.9 (*C*-11), 45.1 (*C*-14), 40.9 (*C*-15), 29.6 (*C*-12), 22.4 (*C*-13), 9.3 (*C*-16); **LR-ESI-MS:** $C_{16}H_{21}N_6$ $[M+H]^+$ *m/z* found 297.4, cald 297.2; **HR-ESI-MS:** $C_{16}H_{21}N_6$ $[M+H]^+$ *m/z* found 297.1852, cald 297.1828.

3-methyl-*N*-(2-morpholinoethyl)-[1,2,4]triazolo[3,4-a]phthalazin-6-amine (98)

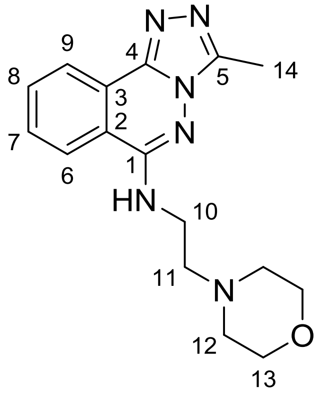

5 (200 mg, 0.915 mmol, 1 eq) and 2-morpholinoethan-1-amine (238 mg, 1.829 mmol, 2 eq) were reacted according to general procedure **A**. After cooling to 4 °C a precipitate had formed which was washed according to **A** to give **98** (58 mg, 0.185 mmol, 20.3%) as an off white powder.

Mpt: 169.7-171.7 °C; **v_{max} (cm^{-1})** 3219, 3071, 2946, 1594, 1552, 1112, 771; **^{1}H-NMR (400MHz, DMSO-*d^6*): δ_H** 8.36 (d, *J* = 8.6 Hz, 1H, *H*-9), 8.26 (d *J*= 8.1 Hz, 1H, *H*-6), 7.90 (t, *J*= 7.2 Hz, 1H, *H*-8), 7.80 (m, 1H, *H*-7), 7.56 (t, *J*= 5.3 Hz, 1H, N*H*), 3.58 (m, 6H, *H*-10; *H*-13), 2.64 (t, *J*=7 Hz, 2H, *H*-11), 2.55 (s, 3H, *H*-14), 2.48 (m, 4H, *H*-12); **^{13}C-NMR (100 MHz DMSO-*d^6*): δ_C** 151.1 (*C*-1), 146.0 (*C*-4), 141.1 (*C*-5), 132.8 (*C*-8), 130.2 (*C*-7), 124.1 (*C*-9), 123.3 (*C*-3), 122.5 (*C*-6), 118.2 (*C*-2), 66.3 (*C*-13), 56.3 (*C*-12), 53.4 (*C*-11), 38.4 (*C*-10), 9.3 (*C*-14); **LR-ESI-MS:** $C_{16}H_{21}N_6O$ $[M+H]^+$ *m/z* found 313.4, cald 313.2; **HR-ESI-MS:** $C_{16}H_{21}N_6O$ $[M+H]^+$ *m/z* found 313.1812, cald 313.1777.

3-methyl-*N*-((tetrahydro-2H-pyran-3-yl)methyl)-[1,2,4]triazolo[3,4-a]phthalazin-6-amine (99)

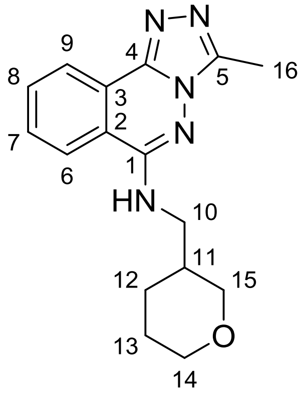

5 (100 mg, 0.457 mmol, 1 eq) and (tetrahydro-2H-pyran-3-yl)methanamine (105 mg, 0.915 mmol, 2 eq) were reacted according to general procedure **A**. The crude material was purified by Isolera Biotage LPLC (DCM/MeOH 90:10 then DCM/MeOH/NH_3 9:1:0.5) to give **99** (41 mg, 0.138 mmol 30.1%) as an off white powder.

Mpt: 156.6-158.6 °C; **v_{max} (cm^{-1})** 3250, 3923, 2849, 1567, 1483, 770; **^{1}H-NMR (400MHz, DMSO-*d^6*): δ_H** 8.35 (t, *J* = 8.6 Hz, 2H, *H*-6; *H*-9), 7.9 (m, 1H,

H-8), 7.8 (m, 1H, *H*-7), 7.6 (t, *J*= 5.3 Hz, 1H, N*H*), 3.87 (m, 1H, *H*-15”), 3.72 (m, 1H, *H*-15’), 3.33 (m, 1H, *H*-14”), 3.21 (m, 1H, *H*-14’), 2.55 (s, 3H, *H*-16), 2.09 (m, 1H, *H*-13”), 1.84 (m, 1H, *H*-13’), 1.61 (m, 1H, *H*-12”), 1.49 (m, 1H, *H*-12’), 1.33 (m, 1H, *H*-11); **^{13}C-NMR (100 MHz DMSO-*d*^{6}): δ$_C$** 151.3 (*C*-1), 146.0 (*C*-4), 141.1 (*C*-5), 132.7 (*C*-8), 130.1 (*C*-7), 124.2 (*C*-9), 123.3 (*C*-3), 122.4 (*C*-6), 118.2 (*C*-2), 70.9 (*C*-15), 67.5 (*C*-14), 54.9 (*C*-10), 34.8 (*C*-11), 27.4 (*C*-12), 24.9 (*C*-13), 9.3 (*C*-16); **LR-ESI-MS:** $C_{19}H_{20}N_2O$ [M+H]$^+$ *m/z* found 298.4, cald 298.2; **HR-ESI-MS:** $C_{19}H_{20}N_2O$ [M+H]$^+$ *m/z* found 298.1703, cald 298.1668.

1-methyl-3-((3-methyl-[1,2,4]triazolo[3,4-a]phthalazin-6-yl)amino)piperidin-2-one (50)

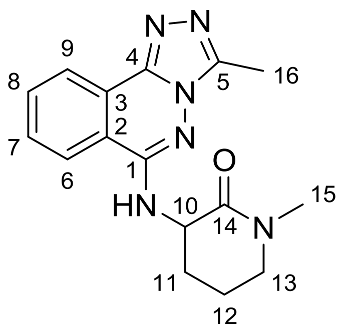

5 (100 mg, 0.457 mmol, 1 eq) and 3-amino-1-methylpiperidin-2-one (117 mg, 0.915 mmol, 2 eq) were reacted according to general procedure **A**. The crude material was purified by Isolera Biotage LPLC (DCM/MeOH 90:10 then DCM/MeOH/NH_3 9:1:0.5) to give **50** (53 mg, 0.172 mmol 37.5%) as a white solid.

Mpt: 229.5-231.5 °C; **ν$_{max}$ (cm^{-1})** 3259, 3077, 2941, 1595, 1504, 1270, 658; **^{1}H-NMR (400MHz, DMSO-*d*^{6}): δ$_H$** 8.34 (d J= 7.2 Hz, 1H, *H*-6), 8.28 (d, J= 8.2 Hz, 1H, *H*-9), 7.99 (d, J= 7.3 Hz, 1H, N*H*), 7.87 (t, J= 7.6 Hz, 1H, *H*-8), 7.76 (m, 1H, *H*-7), 4.36 (dt, J= 11.2, 6.6 Hz, 1H, *H*-10), 3.43 (m, 1H, *H*-13”), 3.37 (s, 3H, *H*-15), 3.35 (m, 1H, *H*-13’), 2.89 (s, 3H, *H*-16), 2.22 (m, 1H, *H*-12”), 2.09 (m, 1H, *H*-12’), 1.92 (m, 2H, *H*-11); **^{13}C-NMR (100 MHz DMSO-*d*^{6}): δ$_C$** 168.4 (*C*-14), 150.3 (*C*-1), 145.8 (*C*-4), 141.0 (*C*-5), 132.8 (*C*-8), 130.1 (*C*-7), 124.2 (*C*-9), 123.3 (*C*-3), 122.4 (*C*-6), 118.2 (*C*-2), 51.8 (*C*-10), 49.5 (*C*-15), 34.4 (*C*-13), 26.5 (*C*-11), 21.3 (*C*-12), 9.3 (*C*-16); **LR-ESI-MS:** $C_{16}H_{19}N_6O$ [M+H]$^+$ m/z found 311.4, cald 311.2; **HR-ESI-MS:** $C_{16}H_{19}N_6O$ [M+H]$^+$ m/z found 311.1663, cald 311.1620.

2-((3-methyl-[1,2,4]triazolo[3,4-a]phthalazin-6-yl)amino)-2-phenylethan-1-ol (51)

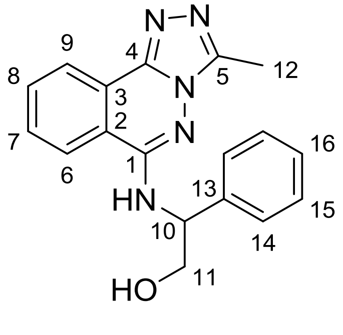

5 (100 mg, 0.457 mmol, 1 eq) and 2-amino-2-phenylethan-1-ol (125 mg, 0.915 mmol, 2 eq) were reacted according to general procedure **A**. After cooling to 4 °C a precipitate had formed which was washed according to **A** to give **51** (43 mg, 0.133 mmol, 29%).

Mpt: 171.3-173.3 °C; **ν$_{max}$ (cm^{-1})** 3215, 2924, 1544, 1474, 1036, 760, 773; **^{1}H-NMR (400MHz, DMSO-*d*^{6}): δ$_H$** 8.56 (d, *J* = 7.9 Hz, 1H, *H*-6), 8.36 (d, *J* = 8.9 Hz, 1H, *H*-9), 7.88 (m, 3H, N*H*; *H*-8 & *H*-7), 7.49 (d, *J*= 7.2 Hz, 2H, *H*-14), 7.31 (t, *J*=7.5 Hz, 2H, *H*-15), 7.21 (m, 1H, *H*-16), 5.12 (m, 1H, *H*-10), 5.04 (t, *J*= 5.8 Hz, 1H, O*H*), 3.91 (ddd, *J*= 11.1, 8.4, 5.7 Hz, 1H, *H*-11”) 3.73 (dt, *J*= 11.1, 5.5 Hz, 1H, *H*-11’), 2.45 (s, 3H, *H*-12); **^{13}C-NMR (100 MHz DMSO-*d*^{6}): δ$_C$** 150.7 (*C*-1), 145.9 (*C*-4), 141.4 (*C*-5), 140.9 (*C*-13), 132.9 (*C*-8), 130.1 (*C*-7), 128.0 (*C*-15), 127.3 (*C*-14), 126.9 (*C*-16), 124.6 (*C*-6), 123.3 (*C*-3), 122.5 (*C*-9), 118.2 (*C*-2), 64.5 (*C*-10), 58.3 (*C*-11), 9.2 (*C*-12); **LR-ESI-MS:** $C_{18}H_{18}N_5O$ [M+H]$^+$ *m/z* found 320.4, cald 320.2; **HR-ESI-MS:** $C_{18}H_{18}N_5O$ [M+H]$^+$ *m/z* found 320.1530, cald 320.1511.

N-((1-ethylpyrrolidin-2-yl)methyl)-3-methyl-[1,2,4]triazolo[3,4-a]phthalazin-6-amine (52)

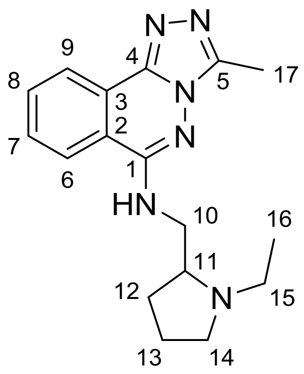

5 (200 mg, 0.915 mmol, 1 eq) and (1-ethylpyrrolidin-2-yl)methanamine (235 mg, 1.829 mmol, 2 eq) were reacted according to general procedure **A**. The crude material was purified by Isolera Biotage LPLC (DCM/MeOH 90:10 then DCM/MeOH/NH_3 9:1:0.5) to give **52** (138 mg, 0.445 mmol 48.6%) as a yellow solid.

Mpt: 219.4-221.4 °C; **ν_{max} (cm^{-1})** 3227, 3070, 2960, 2772, 1482, 779; **^{1}H-NMR (400MHz, DMSO-d^6): δ_H** 8.36 (d, *J* = 7.8 Hz, 1H, *H*-9), 8.3 (d, *J*= 8.1 Hz, 1H, *H*-6), 7.9 (m, 1H, *H*-8), 7.8 (m, 1H, *H*-7), 7.67 (m, 1H, N*H*), 4.11 (s, 1H, *H*-10''), 3.63 (m, 1H, *H*-10'), 3.17 (m, 2H, *H*-15), 3.07 (m, 1H, *H*-11), 2.99 (m, 1H, *H*-14''), 2.91 (m, 1H, *H*-14'), 2.54 (s, 3H, *H*-17), 2.38 (m, 1H, *H*-12''), 2.18 (m, 1H, *H*-12'), 1.85 (m, 1H, *H*-13''), 1.68 (m, 1H, *H*-13'), 1.09 (t, *J*= 7.2 Hz, 3H, *H*-16); **^{13}C-NMR (100 MHz CDCl$_3$): δ_C** 151.2 (*C*-1), 145.9 (*C*-4), 141.1 (*C*-5), 132.8 (*C*-8), 130.2 (*C*-7), 124.1 (*C*-9), 123.3 (*C*-3), 122.5 (*C*-6), 118.2 (*C*-2), 61.2 (*C*-11), 53.3 (*C*-14), 48.6 (*C*-15), 48.3 (*C*-10), 29.1 (*C*-12), 22.5 (*C*-13), 13.8 (*C*-16), 9.3 (*C*-17); **LR-ESI-MS:** $C_{17}H_{23}N_6$ $[M+H]^+$ *m/z* found 311.4, cald 311.2; **HR-ESI-MS:** $C_{17}H_{23}N_6$ $[M+H]^+$ *m/z* found 311.2000, cald 311.1984.

(*S*)-*N*-(1-benzylpiperidin-3-yl)-3-methyl-[1,2,4]triazolo[3,4-a]phthalazin-6-amine (56)

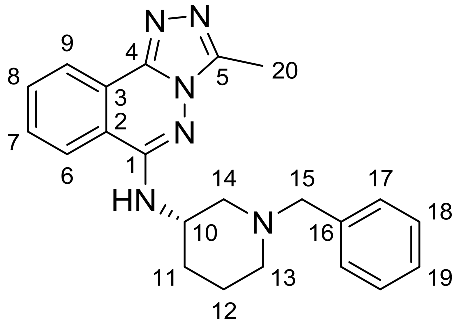

5 (400 mg, 1.829 mmol, 1 eq) and (S)-1-benzylpiperidin-3-amine (522 mg, 2.74 mmol, 1.5 eq) were reacted according to general procedure **A**. The crude material was purified by Isolera Biotage LPLC (DCM/MeOH 90:10 then DCM/MeOH/NH_3 9:1:0.5) to give **56** (200 mg, 0.537 mmol 29.4%) as a yellow solid.

Mpt: 132.6-134.6 °C; **ν_{max} (cm^{-1})** 3336, 2930, 2795, 1590, 1513; **^{1}H-NMR (400MHz, DMSO-d^6): δ_H** 8.34 (m, 2H, *H*-9; *H*-6), 7.88 (m, 1H, *H*-8), 7.78 (td, *J* = 7.7, 1.3 Hz, 1H, *H*-8), 7.31 (m, 4H, *H*-17; *H*-18), 7.21 (m, 1H, *H*-19), 7.12 (d, *J*= 7.3 Hz, 1H, N*H*), 4.05 (m, 1H, *H*-10), 3.62 (d, *J*= 13.3 Hz, 1H, *H*-15''), 3.39 (d, *J*= 13.3 Hz, 1H, *H*-15'), 3.16 (m, 1H, *H*-14''), 2.81 (m, 1H, *H*-14'), 2.43 (s, 3H, *H*-20), 2.02 (m, 2H, *H*-13), 1.86 (t, *J*= 10 Hz, 1H, *H*-12''), 1.74 (m, 1H, *H*-11''), 1.62 (m, 1H, *H*-11'), 1.5 (m, 1H, *H*-12'); **^{13}C-NMR (100 MHz, DMSO-d^6): δ_C** 150.4 (*C*-1), 145.9 (*C*-4), 141.0 (*C*-5), 138.5 (*C*-16), 132.8 (*C*-8), 130.0 (*C*-7), 128.7 (*C*-17), 128.1 (*C*-18), 126.8 (*C*-19), 124.4 (*C*-9), 123.3 (*C*-3), 122.4 (*C*-6), 118.1 (*C*-2), 62.1 (*C*-15), 56.9 (*C*-14), 53.6 (*C*-13), 48.3 (*C*-10), 29.5 (*C*-11), 24.1 (*C*-12), 9.2 (*C*-20); **LR-ESI-MS:** $C_{22}H_{25}N_6$ $[M+H]^+$ *m/z* found 373.3, cald 373.2; **HR-ESI-MS:** $C_{22}H_{25}N_6$ $[M+H]^+$ *m/z* found 373.2173, cald 373.2141.

1-(3-methoxyphenyl)-4-((3-methyl-[1,2,4]triazolo[3,4-a]phthalazin-6-yl)amino)pyrrolidin-2-one (57)

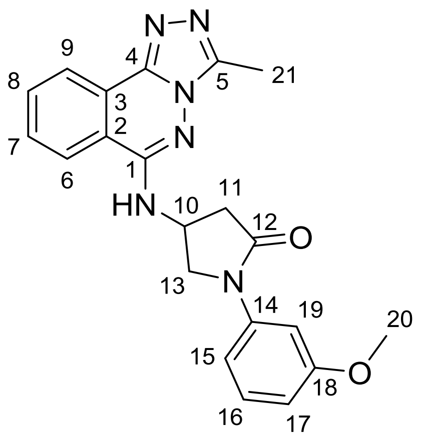

5 (400 mg, 1.829 mmol, 1 eq) and 4-amino-1-(3-methoxyphenyl)pyrrolidin-2-one (566 mg, 2.74 mmol, 1.5 eq) were reacted according to general procedure **A**. The crude material was purified by Isolera Biotage LPLC (DCM/MeOH 90:10 then DCM/MeOH/NH_3 9:1:0.5) to give **57** (61 mg, 0.157 mmol 8.6%) as a brown solid.

Mpt: 249.9-251.1 °C; **v_{max} (cm^{-1})** 3250, 2929, 1697, 1592, 1498, 1317, 1212, 1043, 765, 701; **^{1}H-NMR (400MHz, DMSO-*d*^{6}): δ_H** 8.34 (m, 2H, *H*-6; *H*-9), 7.88 (m, 2H, *H*-7; *H*-8), 7.76 (m, 1H, N*H*), 7.74 (m, 1H, *H*-19), 7.26 (m, 1H, *H*-16), 7.20 (m, 1H, *H*-15), 6.71 (dt, 1H, *J*= 8, 1.2 Hz, *H*-17), 4.67 (s, 1H, *H*-10), 4.30 (dd, 1H, *J*= 10.4, 7.1 Hz, *H*-13”), 3.97 (dd, 1H, *J*= 10.5, 3.2 Hz, *H*-13’), 3.73 (s, 3H, *H*-20), 3.10 (dd, 1H, *J*= 17.4, 8.4 Hz, *H*-11”), 2.81 (dd, 1H, *J*= 17.5, 3.9 Hz, *H*-11’), 2.55 (s, 3H, *H*-21); **^{13}C-NMR (100 MHz, DMSO-*d*^{6}): δ_C** 172.17 (*C*-12), 159.4 (*C*-18), 150.6 (*C*-1), 146.1 (*C*-4), 141.0 (*C*-5), 140.5 (*C*-14), 132.8 (*C*-8), 129.9 (*C*-7), 129.4 (*C*-19, 124.4 (*C*-9), 123.3 (*C*-3), 122.4 (*C*-6), 118.1 (*C*-2), 111.6 (*C*-16), 109.2 (*C*-15), 105.6 (*C*-17), 55.1 (*C*-20), 53.9 (*C*-10), 44.1 (*C*-11), 38.6 (*C*-13), 9.3 (*C*-21); **LR-ESI-MS:** $C_{21}H_{21}N_6O_2$ $[M+H]^+$ *m/z* found 389.2, cald 389.1; **HR-ESI-MS:** $C_{21}H_{21}N_6O_2$ $[M+H]^+$ *m/z* found 389.1742, cald 389.1726.

3-methyl-*N*-((4-methylmorpholin-3-yl)methyl)-[1,2,4]triazolo[3,4-a]phthalazin-6-amine (58)

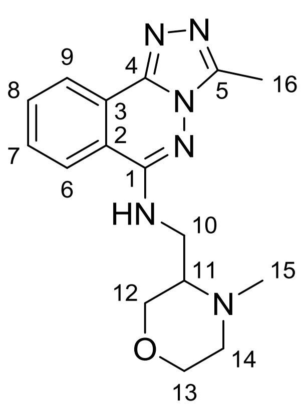

5 (200 mg, 0.915 mmol, 1 eq) and (4-methylmorpholin-3-yl)methanamine (238 mg, 1.829 mmol, 1.5 eq) were reacted according to general procedure **A**. The crude material was purified by Isolera Biotage LPLC (DCM/MeOH 90:10 then DCM/MeOH/NH_3 9:1:0.5) to give **58** (97 mg, 0.310 mmol 33.9%) as a white solid.

Mpt: 172.7-174.7 °C; **v_{max} (cm^{-1})** 3224, 3069, 2924, 2857, 2792, 1565, 1513, 1124, 699; **^{1}H-NMR (400MHz, DMSO-*d*^{6}): δ_H** 8.36 (d, 1H, *J*= 7.7 Hz, *H*-9), 8.29 (d, 1H, *J*= 8.1 Hz, *H*-6), 7.91 (t, 1H, *J*= 7.6 Hz, *H*-8), 7.80 (m, 1H, *H*-7), 7.61 (t, 1H, *J*= 5.4 Hz, N*H*), 3.80 (m, 2H, *H*-12”; *H*-13”), 3.66 (m, 1H, *H*-12’), 3.52 (m, 1H, *H*-13’), 3.33 (m, 1H, *H*-10”), 3.19 (m, 1H, *H*-10’), 2.66 (m, 1H, *H*-11), 2.56 (s, 3H, *H*-16), 2.51 (m, 1H, *H*-14”), 2.40 (s, 3H, *H*-15), 2.21 (m, 1H, *H*-14’); **^{13}C-NMR (100 MHz, DMSO-*d*^{6}): δ_C** 151.1 (*C*-1), 145.9 (*C*-4), 141.1 (*C*-5), 132.9 (*C*-8), 130.2 (*C*-7), 124.2 (*C*-9), 123.3 (*C*-3), 122.5 (*C*-6), 118.2 (*C*-2), 69.6 (*C*-12), 66.2 (*C*-13), 60.3 (*C*-11), 54.4 (*C*-14), 42.8 (*C*-10), 39.5 (*C*-15), 9.3 (*C*-16); **LR-ESI-MS:** $C_{16}H_{21}N_6O$ $[M+H]^+$ *m/z* found 313.2, cald 313.2; **HR-ESI-MS:** $C_{16}H_{21}N_6O$ $[M+H]^+$ *m/z* found 313.1797, cald 313.1777.

3-methyl-*N*-(2-(pyrrolidin-1-yl)ethyl)-[1,2,4]triazolo[3,4-a]phthalazin-6-amine acetate (59)

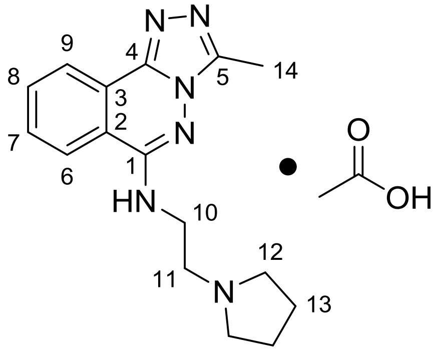

5 (200 mg, 0.915 mmol, 1 eq) and 2-(pyrrolidin-1-yl)ethan-1-amine (209 mg, 1.829 mmol, 1.5 eq) were reacted according to general procedure **A**. After cooling to 4 °C a precipitate had formed which was washed according to **A** and then co-evaporated with AcOH to give **59** as an AcOH salt (101 mg, 0.283 mmol, 31%).

Mpt: 172.4-174.4 °C; **ν_{max} (cm^{-1})** 3449, 3261, 2693, 2615, 1516, 701; **^{1}H-NMR (400MHz, MeOD-*d^{4}*): δ_H** 8.33 (d, 1H, *J*= 7.9 Hz, *H*-9), 8.18 (d, 1H, *J*= 8.2 Hz, *H*-6), 7.89 (m, 1H, *H*-8), 7.81 (m, 1H, *H*-7), 3.96 (t, 2H, *J*= 5.9 Hz, *H*-10), 3.69 (t, 2H, *J*= 5.9 Hz, *H*-11), 3.50 (br s, 4H, *H*-12), 2.64 (s, 3H, *H*-14), 2.16 (s, 4H, *H*-13); **^{13}C-NMR (100 MHz, MeOD-*d^{4}*): δ_C** 152.1 (*C*-1), 147.2 (*C*-4), 141.8 (*C*-5), 132.9 (*C*-8), 130.8 (*C*-7), 123.8 (*C*-9), 122.7 (*C*-3), 122.6 (*C*-6), 118.6 (*C*-2), 54.1 (*C*-12), 52.9 (*C*-11), 37.7 (*C*-10), 22.6 (*C*-13), 8.1 (*C*-14); **LR-ESI-MS:** $C_{16}H_{21}N_6$ [M+H]$^+$ *m/z* found 297.2, cald 297.2; **HR-ESI-MS:** $C_{16}H_{21}N_6$ [M+H]$^+$ *m/z* found 297.1852, cald 297.1828.

3-methyl-*N*-((1-methylpiperidin-2-yl)methyl)-[1,2,4]triazolo[3,4-a]phthalazin-6-amine (60)

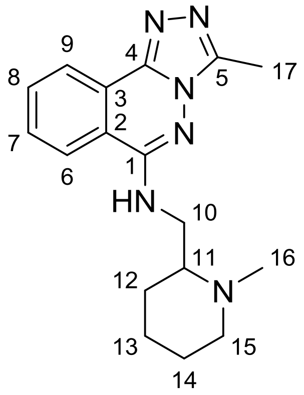

5 (200 mg, 0.915 mmol, 1 eq) and (1-methylpiperidin-2-yl)methanamine (235 mg, 1.829 mmol, 1.5 eq) were reacted according to general procedure **A**. The crude material was purified by Isolera Biotage LPLC (DCM/MeOH 90:10 then DCM/MeOH/NH_3 9:1:0.5) to give **60** (147 mg, 0.475 mmol 51.9%) as a white solid.

Mpt: 171.6-173.6 °C; **ν_{max} (cm^{-1})** 3231, 3071, 2928, 1565, 1511, 1267, 698; **^{1}H-NMR (400MHz, DMSO-*d^{6}*): δ_H** 8.37 (dd, 1H, *J*= 7.9, 1 Hz, *H*-9), 8.31 (d, 1H, *J*= 8.2 Hz, *H*-6), 7.91 (m, 1H, *H*-8), 7.81 (td, 1H, *J*= 7.8, 1.3 Hz, *H*-7), 7.56 (t, 1H, *J*= 5 Hz, N*H*), 4.11 (s, 1H, *H*-11), 3.79 (m, 1H, *H*-10”), 3.26 (m, 1H, *H*-10’), 2.80 (d, 1H, *J*= 11.2 Hz, *H*-12”), 2.55 (s, 3H, *H*-17), 2.38 (s, 3H, *H*-16), 2.09 (m, 1H, *H*-12’), 1.8-1.1 (m, 6H, *H*-13; *H*-14; *H*-15); **^{13}C-NMR (100 MHz, DMSO-*d^{6}*): δ_C** 151.1 (*C*-1), 145.9 (*C*-4), 141.1 (*C*-5), 132.8 (*C*-8), 130.2 (*C*-7), 124.1 (*C*-9), 123.3 (*C*-3), 122.5 (*C*-6), 118.2 (*C*-2), 61.4 (*C*-11), 56.1 (*C*-15), 54.9 (*C*-10), 48.6 (*C*-16), 43.8 (*C*-12), 25.1 (*C*-14), 23.3 (*C*-13), 9.3 (*C*-17); **LR-ESI-MS:** $C_{17}H_{23}N_6$ [M+H]$^+$ *m/z* found 311.4, cald 311.2; **HR-ESI-MS:** $C_{17}H_{23}N_6$ [M+H]$^+$ *m/z* found 311.2006, cald 311.1984.

***N*-((1*S**, 2*S**, 4*R**)-bicyclo[2.2.1]heptan-2-yl)-3-methyl-[1,2,4]triazolo[3,4-a]phthalazin-6-amine (61)**

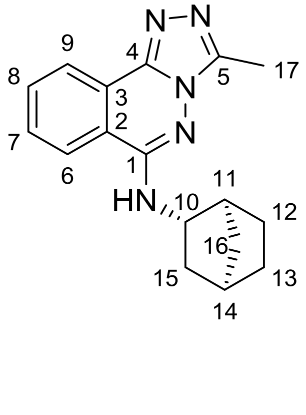

5 (100 mg, 0.457 mmol, 1 eq) and *rac*-(1*S*,2*S*,4*R*)-bicyclo[2.2.1]heptan-2-amine (102 mg, 0.915 mmol, 2 eq) were reacted according to general procedure **A**. The crude material was purified by Isolera Biotage LPLC (DCM/MeOH 90:10 then DCM/MeOH/NH_3 9:1:0.5) to give **61** (20 mg, 0.068 mmol 14.9%) as a white crystalline solid.

Mpt: 256.2-258.2 °C; **ν_{max} (cm^{-1})** 3273, 2944, 1502, 769; **^{1}H-NMR (400MHz, DMSO-*d^{6}*): δ_H** 8.48 (d, 1H, *J*= 8.2 Hz, *H*-9), 8.35 (dd, 1H, *J*= 7.9,

0.8 Hz, *H*-6), 7.89 (m, 1H, *H*-8), 7.79 (m, 1H, *H*-7), 7.13 (d, 1H, *J*= 5.5 Hz, N*H*), 3.75 (m, 1H, *H*-10), 2.55 (s, 3H, *H*-17), 2.46 (m, 1H, *H*-11), 2.27 (m, 1H, *H*-14), 1.8-1.4 (m, 5H, *H*-12"; *H*-15; *H*-16), 1.3-1.1 (m, 3H, *H*-12'; *H*-13); **^{13}C-NMR (100 MHz, DMSO-*d^6*): δ_C** 150.5 (*C*-1), 145.9 (*C*-4), 140.9 (*C*-5), 132.6 (*C*-8), 129.9 (*C*-7), 124.6 (*C*-9), 123.3 (*C*-3), 122.3 (*C*-6), 118.3 (*C*-2), 55.2 (*C*-10), 40.6 (*C*-15), 38.5 (*C*-12), 35.2 (*C*-14), 35.1 (*C*-11), 28.1 (*C*-13), 26.3 (*C*-16), 9.2 (*C*-17); **LR-ESI-MS:** $C_{17}H_{20}N_5$ $[M+H]^+$ *m/z* found 294.4, cald 294.2; **HR-ESI-MS:** $C_{17}H_{20}N_5$ $[M+H]^+$ *m/z* found 294.1744, cald 294.1719.

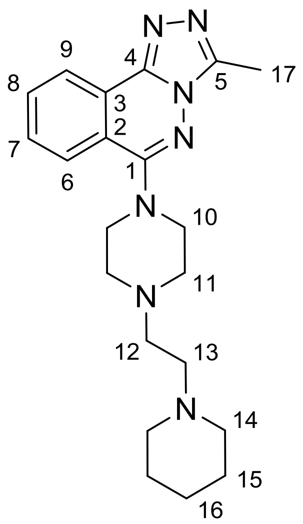

3-methyl-6-(4-(2-(piperidin-1-yl)ethyl)piperazin-1-yl)-[1,2,4]triazolo[3,4-a]phthalazine (62)

5 (200 mg, 0.915 mmol, 1 eq) and 1-(2-(piperidin-1-yl)ethyl)piperazine (361 mg, 1.829 mmol, 2 eq) were reacted according to general procedure **A**. The crude material was purified by Isolera Biotage LPLC (DCM/MeOH 90:10 then DCM/MeOH/NH_3 9:1:0.5) to give **62** (180 mg, 0.475 mmol 51.9%) as a white solid.

Mpt: 228.6-230.6 °C; **v_{max} (cm^{-1})** 3254, 2932, 2643, 2550, 1654, 1477, 989, 792; **^{1}H-NMR (400MHz, CDCl$_3$): δ_H** 8.60 (d, 1H, *J*= 7.7 Hz, *H*-9), 8.00 (d, 1H, *J*= 8.2 Hz, *H*-6), 7.84 (t, 1H, *J*= 7.3 Hz, *H*-8), 7.72 (m, 1H, *H*-7), 3.42 (br-s, 4H, *H*-10), 2.94 (br-s, 6H, *H*-11; *H*-12; *H*-13), 2.82 (br-s, 4H, *H*-14), 1.92 (br-s, 4H, *H*-15), 1.60 (br-s, 2H, *H*-16); **^{13}C-NMR (100 MHz, CDCl$_3$): δ_C** 157.5 (*C*-1), 147.6 (*C*-4), 142.5 (*C*-5), 132.9 (*C*-8), 129.9 (*C*-7), 126.3 (*C*-9), 125.0 (*C*-3), 123.8 (*C*-6), 119.9 (*C*-2), 54.5 (*C*-10), 52.9 (*C*-11), 51.1 (*C*-12; *C*-13), 26.9 (*C*-14), 23.8 (*C*-15), 22.9 (*C*-16); **LR-ESI-MS:** $C_{21}H_{30}N_7$ $[M+H]^+$ *m/z* found 380.3, cald 380.3; **HR-ESI-MS:** $C_{21}H_{30}N_7$ $[M+H]^+$ *m/z* found 380.2583, cald 380.2563.

***N*-((1-ethylpiperidin-2-yl)methyl)-3-methyl-[1,2,4]triazolo[3,4-a]phthalazin-6-amine (63)**

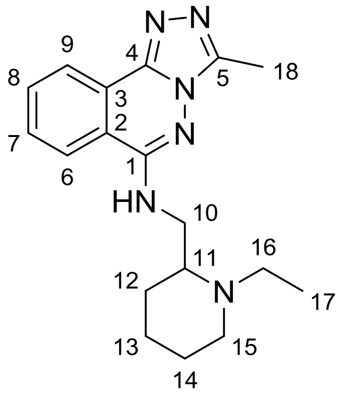

5 (200 mg, 0.915 mmol, 1 eq) and (1-ethylpiperidin-2-yl)methanamine (260 mg, 1.829 mmol, 2 eq) were reacted according to general procedure **A**. The crude material was purified by Isolera Biotage LPLC (DCM/MeOH 90:10 then DCM/MeOH/NH_3 9:1:0.5) to give **63** (99 mg, 0.307 mmol 33.6%) as a white solid.

Mpt: 169.0-171.0 °C; **v_{max} (cm^{-1})** 3250, 2946, 2660, 1510, 1339, 1212, 627, 573; **^{1}H-NMR (400MHz, DMSO-*d^6*): δ_H** 8.46 (d, 1H, *J*= 8.1 Hz, *H*-9), 8.37 (d, 1H, *J*= 7.7 Hz, *H*-6), 8.23 (m, 1H, N*H*), 7.92 (t, 1H, *J*= 7.5 Hz, *H*-8), 7.81 (m, 1H, *H*-7), 4.01 (m, 1H, *H*-11), 3.95-3.5 (m, 2H, *H*-10), 3.44 (m, 1H, *H*-15"), 3.07 (m, 1H, *H*-15'), 2.56 (s, 3H, *H*-18), 2.06 (m, 1H, *H*-12"), 2.0-1.7 (m, 4H, *H*-12'; *H*-14"; *H*-16), 1.49 (m, 1H, *H*-13"), 1.29 (m, 3H, *H*-13'; *H*-17); **^{13}C-NMR (100 MHz, DMSO-*d^6*): δ_C** 151.1 (*C*-1), 146.1 (*C*-4), 141.1 (*C*-5), 133.1 (*C*-8), 130.3 (*C*-7), 124.5 (*C*-9), 123.4 (*C*-3), 122.5 (*C*-6), 118.1 (*C*-2), 58.9 (*C*-11), 50.7 (*C*-11), 47.2 (*C*-15), 42.1 (*C*-16), 27.2 (*C*-12), 22.2 (*C*-14), 21.4 (*C*-13), 9.5 (*C*-18), 7.8 (*C*-17); **LR-ESI-MS:** $C_{18}H_{25}N_6$ $[M+H]^+$ *m/z* found 325.3, cald 325.2; **HR-ESI-MS:** $C_{18}H_{25}N_6$ $[M+H]^+$ *m/z* found 325.2160, cald 325.2141.

(*R*)-*N*-(1-benzylpiperidin-3-yl)-3-methyl-[1,2,4]triazolo[3,4-a]phthalazin-6-amine (64)

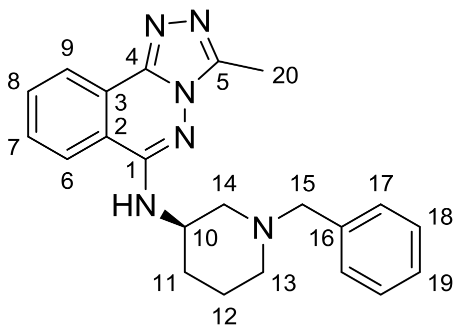

5 (400 mg, 1.829 mmol, 1 eq) and (R)-1-benzylpiperidin-3-amine (522 mg, 2.74 mmol, 1.5 eq) were reacted according to general procedure **A**. The crude material was purified by Isolera Biotage LPLC (DCM/MeOH 90:10 then DCM/MeOH/NH_3 9:1:0.5) to give **64** (153 mg, 0.411 mmol 22.5%) as a white solid.

Mpt: 122.5-124.5 °C; **ν_{max} (cm^{-1})** 3344, 2930, 2802, 1528, 1512, 1158, 733, 658; **^{1}H-NMR (400MHz, DMSO-*d*^{6}): δ_H** 8.34 (m, 2H, *H*-6; *H*-9), 7.89 (m, 1H, *H*-8), 7.78 (td, 1H, *J*= 7.7, 1.3 Hz, *H*-7), 7.31 (m, 4H, *H*-17; *H*-18), 7.23 (m, 1H, *H*-19), 7.13 (d, 1H, *J*= 7.3 Hz, N*H*), 4.09 (m, 1H, *H*-10), 3.63 (d, 1H, *J*= 13.3 Hz, *H*-15”), 3.40 (d, 1H, *J*= 13.3 Hz, *H*-15’), 3.18 (d, 1H, *J*= 7.7 Hz, *H*-14”), 2.82 (d, 1H, *J*= 11 Hz, *H*-14’), 2.44 (s, 3H, *H*-20), 2.02 (m, 2H, *H*-13), 1.86 (m, 1H, *H*-12”), 1.74 (m, 1H, *H*-11”), 1.62 (m, 1H, *H*-11’), 1.5 (m, 1H, *H*-12’); **^{13}C-NMR (100 MHz, DMSO-*d*^{6}): δ_C** 150.4 (*C*-1), 145.9 (*C*-4), 141.0 (*C*-5), 138.5 (*C*-16), 132.8 (*C*-8), 130.0 (*C*-7), 128.7 (*C*-17), 128.1 (*C*-18), 126.8 (*C*-19), 124.4 (*C*-9), 123.3 (*C*-3), 122.4 (*C*-6), 118.1 (*C*-2), 62.1 (*C*-15), 56.9 (*C*-14), 53.6 (*C*-13), 48.3 (*C*-10), 29.5 (*C*-11), 24.1 (*C*-12), 9.2 (*C*-20); **LR-ESI-MS:** $C_{22}H_{24}N_6$ $[M+H]^+$ *m/z* found 373.3, cald 373.2; **HR-ESI-MS:** $C_{22}H_{24}N_6$ $[M+H]^+$ *m/z* found 373.2173, cald 373.2141.

benzyl (*R*)-3-((3-methyl-[1,2,4]triazolo[3,4-a]phthalazin-6-yl)amino)piperidine-1-carboxylate (65)

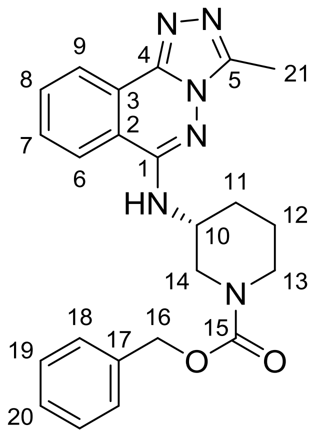

5 (300 mg, 1.372 mmol, 1 eq) and benzyl (*R*)-3-aminopiperidine-1-carboxylate (482 mg, 2.058 mmol, 1.5 eq) were reacted according to general procedure **A**. The crude material was purified by Isolera Biotage LPLC (DCM/MeOH 90:10 then DCM/MeOH/NH_3 9:1:0.5) to give **65** (93 mg, 0.223 mmol 16.3%) as a white solid.

Mpt: 172.2-174.2 °C; **ν_{max} (cm^{-1})** 3277, 2925, 2851, 1678, 1509, 1258, 1231, 696; **^{1}H-NMR (VT) (400MHz, 343 K, DMSO-*d*^{6}): δ_H** 8.38 (m, 2H, *H*-6; *H*-9), 7.91 (td, 1H, *J*= 7.6, 1.1 Hz, *H*-8), 7.79 (ddd, 1H, *J*= 8.3, 7.2, 1.4 Hz, *H*-7), 7.27 (s, 5H, *H*-18; *H*-19, *H*-20), 6.99 (d, 1H, *J*= 6.3 Hz, N*H*), 5.07 (s, 2H, *H*-16), 4.34 (m, 1H, *H*-14”), 4.02 (m, 1H, *H*-10), 3.85 (m, 1H, *H*-14’), 3.08 (m, 2H, *H*-13), 3.03 (s, 3H, *H*-21), 2.15 (m, 1H, *H*-11”), 1.82 (m, 2H, *H*-11’; *H*-12”), 1.55 (m, 1H, *H*-12’); **^{13}C-NMR (100 MHz, DMSO-*d*^{6}): δ_C** 154.5 (*C*-15), 150.5 (*C*-1), 146.0 (*C*-4), 141.0 (*C*-5), 136.9 (*C*-17), 132.9 (*C*-8), 130.0 (*C*-7), 128.2 (*C*-18), 127.7 (*C*-19), 127.1 (*C*-20), 124.6 (*C*-9), 123.4 (*C*-3), 122.4 (*C*-6), 118.1 (*C*-2), 66.2 (*C*-16), 47.8 (*C*-14), 43.8 (*C*-10), 29.3 (*C*-13), 26.3 (*C*-11; *C*-12), 9.1 (*C*-21); **LR-ESI-MS:** $C_{23}H_{25}N_6O_2$ $[M+H]^+$ *m/z* found 417.2, cald 417.2; **HR-ESI-MS:** $C_{23}H_{25}N_6O_2$ $[M+H]^+$ *m/z* found 417.2064, cald 417.2039.

(1*S*, 2*S*)-*N*$_1$,*N*$_1$-dimethyl-*N*$_2$-(3-methyl-[1,2,4]triazolo[3,4-a]phthalazin-6-yl)cyclohexane-1,2-diamine (66)

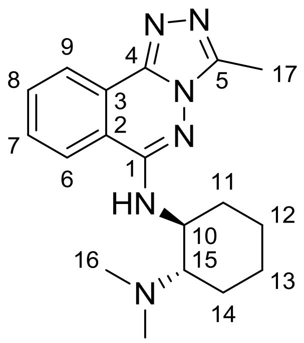

5 (300 mg, 1.372 mmol, 1 eq) and (1*S*,2*S*)-*N*$_1$,*N*$_1$-dimethylcyclohexane-1,2-diamine (390 mg, 2.74 mmol, 2 eq) were reacted according to general procedure **A**. The crude material was purified by Isolera Biotage LPLC (DCM/MeOH 90:10 then DCM/MeOH/NH$_3$ 9:1:0.5) to give **66** (245 mg, 0.755 mmol 55%) as a white solid.

Mpt: 183.0-185.0 °C; **ν$_{max}$ (cm^{-1})** 3306, 2993, 1592, 1498, 791; **^{1}H-NMR (400MHz, DMSO-*d^6*): δ$_H$** 8.37 (dd, 1H, *J*= 7.9, 1.0 Hz, *H*-9), 8.29 (d, 1H, *J*= 8.1 Hz, *H*-6), 7.94 (m, 1H, *H*-8), 7.81 (m, 1H, *H*-7), 7.03 (d, 1H, *J*= 6.5 Hz, N*H*), 3.92 (m, 1H, *H*-10), 2.65 (m, 1H, *H*-15), 2.55 (s, 3H, *H*-17), 2.32 (m, 1H, *H*-11''), 2.20 (s, 6H, *H*-16), 1.85 (m, 1H, *H*-11'), 1.81 (m, 1H, *H*-14''), 1.70 (m, 1H, *H*-14'), 1.28 (m, 4H, *H*-12; *H*-13); **^{13}C-NMR (100 MHz, DMSO-*d^6*): δ$_C$** 150.8 (*C*-1), 145.9 (*C*-4), 141.0 (*C*-5), 132.7 (*C*-8), 130.2 (*C*-7), 124.1 (*C*-9), 123.4 (*C*-3), 122.5 (*C*-6), 118.4 (*C*-2), 65.5 (*C*-15), 51.7 (*C*-10), 40.0 (*C*-16), 31.8 (*C*-11), 25.0 (*C*-12), 24.7 (*C*-14), 22.2 (*C*-13), 9.3 (*C*-17); **LR-ESI-MS:** $C_{18}H_{25}N_6$ [M+H]$^+$ *m/z* found 325.2, cald 325.2; **HR-ESI-MS:** $C_{18}H_{25}N_6$ [M+H]$^+$ *m/z* found 325.2160, cald 325.2141.

(1*R, 2*R**)-*N*$_1$,*N*$_1$-dimethyl-*N*$_2$-(3-methyl-[1,2,4]triazolo[3,4-a]phthalazin-6-yl)cyclopentane-1,2-diamine (67)**

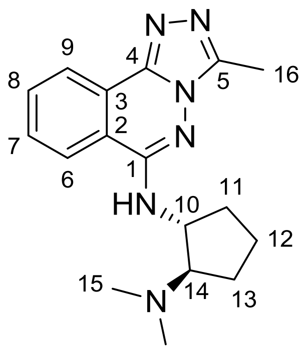

5 (300 mg, 1.372 mmol, 1 eq) and *rac*-(1*R*,2*R*)-*N*$_1$,*N*$_1$-dimethylcyclopentane-1,2-diamine (264 mg, 2.058 mmol, 1.5 eq) were reacted according to general procedure **A**. The crude material was purified by Isolera Biotage LPLC (DCM/MeOH 90:10 then DCM/MeOH/NH$_3$ 9:1:0.5) to give **67** (120 mg, 0.387 mmol 28%) as a white solid.

Mpt: 178.3-180.3 °C; **ν$_{max}$ (cm^{-1})** 3231, 2948, 1593, 1392, 1174, 769, 589; **^{1}H-NMR (400MHz, DMSO-*d^6*): δ$_H$** 8.43 (d, 1H, *J*= 8.1 Hz, *H*-9), 8.36 (dd, 1H, *J*= 7.9, 0.9 Hz, *H*-6), 7.91 (m, 1H, *H*-8), 7.80 (td, 1H, *J*= 7.8, 1.3 Hz, *H*-7), 7.35 (d, 1H, *J*= 7.6 Hz, N*H*), 4.35 (m, 1H, *H*-10), 2.91 (q, 1H, *J*= 7.1 Hz, *H*-14), 2.56 (s, 3H, *H*-16), 2.19 (s, 6H, *H*-15), 2.13 (m, 1H, *H*-11''), 1.81 (m, 1H, *H*-13''), 1.60 (m, 4H, *H*-11'; *H*-12; *H*-13'); **^{13}C-NMR (100 MHz, DMSO-*d^6*): δ$_C$** 150.6 (*C*-1), 146.0 (*C*-4), 141.0 (*C*-5), 132.7 (*C*-8), 130.0 (*C*-7), 124.4 (*C*-9), 123.3 (*C*-3), 122.4 (*C*-6), 118.3 (*C*-2), 71.0 (*C*-14), 54.5 (*C*-10), 42.8 (*C*-15), 31.6 (*C*-11), 27.2 (*C*-13), 22.0 (*C*-12), 9.3 (*C*-16); **LR-ESI-MS:** $C_{17}H_{23}N_6$ [M+H]$^+$ *m/z* found 311.2, cald 311.2; **HR-ESI-MS:** $C_{17}H_{23}N_6$ [M+H]$^+$ *m/z* found 311.2006, cald 311.1984.

_N,N_-dimethyl-1-(3-methyl-[1,2,4]triazolo[3,4-a]phthalazin-6-yl)pyrrolidin-3-amine (68)

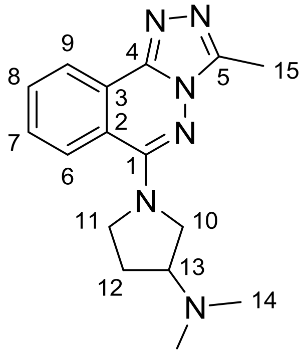

5 (200 mg, 0.915 mmol, 1 eq) and _N,N_-dimethylpyrrolidin-3-amine (209 mg, 1.829 mmol, 2 eq) were reacted according to general procedure **A**. The crude material was purified by Isolera Biotage LPLC (DCM/MeOH 90:10 then DCM/MeOH/NH_3 9:1:0.5) to give **68** (246 mg, 0.831 mmol 91%) as a white solid.

Mpt: 141.1-143.1 °C; **ν_{max} (cm^{-1})** 3380, 2864, 2785, 1681, 1493, 1420, 799, 704; **^{1}H-NMR (400MHz, $CDCl_3$): δ_H** 8.55 (dd, 1H, _J_= 7.9, 1.0 Hz, _H_-9), 8.09 (d, 1H, _J_= 8.3 Hz, _H_-6), 7.77 (m, 1H, _H_-8), 7.65 (ddd, 1H, _J_= 8.4, 7.2, 1.3 Hz, _H_-7), 3.92 (m, 1H, _H_-13), 3.84-3.69 (m, 3H, _H_-10; _H_-11”), 2.86 (m, 1H, _H_-11’), 2.66 (s, 3H, _H_-15), 2.35 (s, 6H, _H_-14), 2.22 (m, 1H, _H_-12”), 1.97 (m, 1H, _H_-12’); **^{13}C-NMR (100 MHz, $CDCl_3$): δ_C** 154.3 (_C_-1), 147.1 (_C_-4), 142.2 (_C_-5), 132.1 (_C_-8), 129.3 (_C_-7), 126.5 (_C_-9), 125.0 (_C_-3), 123.5 (_C_-6), 120.1 (_C_-2), 65.4 (_C_-13), 55.8 (_C_-10), 50.3 (_C_-11), 44.3 (_C_-14), 30.2 (_C_-12), 9.7 (_C_-15); **LR-ESI-MS:** $C_{16}H_{21}N_6$ [M+H]$^+$ _m/z_ found 297.2, cald 297.2; **HR-ESI-MS:** $C_{16}H_{21}N_6$ [M+H]$^+$ _m/z_ found 297.1850, cald 297.1828.

3-methyl-_N_-(2-(4-methylpiperazin-1-yl)ethyl)-[1,2,4]triazolo[3,4-a]phthalazin-6-amine (69)

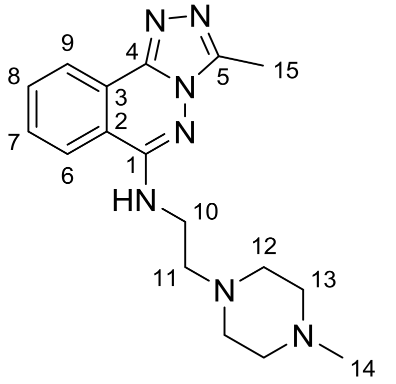

5 (200 mg, 0.915 mmol, 1 eq) and 2-(4-methylpiperazin-1-yl)ethan-1-amine (262 mg, 1.829 mmol, 2 eq) were reacted according to general procedure **A**. The crude material was purified by Isolera Biotage LPLC (DCM/MeOH 90:10 then DCM/MeOH/NH_3 9:1:0.5) to give **69** (189 mg, 0.582 mmol 63.6%) as an off white solid.

Mpt: 120.5-122.5 °C; **ν_{max} (cm^{-1})** 3223, 2938, 2802, 1566, 1390, 1269, 1145, 1006, 770, 698; **^{1}H-NMR (400MHz, $CDCl_3$): δ_H** 8.55 (d, 1H, _J_= 7.9 Hz, _H_-9), 7.78 (m, 2H, _H_-8; _H_-6), 7.68 (m, 1H, _H_-7), 6.19 (s, 1H, N_H_), 3.56 (m, 2H, _H_-10), 2.77 (t, 2H, _J_= 6 Hz, _H_-11), 2.66 (s, 3H, _H_-15), 2.61 (m, 4H, _H_-12), 2.50 (m, 4H, _H_-13), 2.31 (s, 3H, _H_-14); **^{13}C-NMR (100 MHz, $CDCl_3$): δ_C** 151.0 (_C_-1), 147.1 (_C_-4), 141.9 (_C_-5), 132.3 (_C_-8), 129.9 (_C_-7), 124.1 (_C_-9), 123.6 (_C_-3), 122.3 (_C_-6), 118.4 (_C_-2), 55.5 (_C_-10), 55.2 (_C_-12), 52.6 (_C_-13), 45.9 (_C_-11), 37.8 (_C_-14), 9.7 (_C_-15); **LR-ESI-MS:** $C_{17}H_{24}N_7$ [M+H]$^+$ _m/z_ found 326.2, cald 326.2; **HR-ESI-MS:** $C_{17}H_{24}N_7$ [M+H]$^+$ _m/z_ found 326.2114, cald 326.2093.

3-methyl-_N_-(1-(4-methylpiperidin-1-yl)propan-2-yl)-[1,2,4]triazolo[3,4-a]phthalazin-6-amine (70)

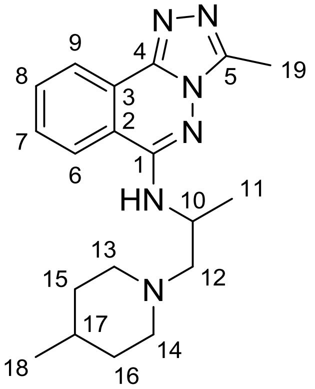

5 (200 mg, 0.915 mmol, 1 eq) and 2-(4-methylpiperazin-1-yl)ethan-1-amine (286 mg, 1.829 mmol, 2 eq) were reacted according to general procedure **A**. The crude material was purified by Isolera Biotage LPLC (DCM/MeOH 90:10 then DCM/MeOH/NH_3 9:1:0.5) to give **70** (40 mg, 0.117 mmol 12.8%) as an off white solid.

Mpt: 128.6-130.6 °C; **ν_{max} (cm^{-1})** 3249, 2919, 1507, 1083, 699; **^{1}H-NMR (400MHz, DMSO-_d^6_): δ_H** 8.36 (m, 2H, _H_-6; _H_-9), 7.9 (m, 1H, _H_-8), 7.8 (m, 1H, _H_-7), 7.16 (d, 1H, _J_= 6.7 Hz, N_H_), 4.28 (m, 1H, _H_-10), 3.00 (d, 1H, _J_= 9.8 Hz, _H_-12”), 2.84 (m, 1H, _H_-12’), 2.59 (m, 1H, _H_-13”), 2.54 (s, 3H, _H_-

19), 2.30 (m, 1H, 13'), 1.97 (m, 2H, *H*-14), 1.52 (t, 2H, *J*= 15 Hz, *H*-15), 1.30 (d, 3H, *H*-11), 1.27 (m, 1H, *H*-17), 1.06 (m, 2H, *H*-16), 0.82 (d, 3H, *J*= 6.4 Hz, *H*-18); **^{13}C-NMR (100 MHz, DMSO-*d*^{6}): δ$_C$** 150.7 (*C*-1), 145.9 (*C*-4), 141.0 (*C*-5), 132.7 (*C*-8), 130.0 (*C*-7), 124.3 (*C*-9), 123.4 (*C*-3), 122.4 (*C*-6), 118.3 (*C*-2), 63.1 (*C*-12), 54.2 (*C*-13), 53.5 (*C*-14), 44.4 (*C*-10), 34.0 (*C*-15), 30.3 (*C*-16), 26.3 (*C*-17), 21.8 (*C*-18), 18.5 (*C*-11), 9.2 (*C*-19); **LR-ESI-MS:** $C_{19}H_{27}N_6$ [M+H]$^+$ *m/z* found 339.2, cald 339.2; **HR-ESI-MS:** $C_{19}H_{27}N_6$ [M+H]$^+$ *m/z* found 339.2320, cald 339.2297.

3-methyl-*N*-(1-(pyridin-2-yl)ethyl)-[1,2,4]triazolo[3,4-a]phthalazin-6-amine (71)

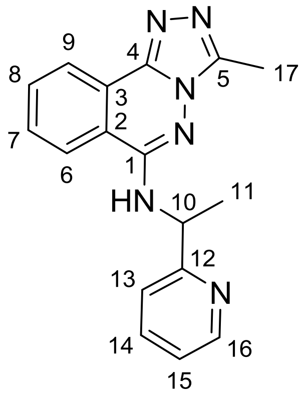

5 (300 mg, 1.372 mmol, 1 eq) and 1-(pyridin-2-yl)ethan-1-amine (335 mg, 2.74 mmol, 2 eq) were reacted according to general procedure **A**. The crude material was purified by Isolera Biotage LPLC (DCM/MeOH 90:10 then DCM/MeOH/NH_3 9:1:0.5) to give **71** (172 mg, 0.567 mmol 41.4%) as an off white solid.

Mpt: 143.5-145.5 °C; $ν_{max}$ (cm^{-1}) 3266, 1591, 1475, 1376, 1164, 698; **^{1}H-NMR (400MHz, CDCl$_3$): δ$_H$** 8.60 (d, 1H, *J*= 4.6 Hz, *H*-16), 8.53 (d, 1H, *J*= 7.8 Hz, *H*-9), 7.94 (d, 1H, *J*= 8.2 Hz, *H*-6), 7.74 (m, 2H, *H*-14; *H*-8), 7.64 (m, 1H, *H*-7), 7.41 (d, 1H, *J*= 7.8 Hz, *H*-13), 7.25 (m, 1H, *H*-15), 7.15 (d, 1H, *J*= 6 Hz, N*H*), 5.29 (quin, 1H, *J*= 6.5 Hz, *H*-10), 2.65 (s, 3H, *H*-17), 1.69 (d, 3H, *J*= 6.6 Hz, *H*-11); **^{13}C-NMR (100 MHz, CDCl$_3$): δ$_C$** 161.3 (*C*-12), 150.0 (*C*-1), 148.6 (*C*-16), 147.0 (*C*-4), 141.9 (*C*-5), 137.0 (*C*-14), 132.4 (*C*-8), 129.9 (*C*-7), 124.1 (*C*-9), 123.6 (*C*-3), 122.7 (*C*-13), 122.4 (*C*-6), 121.6 (*C*-15), 118.5 (*C*-2), 51.5 (*C*-10), 21.8 (*C*-11), 9.7 (*C*-17); **LR-ESI-MS:** $C_{17}H_{17}N_6$ [M+H]$^+$ *m/z* found 305.2, cald 305.2; **HR-ESI-MS:** $C_{17}H_{17}N_6$ [M+H]$^+$ *m/z* found 305.1556, cald 305.1515.

3-methyl-*N*-(1-(pyrazin-2-yl)ethyl)-[1,2,4]triazolo[3,4-a]phthalazin-6-amine (72)

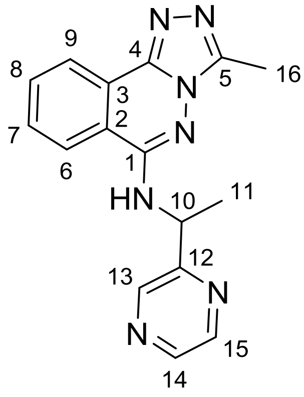

5 (300 mg, 1.372 mmol, 1 eq) and 1-(pyrazin-2-yl)ethan-1-amine (338 mg, 2.74 mmol, 2 eq) were reacted according to general procedure **A**. The crude material was purified by Isolera Biotage LPLC (DCM/MeOH 90:10 then DCM/MeOH/NH_3 9:1:0.5) to give **72** (66 mg, 0.215 mmol 15.6%) as an off white solid.

Mpt: 172.9-174.9 °C; **$ν_{max}$ (cm^{-1})** 3201, 3057, 1513, 1407, 1141, 701, 629, 604; **^{1}H-NMR (400MHz, CDCl$_3$): δ$_H$** 8.78 (d, 1H, *J*= 1.3 Hz, *H*-13), 8.59 (d, 1H, *J*= 7.3 Hz, *H*-9), 8.55 (m, 1H, *H*-15), 8.52 (m, 1H, *H*-15), 7.97 (d, 1H, *H*-6), 7.82 (m, 1H, *H*-8), 7.73 (m, 1H, *H*-7), 6.59 (d, 1H, N*H*), 5.40 (quin, 1H, *J*= 6.7 Hz, *H*-10), 2.64 (s, 3H, *H*-16), 1.75 (d, 3H, *J*= 6.7 Hz, *H*-11); **^{13}C-NMR (100 MHz, CDCl$_3$):** δ$_C$ 157.2 (*C*-12), 150.0 (*C*-1), 147.1 (*C*-4), 143.9 (*C*-15), 143.8 (*C*-14), 143.5 (*C*-12), 141.7 (*C*-5), 132.8 (*C*-8), 130.3 (*C*-7), 124.0 (*C*-9; *C*-3), 122.6 (*C*-6), 118.3 (*C*-2), 49.9 (*C*-10), 21.6 (*C*-11), 9.7 (*C*-16); **LR-ESI-MS:** $C_{16}H_{16}N_7$ [M+H]$^+$ *m/z* found 306.1, cald 306.1; **HR-ESI-MS:** $C_{16}H_{16}N_7$ [M+H]$^+$ *m/z* found 306.1509, cald 306.1467.

3-methyl-6-(octahydro-2H-pyrido[1,2-a]pyrazin-2-yl)-[1,2,4]triazolo[3,4-a]phthalazine (73)

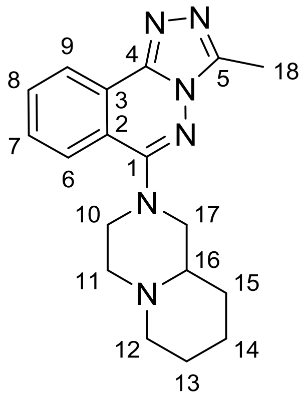

5 (200 mg, 0.915 mmol, 1 eq) and octahydro-2H-pyrido[1,2-a]pyrazine (257 mg, 1.829 mmol, 2 eq) were reacted according to general procedure **A**. The crude material was purified by Isolera Biotage LPLC (DCM/MeOH 90:10 then DCM/MeOH/NH_3 9:1:0.5) to give **73** (228 mg, 0.709 mmol 78%) as an off white solid.

Mpt: 128.7-130.7 °C; **ν_{max} (cm^{-1})** 2929, 2807, 1510, 1345, 1259, 1124, 748; **^{1}H-NMR (400MHz, CDCl$_3$): δ_H** 8.60 (dd, 1H, *J*= 7.9, 0.6 Hz, *H*-9), 8.01 (d, 1H, *J*= 8.2 Hz, *H*-6), 7.83 (m, 1H, *H*-8), 7.71 (m, 1H, *H*-7), 3.70 (dd, 1H, *J*= 12.6, 2 Hz, *H*-17”), 3.57 (d, 1H, *J*= 12.6 Hz, *H*-17’), 3.37 (m, 1H, *H*-16), 2.98 (m, 3H, *H*-11; *H*-10”), 2.74 (s, 3H, *H*-18), 2.64 (m, 1H, *H*-10’), 2.30 (m, 2H, *H*-12), 1.69 (m, 6H, *H*-13; *H*-14, *H*-15); **^{13}C-NMR (100 MHz, CDCl$_3$): δ_C** 157.2 (*C*-1), 147.7 (*C*-4), 142.4 (*C*-5), 132.8 (*C*-8), 129.8 (*C*-7), 126.3 (*C*-9), 125.1 (*C*-3), 123.8 (*C*-6), 120.0 (*C*-2), 61.1 (*C*-16), 56.6 (*C*-17), 55.6 (*C*-11), 54.4 (*C*-10), 50.8 (*C*-12), 29.2 (*C*-15), 25.2 (*C*-13), 23.7 (*C*-14), 9.8 (*C*-18); **LR-ESI-MS:** $C_{18}H_{23}N_6$ [M+H]$^+$ *m/z* found 323.2, cald 323.2; **HR-ESI-MS:** $C_{18}H_{23}N_6$ [M+H]$^+$ *m/z* found 323.2007, cald 323.1984.

***N*-((1,4-dimethylpiperazin-2-yl)methyl)-3-methyl-[1,2,4]triazolo[3,4-a]phthalazin-6-amine (74)**

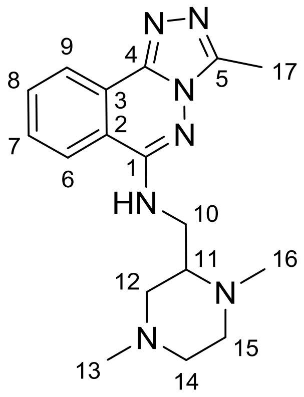

5 (200 mg, 0.915 mmol, 1 eq) and (1,4-dimethylpiperazin-2-yl)methanamine (262 mg, 1.829 mmol, 2 eq) were reacted according to general procedure **A**. The crude material was purified by Isolera Biotage LPLC (DCM/MeOH 90:10 then DCM/MeOH/NH_3 9:1:0.5) to give **74** (89 mg, 0.273 mmol 29.9%) as an off white solid.

Mpt: 251.6-253.6 °C; ν_{max} (cm^{-1}) 3226, 3064, 2794, 1512, 1265, 1166, 697; **^{1}H-NMR (400MHz, CDCl$_3$):** δ_H 8.56 (m, 1H, *H*-9), 7.80 (m, 2H, *H*-6; *H*-8), 7.69 (m, 1H, *H*-7), 6.43 (m, 1H, N*H*), 3.66 (m, 1H, *H*-10”), 3.58 (m, 1H, *H*-10’), 2.94 (dt, 1H, *J*= 11.6, 3 Hz, *H*-15”), 2.81 (m, 2H, *H*-12”; *H*-14”), 2.69 (m, 1H, *H*-15’), 2.68 (s, 3H, *H*-17), 2.53 (td, 1H, *J*= 10.9, 2.8 Hz, *H*-11), 2.41 (s, 3H, *H*-16), 2.36 (m, 2H, *H*-12’; *H*-14’), 2.29 (s, 3H, *H*-13); **^{13}C-NMR (100 MHz, CDCl$_3$): δ_C** 151.1 (*C*-1), 147.2 (*C*-4), 142.0 (*C*-5), 132.4 (*C*-8), 129.9 (*C*-7), 124.1 (*C*-9), 123.7 (*C*-3), 122.4 (*C*-6), 118.5 (*C*-2), 59.2 (*C*-11), 58.4 (*C*-12), 54.7 (*C*-14), 45.9 (*C*-13; *C*-16), 42.1 (*C*-15), 41.5 (*C*-10), 9.8 (*C*-17); **LR-ESI-MS:** $C_{17}H_{24}N_7$ [M+H]$^+$ *m/z* found 326.2, cald 326.2; **HR-ESI-MS:** $C_{17}H_{24}N_7$ [M+H]$^+$ *m/z* found 326.2121, cald 326.2093.

***N*,*N*-dimethyl-1-(3-methyl-[1,2,4]triazolo[3,4-a]phthalazin-6-yl)piperidin-3-amine (75)**

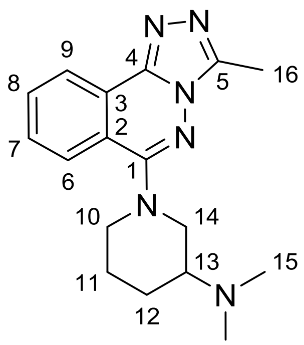

5 (200 mg, 0.915 mmol, 1 eq) and *N*,*N*-dimethylpiperidin-3-amine (235 mg, 1.829 mmol, 2 eq) were reacted according to general procedure **A**. The crude material was purified by Isolera Biotage LPLC (DCM/MeOH 90:10 then DCM/MeOH/NH_3 9:1:0.5) to give **75** (227 mg, 0.730 mmol 80%) as an off white solid.

Mpt: 139.7-141.7 °C; **ν_{max} (cm^{-1})** 2932, 2825, 1383, 1252, 703; **^{1}H-NMR (400MHz, CDCl$_3$): δ_H** 8.58 (dd, 1H, *J*= 7.9, 0.7 Hz, *H*-9), 8.00 (d, 1H, *J*= 8.1

Hz, *H*-6), 7.82 (m, 1H, *H*-8), 7.71 (m, 1H, *H*-7), 3.97 (m, 1H, *H*-14”), 3.7 (m, 1H, *H*-14’), 2.92 (m, 3H, *H*-10; *H*-13), 2.72 (s, 3H, *H*-16), 2.46 (s, 6H, *H*-15), 2.19 (m, 1H, *H*-12”), 1.98 (m, 1H, *H*-12’), 1.86 (m, 1H, *H*-11”), 1.56 (dd, 1H, *J*= 11.2, 4.1 Hz, *H*-11’); **^{13}C-NMR (100 MHz, $CDCl_3$): δ_C** 157.8 (*C*-1), 147.6 (*C*-4), 142.5 (*C*-5), 132.8 (*C*-8), 129.9 (*C*-7), 126.4 (*C*-9), 125.0 (*C*-3), 123.7 (*C*-6), 120.3 (*C*-2), 61.1 (*C*-13), 53.8 (*C*-14), 52.1 (*C*-10), 41.9 (*C*-15), 27.0 (*C*-12), 24.4 (*C*-11), 9.8 (*C*-16); **LR-ESI-MS:** $C_{17}H_{23}N_6$ $[M+H]^+$ *m/z* found 311.3, cald 311.2; **HR-ESI-MS:** $C_{17}H_{23}N_6$ $[M+H]^+$ *m/z* found 311.2000, cald 311.1984.

(1*S*, 2*S*)-*N*$_1$,*N*$_1$-dimethyl-*N*$_2$-(3-methyl-[1,2,4]triazolo[3,4-a]phthalazin-6-yl)cyclopentane-1,2-diamine (76)

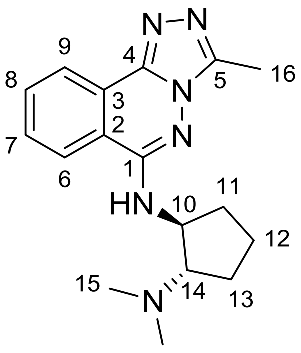

5 (200 mg, 0.915 mmol, 1 eq) and (1*S*,2*S*)-*N*$_1$,*N*$_1$-dimethylcyclopentane-1,2-diamine, 2HCl (276 mg, 1.372 mmol, 1.5 eq) were reacted according to general procedure **A** with added DIPEA (478 µL, 2.74 mmol, 3 eq). The crude material was purified by Isolera Biotage LPLC (DCM/MeOH 90:10 then DCM/MeOH/NH_3 9:1:0.5) to give **76** (48 mg, 0.155 mmol 16.9%) as an off white solid.

Mpt: 211.0-213.0 °C; **ν_{max} (cm^{-1})** 3238, 2954, 1561, 1551, 1264, 699; **^{1}H-NMR (400 MHz, DMSO-*d*^{6}): δ_H** 8.38 (m, 2H, *H*-9; *H*-6), 7.91 (t, 1H, *J*= 7.6 Hz, *H*-8), 7.80 (m, 1H, *H*-7), 7.37 (d, 1H, *J*= 7.6 Hz, N*H*), 4.34 (quin, 1H, *J*= 6.9 Hz, *H*-10), 2.92 (q, 1H, *J*= 7.3 Hz, *H*-14), 2.56 (s, 3H, *H*-16), 2.19 (s, 6H, *H*-15), 2.10 (m, 1H, *H*-11”), 1.83 (m, 1H, *H*-13”), 1.60 (m, 4H, *H*-11’; *H*-12; *H*-13’); **^{13}C-NMR (100 MHz, DMSO-*d*^{6}): δ_C** 151.0 (*C*-1), 146.5 (*C*-4), 141.4 (*C*-5), 133.2 (*C*-8), 130.6 (*C*-7), 124.7 (*C*-9), 123.4 (*C*-3), 122.9 (*C*-6), 118.7 (*C*-2), 71.3 (*C*-14), 54.7 (*C*-10), 43.0 (*C*-15), 31.9 (*C*-11), 27.5 (*C*-13), 22.3 (*C*-12), 9.6 (*C*-16); **LR-ESI-MS:** $C_{17}H_{23}N_6$ $[M+H]^+$ *m/z* found 311.3, cald 311.2; **HR-ESI-MS:** $C_{17}H_{23}N_6$ $[M+H]^+$ *m/z* found 311.1996, cald 311.1984.

***E/Z*-(2-nitroprop-1-en-1-yl)benzene (18)**

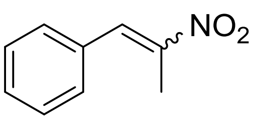

A solution of benzaldehyde (20 g, 188 mmol, 1 eq) and ammonium acetate (2.91 g, 37.7 mmol, 0.2 eq) in nitroethane (314 mL, 0.6 M) was s stirred at 110 °C for 48 h. After reaction completion the mixture was cooled to 4 °C and washed with cold CH before being filtered off and dried to give *E/Z*-**18** as a 1:1 mixture (19.266 g, 118 mmol, 62.7%) which was submitted to the following step without further purification.

methyl *E/Z*-(2-nitroprop-1-en-1-yl)benzoate (19)

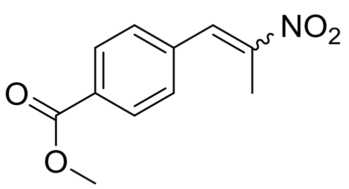

A solution of methyl 4-formylbenzoate (5.31 g, 32.3 mmol, 1 eq) and ammonium acetate (0.499 g, 6.47 mmol, 0.2 eq) in nitroethane (54 mL, 0.6 M) was stirred at 110 °C for 6 days. After reaction completion the mixture was cooled to 4 °C and washed with cold CH before being filtered off and dried to give *E/Z*-**19** (6.554 g, 29.6 mmol, 92%) which was submitted to the following step without further purification.

***E/Z*-1-fluoro-4-(2-nitroprop-1-en-1-yl)benzene (20)**

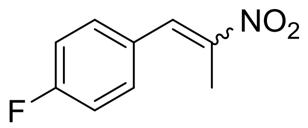

A solution of 4-fluorobenzaldehyde (20 g, 161 mmol, 1 eq) and ammonium acetate (2.484 g, 32.2 mmol, 0.2 eq) in nitroethane (269 mL, 0.6 M) was stirred at 110 °C for 24 h. After reaction completion the mixture was cooled to 4 °C and washed with cold CH before being filtered off and dried to give *E/Z*-**20** (14.95 g, 83 mmol, 51.2%) which was submitted to the following step without further purification.

***E/Z*-1-(2-nitroprop-1-en-1-yl)-4-(trifluoromethyl)benzene (21)**

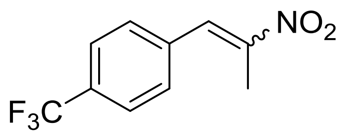

A solution of 4-(trifluoromethyl)benzaldehyde (8 g, 45.9 mmol, 1 eq) and ammonium acetate (0.708 g, 9.19 mmol, 0.2 eq) in nitroethane (77 mL, 0.6 M) was stirred at 110 °C for 4 days. After reaction completion the mixture was cooled to 4 °C and washed with cold CH before being filtered off and dried to give *E/Z*-**21** (10.62 g, 45.9 mmol, 100%) which was submitted to the following step without further purification.

***E/Z*-1-methoxy-4-(2-nitroprop-1-en-1-yl)benzene (22)**

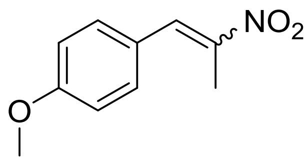

A solution of 4-methoxybenzaldehyde (10 g, 73.4 mmol, 1 eq) and ammonium acetate (1.132 g, 14.69 mmol, 0.2 eq) in nitroethane (122 mL, 0.6 M) was stirred at 110 °C for 3 days. After reaction completion the mixture was cooled to 4 °C and washed with cold CH before being filtered off and dried to give *E/Z*-**22** (14.19 g, 73.4 mmol, 100%) which was submitted to the following step without further purification.

***E/Z*-1-chloro-4-(2-nitroprop-1-en-1-yl)benzene (23)**

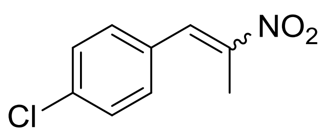

A solution of 4-chlorobenzaldehyde (10 g, 71.1 mmol, 1 eq) and ammonium acetate (1.097 g, 14.23 mmol, 0.2 eq) in nitroethane (119 mL, 0.6 M) was stirred at 110 °C for 4 days. After reaction completion the mixture was cooled to 4 °C and washed with cold CH before being filtered off and dried to give *E/Z*-**23** (14.06 g, 71.1 mmol, 100%) which was submitted to the following step without further purification.

***E/Z*- 1-methyl-4-(2-nitroprop-1-en-1-yl)benzene (24)**

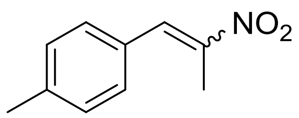

A solution of 4-methylbenzaldehyde (10 g, 83 mmol, 1 eq) and ammonium acetate (1.283 g, 16.65 mmol, 0.2 eq) in nitroethane (139 mL, 0.6 M) was stirred at 110 °C for 16 h. After reaction completion the mixture was cooled to 4 °C and washed with cold CH before being filtered off and dried to give *E/Z*-**24** (14.75 g, 83 mmol, 100%) which was submitted to the following step without further purification.

(1*S, 2*S**)-*N*,*N*-dimethyl-2-nitro-1-phenylpropan-1-amine (25)**

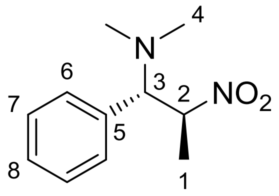

To a stirred solution of *E*/*Z*-**18** (1 g, 6.13 mmol,1 eq) in THF (15 mL, anhydrous) was added Dimethylamine 2M (in THF) (15 mL, 30.6 mmol, 5 eq) at room temperature and the solution was allowed to stir for 16 h. After reaction completion the solution was concentrated to a yellow crystalline crude material which was immediately submitted to a purification by Isolera Biotage LPLC (CH/EA 90:10 to CH/EA 2:8) to give **25** (1.0219 g, >33:1 dr, 70.5%) as a unstable white waxy solid.

ν_{max} (cm^{-1}) 2975, 2944, 2835, 2791, 1556, 1451, 1353, 873, 705, 614; **^{1}H-NMR (400 MHz, $CDCl_3$): δ_H** 7.39 (m, 3H, *H*-7; *H*-8), 7.11 (m, 1H, *H*-6), 5.23 (dq, 1H, *J*= 11.1, 6.6 Hz, *H*-2), 4.01 (d, 1H, *J*= 11 Hz, *H*-3), 2.15 (s, 6H, *H*-4), 1.34 (d, 1H, *J*= 6.6 Hz, *H*-1); **^{13}C-NMR (100 MHz, $CDCl_3$): δ_C** 131.0 (*C*-5), 129.2 (*C*-6), 128.3 (*C*-7), 128.2 (*C*-8), 83.7 (*C*-2), 72.3 (*C*-3), 40.9 (*C*-4), 17.2 (*C*-1); **LR-ESI-MS:** $C_{11}H_{17}N_2O_2$ $[M+H]^+$ *m/z* found 209.1, cald 209.1. **HR-ESI-MS:** $C_{11}H_{17}N_2O_2$ $[M+H]^+$ *m/z* found 209.0879, cald 209.1290.

methyl 4-((1*S, 2*S**)-1-(dimethylamino)-2-nitropropyl)benzoate (26)**

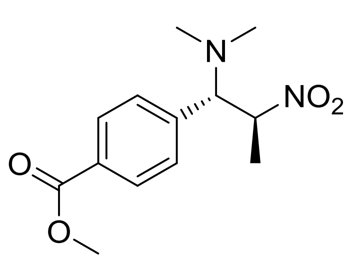

To a stirred solution of *E*/*Z*-**19** (1.133 g, 5.12 mmol,1 eq) in THF (10 mL, anhydrous) was added Dimethylamine 2M (in THF) (12.8 mL, 25.6 mmol, 5 eq) at room temperature and the solution was allowed to stir for 16 h. After reaction completion the solution was concentrated to give **26** (1.364 g, 5.12 mmol, 12:1 dr, 100%) as an unstable red crude material which was immediately submitted to the next step without further purification.

(1*S, 2*S**)-1-(4-fluorophenyl)-*N*,*N*-dimethyl-2-nitropropan-1-amine (27)**

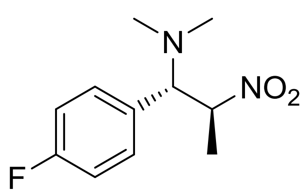

To a stirred solution of *E*/*Z*-**20** (5 g, 27.6 mmol,1 eq) in THF (20 mL, anhydrous) was added Dimethylamine 2M (in THF) (69 mL, 138 mmol, 5 eq) at room temperature and the solution was allowed to stir for 16 h. After reaction completion the solution was concentrated to give **27** (6.24 g, 27.6 mmol, 15:1 dr, 100%) as an unstable yellow crystalline crude material which was immediately submitted to the next step without further purification.

(1*S, 2*S**)-1-(4-methoxyphenyl)-*N*,*N*-dimethyl-2-nitropropan-1-amine (28)**

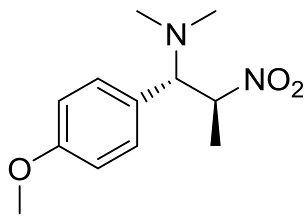

To a stirred solution of *E*/*Z*-**22** (14.19 g, 73.4 mmol,1 eq) in THF (30 mL, anhydrous) was added Dimethylamine 2M (in THF) (184 mL, 367 mmol, 5 eq) at room temperature and the solution was allowed to stir for 48 h. After reaction completion the solution was concentrated to give **28** (17.5 g, 73.4 mmol, 7.5:1 dr, 100%) as an unstable red solid which was immediately submitted to the next step without further purification.

(1*S, 2*S**)-*N,N*-dimethyl-2-nitro-1-(4-(trifluoromethyl)phenyl)propan-1-amine (29)**

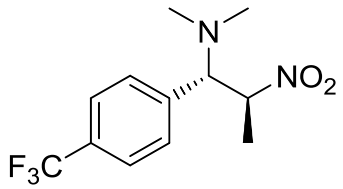

To a stirred solution of *E*/*Z*-**21** (10.62 g, 45.9 mmol,1 eq) in THF (30 mL, anhydrous) was added Dimethylamine 2M (in THF) (115 mL, 230 mmol, 5 eq) at room temperature and the solution was allowed to stir for 16 h. After reaction completion the solution was concentrated to give **29** (12.69 g, 45.9 mmol, 6.8:1 dr, 100%) as an unstable red solid which was immediately submitted to the next step without further purification.

(1*S, 2*S**)-*N,N*-dimethyl-2-nitro-1-(p-tolyl)propan-1-amine (30)**

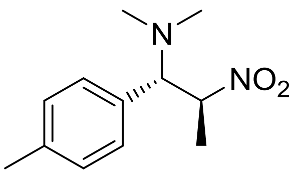

To a stirred solution of *E*/*Z*-**24** (14.75 g, 83 mmol,1 eq) in THF (30 mL, anhydrous) was added Dimethylamine 2M (in THF) (208 mL, 291 mmol, 5 eq) at room temperature and the solution was allowed to stir for 16 h. After reaction completion the solution was concentrated to give **30** (18.5 g, 83 mmol, 13:1 dr, 100%) as an unstable dark red solid which was immediately submitted to the next step without further purification.

(1*S, 2*S**)-1-(4-chlorophenyl)-*N,N*-dimethyl-2-nitropropan-1-amine (31)**

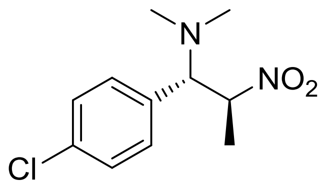

To a stirred solution of *E*/*Z*-**23** (14.06 g, 71.1 mmol,1 eq) in THF (30 mL, anhydrous) was added Dimethylamine 2M (in THF) (178 mL, 249 mmol, 5 eq) at room temperature and the solution was allowed to stir for 16 h. After reaction completion the solution was concentrated to give **31** (17.27 g, 71.1 mmol, 4.6:1 dr, 100%) as an unstable dark red solid which was immediately submitted to the next step without further purification.

(1*S, 2*S**)-*N*$_1$,*N*$_1$-dimethyl-1-phenylpropane-1,2-diamine (32)**

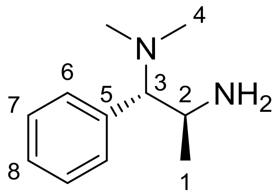

To a degassed stirred solution of **25** (4.2127 g, 20.23 mmol,1 eq) in MeOH (101 mL, 0.2 M, anhydrous) was added Pd/C (10%) (0.42 g, 0.195 eq) and atmosphere was exhanged with H_2 gas (x3). The reaction mixture was stirred under a H_2 atmsophere (double balloon, 1 bar) for 5 h. Upon reaction completion the mixture was filtered through celite, followed by filtration through a sintered frit to afford a crude oil after drying. The oily residue was purified by Isolera Biotage LPLC (DCM/MeOH 90:10 then DCM/MeOH/NH_3 9:1:0.5) to give **32** (0.4 g, 2.244 mmol, single diastereomer,11% over two steps) as a colourless oil.

ν_{max} (cm^{-1}) 2963, 2780, 1581, 1452, 1375, 753, 704; **^{1}H-NMR (400 MHz, $CDCl_3$): δ_H** 7.46 (m, 2H, *H*-7, *H*-8), 7.26 (m, 2H, *H*-6), 3.56 (m, 1H, *H*-2), 3.19 (d, 1H, *J*= 9.5 Hz, *H*-3), 2.28 (s, 6H, *H*-4), 1.03 (d, 3H, *J*= 6.2 Hz, *H*-1); **^{13}C-NMR (100 MHz DMSO-*d*^{6}): δ_C** 135.3 (*C*-5), 129.4 (*C*-6), 127.6 (*C*-7), 126.8 (*C*-8), 79.2 (*C*-3), 45.2 (*C*-2), 40.9 (*C*-4), 20.4 (*C*-1); **LR-ESI-MS:** $C_{11}H_{19}N_2$ [M+H]$^+$ *m/z* found 179.2, cald 179.2; **HR-ESI-MS:** $C_{11}H_{19}N_2$ [M+H]$^+$ *m/z* found 179.1549, cald 179.1548.

(1*S, 2*S**)-1-(4-fluorophenyl)-*N*$_1$,*N*$_1$-dimethylpropane-1,2-diamine (33)**

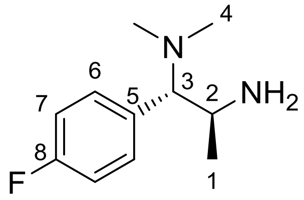

To a degassed stirred solution of **27** (6.24 g, 27.6 mmol,1 eq) in MeOH (68.9 mL, 0.4 M, anhydrous) was added Pd/C (10%) (0.646 g, 0.22 eq) and atmosphere was exhanged with H_2 gas (x3). The reaction mixture was stirred under a H_2 atmsophere (double balloon, 1 bar) for 16 h. Upon reaction completion the mixture was filtered through celite, followed by filtration through a sintered frit to afford a yellow crude oil after drying. The oily residue was purified by Isolera Biotage LPLC (DCM/MeOH 90:10 then DCM/MeOH/NH_3 9:1:0.5) to give **33** (0.569 g, 2.90 mmol, single diastereomer,10.5% over two steps) as a colourless oil.

ν_{max} (cm^{-1}) 2827, 1599, 1506, 1222, 846; **^{19}F-{H}-NMR (376 MHz, CDCl$_3$): δ_F** -115.77; **^{1}H-{F}-NMR (400 MHz, CDCl$_3$): δ_H** 7.08 (d, 2H, *J*= 8.7 Hz, *H*-7), 7.02 (d, 2H, *J*= 8.7 Hz, *H*-6), 3.36 (dq, 1H, *J*= 9.8, 6.2 Hz, *H*-2), 3.03 (d, 1H, *J*= 9.2 Hz, *H*-3), 2.11 (s, 6H, *H*-4), 0.86 (d, 3H, *J*= 6.2 Hz, *H*-1); **^{13}C-NMR (100 MHz CDCl$_3$): δ_C** 162.0 (d, *J*= 245 Hz, *C*-8), 131.0 (d, *J*= 3.7 Hz, *C*-5), 130.8 (d, *J*= 8.1 Hz, *C*-6), 114.6 (d, *J*= 20.5 Hz, *C*-7), 76.2 (*C*-3), 46.0 (*C*-2), 41.1 (*C*-4), 20.4 (*C*-1); **LR-ESI-MS:** $C_{11}H_{18}FN_2$ [M+H]$^+$ *m/z* found 197.2, cald 197.2; **HR-ESI-MS:** $C_{11}H_{17}FN_2Na$ [M+Na]$^+$ *m/z* found 219.1363, cald 219.1273.

(1*S, 2*S**)-*N*$_1$,*N*$_1$-dimethyl-1-(p-tolyl)propane-1,2-diamine (34)**

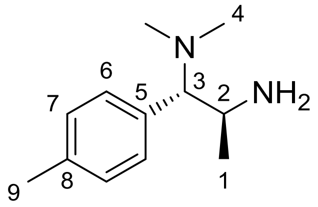

To a degassed stirred solution of **30** (1 g, 4.50 mmol,1 eq) in MeOH (30 mL, 0.15 M, anhydrous) was added Pd/C (10%) (0.144 g, 0.3 eq) and atmosphere was exhanged with H_2 gas (x3). The reaction mixture was stirred under a H_2 atmsophere (double balloon, 1 bar) for 16 h. Upon reaction completion the mixture was filtered through celite, followed by filtration through a sintered frit to afford a yellow crude oil after drying. The oily residue was purified by Isolera Biotage LPLC (DCM/MeOH 90:10 then DCM/MeOH/NH_3 9:1:0.5) to give **34** (0.153 g, 0.796 mmol, single diastereomer,17.7% over two steps) as a white waxy solid.

ν_{max} (cm^{-1}) 3283, 2973, 2867, 1599, 1451, 1016, 801, 591; **^{1}H-NMR (400 MHz, CDCl$_3$): δ_H** 7.14 (d, 2H, *J*= 7.8 Hz, *H*-6), 6.99 (d, 2H, *J*= 8.1 Hz, *H*-7), 3.38 (dq, 1H, *J*= 9.7, 6.3 Hz, *H*-2), 3.01 (d, 1H, *J*= 9.8 Hz, *H*-3), 2.35 (s, 3H, *H*-9), 2.11 (s, 6H, *H*-4), 0.87 (d, 3H, *J*= 6.2 Hz, *H*-1); **^{13}C-NMR (100 MHz CDCl$_3$): δ_C** 136.6 (*C*-5), 132.0 (*C*-8), 129.4 (*C*-6), 128.4 (*C*-7), 76.5 (*C*-3), 45.8 (*C*-2), 41.2 (*C*-4), 21.0 (*C*-9), 20.4 (*C*-1); **LR-ESI-MS:** $C_{12}H_{21}N_2$ [M+H]$^+$ *m/z* found 193.3, cald 193.2; **HR-ESI-MS:** $C_{12}H_{21}N_2$ [M+H]$^+$ *m/z* found 193.1706, cald 193.1705.

(1*S, 2*S**)-1-(4-chlorophenyl)-*N*$_1$,*N*$_1$-dimethylpropane-1,2-diamine (35)**

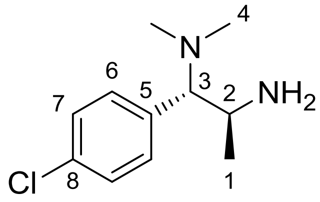

Under an inert atmsosphere (N_2) Raney Ni (2400) (0.234 g, 0.3 eq) was activated by washings with H_2O (x3) and then MeOH (x3, anhydrous) before a degassed solution of **31** (3.22 g, 13.27 mmol,1 eq) in MeOH (66 mL, 0.2 M, anhydrous) was added . The reaction vessel atmosphere was then exhanged with H_2 gas (x3). The reaction mixture was stirred under a H_2 atmsophere (double balloon, 1 bar) for 16 h. Upon reaction completion the mixture was filtered through celite, followed by filtration through a sintered frit to afford a colourless crude oil after drying. The oily residue was purified by Isolera Biotage LPLC

(DCM/MeOH 90:10 then DCM/MeOH/NH_3 9:1:0.5) to give **35** (0.691 g, 3.25 mmol, single diastereomer, 24.48% over three steps) as a colourless oil.

$ν_{max}$ (cm^{-1}) 2933, 2864, 2825, 2780, 1592, 1488, 1452, 1090, 1012, 842, 802, 701; **^{1}H-NMR (400 MHz, $CDCl_3$): $δ_H$** 7.30 (d, 2H, *J*= 8.6 Hz, *H*-7), 7.03 (d, 2H, *J*= 8.3 Hz, *H*-6), 3.36 (dq, 1H, *J*= 9.6, 6.3 Hz, *H*-2), 3.02 (d, 1H, *J*= 9.7 Hz, *H*-3), 2.10 (s, 6H, *H*-4), 0.86 (d, 3H, *J*= 6.2 Hz, *H*-1); **^{13}C-NMR (100 MHz $CDCl_3$): $δ_C$** 133.6 (*C*-8), 132.9 (*C*-5), 130.7 (*C*-6), 127.9 (*C*-7), 76.2 (*C*-3), 45.8 (*C*-2), 41.1 (*C*-4), 20.3 (*C*-1); **LR-ESI-MS:** $C_{11}H_{18}ClN_2$ $[M+H]^+$ *m/z* found 213.2, cald 213.1; **HR-ESI-MS:** $C_{11}H_{18}ClN_2$ $[M+H]^+$ *m/z* found 213.0792, cald 213.1159.

methyl 4-((1*S, 2*S**)-2-amino-1-(dimethylamino)propyl)benzoate (36)**

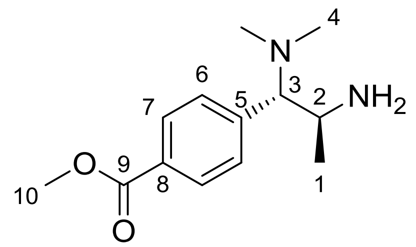

Under an inert atmsosphere (N_2) Raney Ni (2400) (0.066 g, 0.3 eq) was activated by washings with H_2O (x3) and then MeOH (x3, anhydrous) before a degassed solution of **26** (1 g, 3.76 mmol,1 eq) in MeOH (38 mL, 0.1 M, anhydrous) was added . The reaction vessel atmosphere was then exhanged with H_2 gas (x3). The reaction mixture was stirred under a H_2 atmsophere (double balloon, 1 bar) for 3 h. Upon reaction completion the mixture was filtered through celite, followed by filtration through a sintered frit to afford a colourless crude oil after drying. The oily residue was purified by Isolera Biotage LPLC (DCM/MeOH 90:10 then DCM/MeOH/NH_3 9:1:0.5) to give **36** (0.251 g, 1.062 mmol, single diastereomer, 28.3% over three steps) as a colourless oil.

$ν_{max}$ (cm^{-1}) 2950, 2826, 2782, 1716, 1435, 1311, 765; **^{1}H-NMR (400MHz, $CDCl_3$): $δ_H$** 8.01 (d, 2H, *J*= 8.1 Hz, *H*-7), 7.18 (d, 2H, *J*= 8.3 Hz, *H*-6), 3.92 (s, 3H, *H*-10), 3.42 (dq, 1H, *J*= 9.7, 6.3 Hz, *H*-2), 3.10 (d, 1H, *J*= 9.7 Hz, *H*-3), 2.13 (s, 6H, *H*-4), 0.87 (d, 3H, *J*= 6.4 Hz, *H*-1); **^{13}C-NMR (100 MHz $CDCl_3$): $δ_C$** 167.0 (*C*-9), 140.7 (*C*-5), 129.4 (*C*-7), 129.1 (*C*-8), 129.0 (*C*-6), 52.1 (*C*-3), 45.7 (*C*-2), 41.2 (*C*-4), 20.3 (*C*-1); **LR-ESI-MS:** $C_{13}H_{21}N_2O_2$ $[M+H]^+$ *m/z* found 237.2, cald 237.2; **HR-ESI-MS:** $C_{13}H_{21}N_2O_2$ $[M+H]^+$ *m/z* found 237.1560, cald 237.1603.

(1*S, 2*S**)-*N*$_1$,*N*$_1$-dimethyl-1-(4-(trifluoromethyl)phenyl)propane-1,2-diamine (37)**

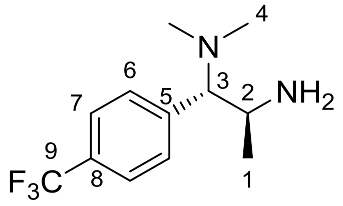

To a degassed stirred solution of **29** (2.486 g, 9 mmol,1 eq) in MeOH (60 mL, 0.15 M, anhydrous) was added Pd/C (10%) (0.287 g, 0.3 eq) and atmosphere was exhanged with H_2 gas (x3). The reaction mixture was stirred under a H_2 atmsophere (double balloon, 1 bar) for 16 h. Upon reaction completion the mixture was filtered through celite, followed by filtration through a sintered frit to afford a colourless crude oil after drying. The oily residue was purified by Isolera Biotage LPLC (DCM/MeOH 90:10 then DCM/MeOH/NH_3 9:1:0.5) to give **37** (0.153 g, 0.796 mmol, single diastereomer,14.2% over three steps) as a colourless oil.

$ν_{max}$ (cm^{-1}) 3222, 2936, 2785, 1670, 1163, 1032, 850; **^{19}F-{H}-NMR (376 MHz, $CDCl_3$): $δ_F$** -62.44; **^{1}H-NMR (400MHz, $CDCl_3$): $δ_H$** 7.60 (d, 2H, *J*= 8.1 Hz, *H*-7), 7.23 (d, 2H, *J*= 8.1 Hz, *H*-6), 3.42 (dq, 1H, *J*= 9.6, 6.3 Hz, *H*-2), 3.12 (d, 1H, *J*= 9.7 Hz, *H*-3), 2.13 (s, 6H, *H*-4), 0.87 (d, 3H, *J*= 6.2 Hz, *H*-1); **^{13}C-NMR (100 MHz $CDCl_3$): $δ_C$** 139.6 (*C*-5), 129.7 (*C*-7; *C*-6), 124.7 (q, *J*= 3.7 Hz, *C*-9), 76.5 (*C*-3), 45.7 (*C*-2), 41.2 (*C*-

4), 20.3 (*C*-1); **LR-ESI-MS:** $C_{12}H_{18}F_3N_2$ $[M+H]^+$ *m/z* found 247.1, cald 247.1; **HR-ESI-MS:** $C_{12}H_{18}F_3N_2$ $[M+H]^+$ *m/z* found 247.1428, cald 247.1422.

(1*S, 2*S**)-1-(4-methoxyphenyl)-*N*₁,*N*₁-dimethylpropane-1,2-diamine (38)**

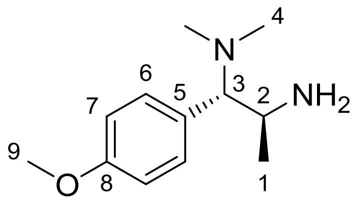

To a degassed stirred solution of **30** (2.556 g, 10.73 mmol,1 eq) in MeOH (72 mL, 0.15 M, anhydrous) was added Pd/C (10%) (0.342 g, 0.3 eq) and atmosphere was exhanged with H_2 gas (x3). The reaction mixture was stirred under a H_2 atmsophere (double balloon, 1 bar) for 16 h. Upon reaction completion the mixture was filtered through celite, followed by filtration through a sintered frit to afford a colourless crude oil after drying. The oily residue was purified by Isolera Biotage LPLC (DCM/MeOH 90:10 then DCM/MeOH/NH_3 9:1:0.5) to give **38** (0.334 g, 1.603 mmol, single diastereomer, 15% over three steps) as a colourless oil.

ν_{max} (cm^{-1}) 2931, 2825, 1608, 1509, 1246 , 1030, 840, 808, 593; **^{1}H-NMR (400MHz, $CDCl_3$): δ_H** 7.02 (d, 2H, *J*= 8.7 Hz, *H*-6), 6.87 (d, 2H, *J*= 8.7 Hz, *H*-7), 3.81 (s, 3H, *H*-9), 3.35 (dq, 1H, *J*= 9.7, 6.2 Hz, *H*-2), 2.99 (d, 1H, *J*= 9.7 Hz, *H*-3), 2.10 (s, 6H, *H*-4), 0.87 (d, 3H, *J*= 6.2 Hz, *H*-1); **^{13}C-NMR (100 MHz $CDCl_3$): δ_C** 158.6 (*C*-8), 130.5 (*C*-6), 127.3 (*C*-5), 113.0 (*C*-7), 76.2 (*C*-3), 55.1 (*C*-9), 46.0 (*C*-2), 41.2 (*C*-4), 20.4 (*C*-1); **LR-ESI-MS:** $C_{12}H_{21}N_2O$ $[M+H]^+$ *m/z* found 209.2, cald 209.2; **HR-ESI-MS:** $C_{12}H_{21}N_2O$ $[M+H]^+$ *m/z* found 209.1295, cald 209.1654.

(1*R*, 2*S*)-2-((3-methyl-[1,2,4]triazolo[3,4-a]phthalazin-6-yl)amino)-1-phenylpropan-1-ol (77)

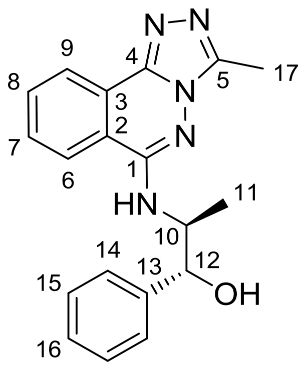

5 (1 g, 0.915 mmol, 1 eq) and (1*R*,2*S*)-(-)-Norephedrine (1.037 g, 6.86 mmol, 1.5 eq) were reacted according to general procedure **A**. The crude material was purified by Isolera Biotage LPLC (DCM/MeOH 90:10) to give **77** (0.413 g, 1.239 mmol, 27.1%) as a white solid.

Mpt: 239.6-241.6 °C; **ν_{max} (cm^{-1})** 3640, 3277, 2972, 1514, 1000, 701; **^{1}H-NMR (400 MHz, $CDCl_3$): δ_H** 8.47 (d, 1H, *J*= 8.2 Hz, *H*-9), 8.35 (d, 1H, *J*= 7.9 Hz, *H*-6), 7.89 (m, 1H, *H*-8), 7.81 (m, 1H, *H*-7), 7.49 (d, 2H, *J*= 7.3 Hz, *H*-14), 7.31 (m, 3H, *H*-15; N*H*), 7.18 (m, 1H, *H*-16, 5.45 (d, 1H, *J*= 4.8 Hz, O*H*), 5.07 (m, 1H, *H*-12), 4.32 (m, 1H, *H*-10), 1.17 (d, 3H, *J*= 6.7 Hz, *H*-11); **^{13}C-NMR (100 MHz, DMSO-*d*^{6}): δ_C** 150.4 (*C*-1), 146.0 (*C*-13), 143.9 (*C*-4), 141.0 (*C*-5), 132.7 (*C*-8), 130.0 (*C*-7), 127.8 (*C*-14), 126.5 (*C*-16), 125.9 (*C*-15), 124.6 (*C*-9), 123.3 (*C*-3), 122.4 (*C*-6), 118.3 (*C*-2), 72.1 (*C*-12), 53.0 (*C*-10), 13.2 (*C*-11), 9.4 (*C*-17); **LR-ESI-MS:** $C_{19}H_{20}N_5O$ $[M+H]^+$ *m/z* found 334.2, cald 334.2; **HR-ESI-MS:** $C_{19}H_{20}N_5O$ $[M+H]^+$ *m/z* found 334.1711, cald 334.1668.

(1*S, 2*S**)-1-(4-fluorophenyl)-*N*$_1$,*N*$_1$-dimethyl-*N*$_2$-(3-methyl-[1,2,4]triazolo[3,4-a]phthalazin-6-yl)propane-1,2-diamine (39)**

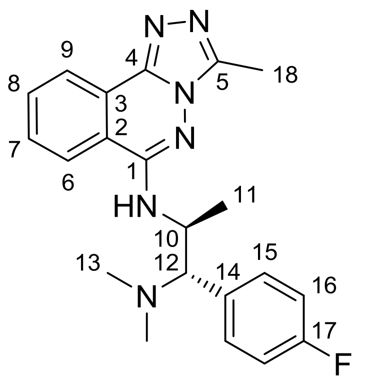

5 (365 mg, 1.669 mmol, 1 eq) and **33** (491 mg, 2.504 mmol, 1.5 eq) were reacted according to general procedure **A**. The crude material was purified by Isolera Biotage LPLC (DCM/MeOH 90:10) to give **39** (206 mg, 0.545 mmol, 32.7%) as an off white solid.

Mpt: 239.8-241.8 °C; **ν_{max} (cm^{-1})** 3453, 2953, 2814, 2773, 1593, 1500, 1246, 1028, 547, 695, 656, 446; **^{19}F-{H}-NMR (376 MHz, CDCl$_3$): δ_F** -115.67; **^{1}H-{F}-NMR (400 MHz, DMSO-*d*^{6}): δ_H** 8.40 (d, 1H, *J*= 7.7 Hz, *H*-9), 8.28 (d, 1H, *J*= 8.2 Hz, *H*-6), 7.94 (t, 1H, *J*= 7.7 Hz, *H*-8), 7.82 (m, 1H, *H*-7), 7.27 (d, 2H, *J*= 8.7 Hz, *H*-16), 7.18, (d, 2H, *J*= 7.2 Hz, *H*-15), 7.01 (d, 1H, *J*= 5.7 Hz, N*H*), 4.70 (m, 1H, *H*-10), 3.72 (d, 1H, *J*= 8.2 Hz, *H*-12), 2.62 (s, 3H, *H*-18), 2.13 (s, 6H, *H*-13), 1.10 (d, 3H, *J*= 6.2 Hz, *H*-11); **^{13}C-NMR (100 MHz, DMSO-*d*^{6}): δ_C** 161.5 (d, *J*= 242.1 Hz, *C*-17), 150.6 (*C*-1), 146.1 (*C*-4), 141.1 (*C*-5), 132.9 (*C*-8), 132.3 (*C*-14), 131.1 (d, *J*= 8.1 Hz, *C*-15), 130.2 (*C*-7), 124.2 (*C*-9), 123.4 (*C*-3), 122.6 (*C*-6), 118.3 (*C*-2), 114.5 (d, *J*= 21.3 Hz, *C*-16), 70.7 (*C*-12), 46.2 (*C*-10), 42.0 (*C*-13), 16.0 (*C*-11), 9.4 (*C*-18); **LR-ESI-MS:** $C_{21}H_{24}FN_6$ [M+H]$^+$ *m/z* found 379.3, cald 379.2; **HR-ESI-MS:** $C_{21}H_{24}FN_6$ [M+H]$^+$ *m/z* found 379.2070, cald 379.2046.

methyl 4-((1*S, 2*S**)-1-(dimethylamino)-2-((3-methyl-[1,2,4]triazolo[3,4-a]phthalazin-6-yl)amino)propyl)benzoate (40)**

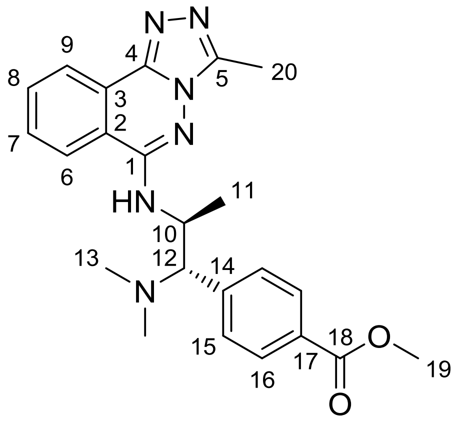

5 (156 mg, 0.713 mmol, 1 eq) and **36** (253 mg, 1.07 mmol, 1.5 eq) were reacted according to general procedure **A**. The crude material was purified by Isolera Biotage LPLC (DCM/MeOH 90:10 then DCM/MeOH/NH$_3$ 9:1:0.5) to give **40** (83 mg, 0.198 mmol 27.8%) as a white solid.

Mpt: 254.3-256.3 °C; **ν_{max} (cm^{-1})** 3256, 2926, 2775, 1722, 1593, 1432, 1101, 1019, 670, 659, 542; **^{1}H-NMR (400MHz, DMSO-*d*^{6}): δ_H** 8.40 (d, 1H, *J*= 7.9 Hz, *H*-9), 8.29 (d, 1H, *J*= 8.2 Hz, *H*-6), 7.94 (m, 3H, *H*-8; *H*-16), 7.81 (m, 1H, *H*-7), 7.36 (d, 2H, *J*= 8.3 Hz, *H*-15), 7.01 (d, 1H, *J*= 5.7 Hz, N*H*), 4.78 (m, 1H, *H*-10), 3.85 (s, 3H, *H*-19), 3.79 (d, 1H, *J*= 7.7 Hz, *H*-12), 2.62 (s, 3H, *H*-20), 2.15 (s, 6H, *H*-13), 1.10 (d, 3H, *J*= 6.6 Hz, *H*-11); **^{13}C-NMR (100 MHz DMSO-*d*^{6}): δ_C** 166.3 (*C*-18), 150.6 (*C*-1), 146.1 (*C*-4), 142.3 (*C*-14), 141.1 (*C*-5), 132.9 (*C*-8), 130.2 (*C*-7), 129.7 (*C*-16), 128.6 (*C*-15), 128.6 (*C*-17), 124.2 (*C*-9), 123.5 (*C*-3), 122.6 (*C*-6), 118.3 (*C*-2), 71.1 (*C*-12), 52.1 (*C*-19), 46.5 (*C*-10), 42.3 (*C*-13), 15.8 (*C*-11), 9.4 (*C*-20); **LR-ESI-MS:** $C_{23}H_{27}N_6O_2$ [M+H]$^+$ *m/z* found 419.4, cald 419.2; **HR-ESI-MS:** $C_{23}H_{27}N_6O_2$ [M+H]$^+$ *m/z* found 419.2233, cald 419.2195.

(1*S, 2*S**)-*N*$_1$,*N*$_1$-dimethyl-*N*$_2$-(3-methyl-[1,2,4]triazolo[3,4-a]phthalazin-6-yl)-1-(p-tolyl)propane-1,2-diamine (41)**

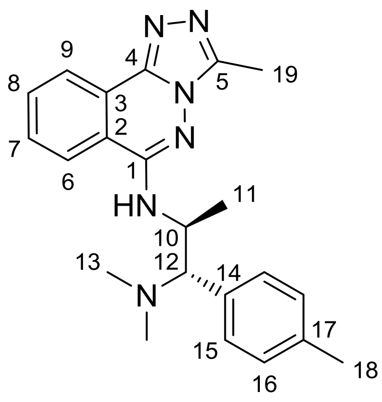

5 (75 mg, 0.343 mmol, 1 eq) and **34** (99 mg, 0.515 mmol, 1.5 eq) were reacted according to general procedure **A**. The crude material was purified by Isolera Biotage LPLC (DCM/MeOH 90:10 then DCM/MeOH/NH_3 9:1:0.5) to give **41** (43 mg, 0.115 mmol 33.4%) as a white solid.

Mpt: 189.6-191.6 °C; **ν_{max} (cm^{-1})** 3257, 2926, 2772, 1508, 1306, 1265, 1027, 802, 699; **^{1}H-NMR (400MHz, CDCl$_3$): δ_H** 8.61 (d, 1H, *J*= 7.9 Hz, *H*-9), 7.84 (m, 2H, *H*-6; *H*-8), 7.74 (m, 1H, *H*-7), 7.26 (m, 2H, *H*-15), 7.21 (d, 2H, *J*= 7.7 Hz, *H*-16), 6.86 (s, 1H, N*H*), 4.34 (m, 1H, *H*-10), 3.66 (m, 1H, *H*-12), 2.74 (s, 3H, *H*-18), 2.41 (s, 3H, *H*-18), 2.21 (s, 6H, *H*-13), 1.26 (d, 3H, *J*= 6 Hz, *H*-11); **^{13}C-NMR (100 MHz, CDCl$_3$): δ_C** 151.4 (*C*-1), 147.3 (*C*-4), 142.0 (*C*-5), 136.0 (*C*-14), 132.4 (*C*-8), 130.0 (*C*-15), 129.7 (*C*-7), 128.4 (*C*-16), 124.3 (*C*-9), 123.8 (*C*-3), 122.8 (*C*-6), 119.2 (*C*-2), 73.0 (*C*-12), 47.2 (*C*-10), 40.8 (*C*-13), 21.1 (*C*-18), 17.6 (*C*-11), 9.8 (*C*-19); **LR-ESI-MS:** $C_{22}H_{27}N_6$ [M+H]$^+$ *m/z* found 375.3, cald 375.3; **HR-ESI-MS:** $C_{22}H_{27}N_6$ [M+H]$^+$ *m/z* found 375.2315, cald 375.2297.

(1*S, 2*S**)-1-(4-chlorophenyl)-*N*$_1$,*N*$_1$-dimethyl-*N*$_2$-(3-methyl-[1,2,4]triazolo[3,4-a]phthalazin-6-yl)propane-1,2-diamine (42)**

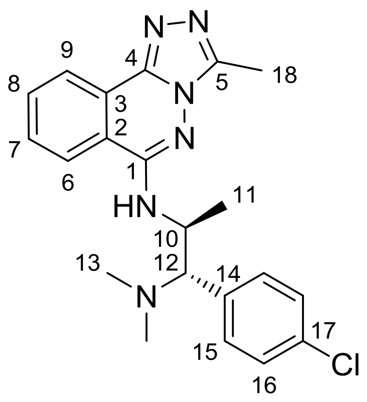

5 (410 mg, 1.875 mmol, 1 eq) and **35** (598 mg, 2.81 mmol, 1.5 eq) were reacted according to general procedure **A**. The crude material was purified by Isolera Biotage LPLC (DCM/MeOH 90:10 then DCM/MeOH/NH_3 9:1:0.5) to give **42** (43 mg, 0.115 mmol 33.4%) as a white solid.

Mpt: 158.8-160.8 °C; **ν_{max} (cm^{-1})** 3272, 2924, 1593, 1497, 1455, 1262, 1051, 696, 552, 461; **^{1}H-NMR (400 MHz, CDCl$_3$): δ_H** 8.63 (d, 1H, *J*= 7.8 Hz, *H*-9), 7.83 (m, 2H, *H*-6; *H*-8), 7.75 (m, 1H, *H*-7), 7.41 (d, 2H, *J*= 8.3 Hz, *H*-16), 7.21 (d, 2H, *J*= 8.4 Hz, *H*-15), 6.68 (s, 1H, N*H*), 4.30 (m, 1H, *H*-10), 3.61 (d, 1H, *J*= 10.1 Hz, *H*-12), 2.73 (s, 3H, *H*-18), 2.15 (s, 6H, *H*-13), 1.25 (d, 3H, *J*= 6 Hz, *H*-11); **^{13}C-NMR (100 MHz CDCl$_3$): δ_C** 151.2 (*C*-1), 147.3 (*C*-4), 142.0 (*C*-5), 133.9 (*C*-8), 132.5 (*C*-14), 132.0 (*C*-17), 130.9 (*C*-16), 130.0 (*C*-7), 128.4 (*C*-15), 124.3 (*C*-9), 123.8 (*C*-3), 122.5 (*C*-6), 119.1 (*C*-2), 72.7 (*C*-12), 47.1 (*C*-10), 40.9 (*C*-13), 17.4 (*C*-11), 9.8 (*C*-18); **LR-ESI-MS:** $C_{21}H_{24}ClN_6$ [M+H]$^+$ *m/z* found 395.3, cald 395.2; **HR-ESI-MS:** $C_{21}H_{24}ClN_6$ [M+H]$^+$ *m/z* found 395.1773, cald 395.1751.

(1*S, 2*S**)-*N*$_1$,*N*$_1$-dimethyl-*N*$_2$-(3-methyl-[1,2,4]triazolo[3,4-a]phthalazin-6-yl)-1-(4-(trifluoromethyl)phenyl)propane-1,2-diamine (43)**

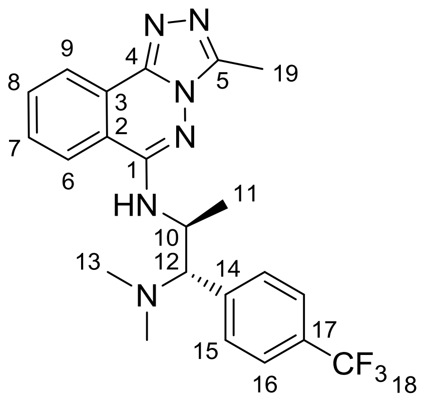

5 (170 mg, 0.778 mmol, 1 eq) and **37** (287 mg, 1.166 mmol, 1.5 eq) were reacted according to general procedure **A**. The crude material was purified by Isolera Biotage LPLC (DCM/MeOH 90:10 then DCM/MeOH/NH_3 9:1:0.5) to give **43** (53 mg, 0.124 mmol, 16%) as a white solid.

Mpt: 143.6-145.6 °C; **v_{max} (cm^{-1})** 3286, 2958, 2820, 1505, 1320, 1066, 699, 617; **^{19}F-NMR (376 MHz, $CDCl_3$): δ_F** -62.53; **^{1}H-NMR (400 MHz, $CDCl_3$): δ_H** 8.64 (d, 1H, *J*= 7.9 Hz, *H*-9), 7.87 (m, 1H, *H*-8), 7.81 (m, 1H, *H*-6), 7.75 (m, 1H, *H*-7), 7.70 (d, 2H, *J*= 8.1 Hz, *H*-16), 7.41 (d, 2H, *J*= 8.1 Hz, *H*-15), 6.62 (s, 1H, N*H*), 4.39 (m, 1H, *H*-10), 3.71 (d, 1H, *J*= 10.3 Hz, *H*-12), 2.74 (s, 3H, *H*-18), 2.21 (s, 6H, *H*-13), 1.26 (d, 3H, *J*= 6 Hz, *H*-11); **^{13}C-NMR (100 MHz $CDCl_3$): δ_C** 151.2 (*C*-1), 147.3 (*C*-4), 142.0 (*C*-5), 137.8 (*C*-14), 132.5 (*C*-8), 130.0 (q, *J*= 36.7 Hz, *C*-17), 129.9 (*C*-7), 125.1 (q, *J*= 3.7 Hz, *C*-16), 124.4 (*C*-9), 124.1 (q, *J*= 105.6 Hz, *C*-18), 123.9 (*C*-3), 122.7 (*C*-15), 122.5 (*C*-6), 119.1 (*C*-2), 72.9 (*C*-12), 47.1 (*C*-10), 40.9 (*C*-13), 17.4 (*C*-11), 9.8 (*C*-19); **LR-ESI-MS:** $C_{22}H_{24}F_3N_6$ $[M+H]^+$ *m/z* found 429.2, cald 429.2; **HR-ESI-MS:** $C_{22}H_{24}F_3N_6$ $[M+H]^+$ *m/z* found 429.2061, cald 429.2015.

(1*S, 2*S**)-1-(4-methoxyphenyl)-*N*1,*N*$_1$-dimethyl-*N*$_2$-(3-methyl-[1,2,4]triazolo[3,4-a]phthalazin-6-yl)propane-1,2-diamine (44)**

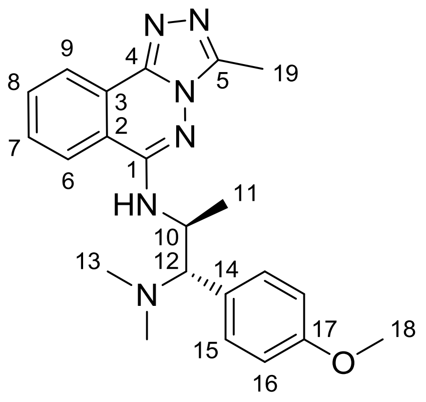

5 (234 mg, 1.07 mmol, 1 eq) and **38** (334 mg, 1.605 mmol, 1.5 eq) were reacted according to general procedure **A**. The crude material was purified by Isolera Biotage LPLC (DCM/MeOH 90:10 then DCM/MeOH/NH_3 9:1:0.5) to give **44** (330 mg, 1.07 mmol, 79%) as a white solid.

Mpt: 171.9-173.9 °C; **v_{max} (cm^{-1})** 3318, 2929, 2778, 1509, 1263, 1177, 1028, 769; **^{1}H-NMR (400 MHz, $CDCl_3$): δ_H** 8.63 (d, 1H, *J*= 7.6 Hz, *H*-9), 7.84 (m, 2H, *H*-6; *H*-8), 7.75 (m, 1H, *H*-7), 7.20 (d, 2H, *J*= 8.3 Hz, *H*-15), 6.98 (d, 2H, *J*= 8.6 Hz, *H*-16), 6.85 (s, 1H, N*H*), 4.28 (m, 1H, *H*-10), 3.86 (s, 3H, *H*-18), 3.57 (d, 2H, *J*= 9.8 Hz, *H*-12), 2.74 (s, 3H, *H*-19), 2.17 (s, 6H, *H*-13), 1.26 (d, 3H, *J*= 6 Hz, *H*-11); **^{13}C-NMR (100 MHz, $CDCl_3$): δ_C** 159.3 (*C*-17), 151.4 (*C*-1), 147.3 (*C*-4), 142.0 (*C*-5, 132.4 (*C*-8), 130.7 (*C*-14), 130.0 (*C*-7), 125.4 (*C*-15), 124.3 (*C*-9), 123.8 (*C*-3), 122.6 (*C*-6), 119.3 (*C*-2), 113.5 (*C*-16), 72.7 (*C*-10), 55.2 (*C*-18), 47.4 (*C*-12), 40.8 (*C*-13), 17.5 (*C*-11), 9.8 (*C*-19); **LR-ESI-MS:** $C_{22}H_{27}N_6O$ $[M+H]^+$ *m/z* found 391.4, cald 391.2; **HR-ESI-MS:** $C_{22}H_{27}N_6O$ $[M+H]^+$ *m/z* found 391.2262, cald 391.2246.

(1*S, 2*S**)-*N*$_1$,*N*$_1$-dimethyl-*N*$_2$-(3-methyl-[1,2,4]triazolo[3,4-a]phthalazin-6-yl)-1-phenylpropane-1,2-diamine (45)**

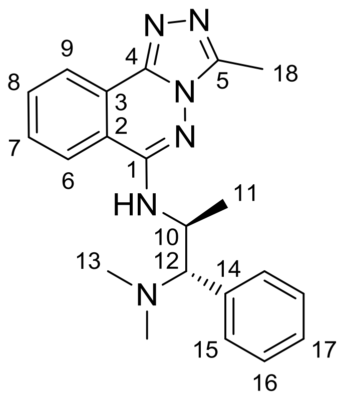

5 (110 mg, 0.503 mmol, 1 eq) and **93** (135 mg, 0.755 mmol, 1.5 eq) were reacted according to general procedure **A**. The crude material was purified by Isolera Biotage LPLC (DCM/MeOH 90:10 then DCM/MeOH/NH_3 9:1:0.5) to give **45** (34 mg, 0.095 mmol, 18.9%) as a white solid.

The *rac*emic mixture **45** (16 mg) was resolved by preparative chiral HPLC (Chiralpak®OG, CH/IPA 85:15, λ 220 nm, 3 mL/min) to give both enantiomers of **45** which were analysed by analytical chiral HPLC to ascertain optical purity:

***L*-45** (2.8 mg, Chiralpak®OG CH/IPA 9:1, 1 mL/min, R_t= 43.88 min, 90% *ee*) $[\alpha]_D^{20}$ -0.068 (0.5 *c* in $CHCl_3$)

***D*-45** (5.8 mg, Chiralpak®OG CH/IPA 9:1, 1 mL/min, R_t= 37.13 min, 99% *ee*) $[\alpha]_D^{20}$ +0.117 (0.5 *c* in $CHCl_3$)

Mpt: 182.7-184.7 °C; **v_{max} (cm^{-1})** 3242, 3078, 2929, 2771, 1510, 1148, 753, 698, 546; **^{1}H-NMR (400 MHz, DMSO-*d*^{6}): δ_H** 8.41 (d, 1H, *J*= 7.7 Hz, *H*-9), 8.29 (d, 1H, *H*-6), 7.94 (m, 1H, *H*-8), 7.83 (m, 1H, *H*-7), 7.37 (m, 3H, *H*-15; *H*-17), 7.24 (d, 2H, *J*= 7.0 Hz, *H*-16), 7.08 (d, 1H, *J*= 5.9 Hz, N*H*), 4.71 (m, 1H, *H*-10), 3.72 (d, 1H, *J*= 8.6 Hz, *H*-12), 2.62 (s, 3H, *H*-18), 2.12 (s, 6H, *H*-13), 1.11 (d, 3H, *J*= 6.4 Hz, *H*-11); **^{13}C-NMR (100 MHz, DMSO-*d*^{6}): δ_C** 150.7 (*C*-1), 146.1 (*C*-4), 141.1 (*C*-5), 135.7 (*C*-14), 132.9 (*C*-8), 130.2 (*C*-7), 129.4 (*C*-15), 127.8 (*C*-16), 127.3 (*C*-17), 124.1 (*C*-9), 123.4 (*C*-3), 122.5 (*C*-6), 118.3 (*C*-2), 71.6 (*C*-12), 46.5 (*C*-10), 41.8 (*C*-13), 16.5 (*C*-11), 9.4 (*C*-18); **LR-ESI-MS**: $C_{21}H_{25}N_6$ $[M+H]^+$ *m/z* found 361.3, cald 361.2; **HR-ESI-MS**: $C_{21}H_{25}N_6$ $[M+H]^+$ *m/z* found 361.2161, cald 361.2141.

4-((1*S, 2*S**)-1-(dimethylamino)-2-((3-methyl-[1,2,4]triazolo[3,4-a]phthalazin-6-yl)amino)propyl)benzoic acid (78)**

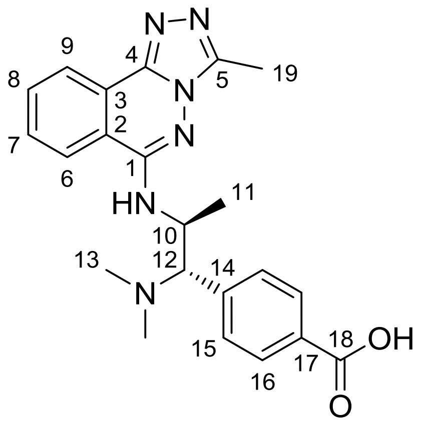

To a stirred solution of **40** (38 mg, 0.091 mmol, 1 eq) in THF/H_2O (908 µL, 2:1, 0.1 M) was added LiOH.H_2O (33 mg, 1.362 mmol, 15 eq) portion wise and allowed to stir for 16 h. Upon reaction completion the mixture was carefully quenched with 1N HCl (1.3 mL, 1.362 mmol, 15 eq) and concentrated down to dryness. The crude material was suspended in DCM/MeOH (9:1, 50 mL) and sonicated prior to being filtered through a sintered frit. The filtrate was concetrated down to give **78** (39 mg, 0.097 mmol, *quant.*) as a white solid.

Mpt: 256.3-258.3 °C; **v_{max} (cm^{-1})** 3276, 2918, 1698, 1515, 1268, 1116, 700; **^{1}H-NMR (400MHz, DMSO-*d*^{6}): δ_H** 8.42 (m, 2H, *H*-6; *H*-9), 8.05 (d, 1H, *J*= 7.9 Hz, *H*-16), 7.97 (t, 1H, *J*= 7.6 Hz, *H*-8), 7.85 (t, 1H, *J*= 7.7 Hz, *H*-7), 7.64 (m, 3H, *H*-15, N*H*), 5.15 (br-s, 1H, *H*-12), 4.85 (br-s, 1H, *H*-10), 4.78 (m, 1H, *H*-10), 3.85 (s, 3H, *H*-19), 3.79 (d, 1H, *J*= 7.7 Hz, *H*-12), 2.68 (m, 9H, *H*-13; *H*-19), 1.18 (d, 3H, *J*= 6.5 Hz, *H*-11); **^{13}C-NMR (100MHz, DMSO-*d*^{6}): δ_C** 170.3 (*C*-18), 150.9 (*C*-1), 146.1 (*C*-4), 141.1 (*C*-5), 136.6 (*C*-14), 132.8 (*C*-8), 130.3 (*C*-7),

128.7 (*C*-16), 128.4 (*C*-15), 124.5 (*C*-9), 123.4 (*C*-3), 122.5 (*C*-6), 118.5 (*C*-2), 71.5 (*C*-12), 46.6 (*C*-10), 41.7 (*C*-13), 16.8 (*C*-11), 9.5 (*C*-19); **LR-ESI-MS:** $C_{22}H_{25}N_6O_2$ [M+H]$^+$ *m/z* found 405.3, cald 405.2; **HR-ESI-MS:** $C_{22}H_{25}N_6O_2$ [M+H]$^+$ *m/z* found 405.2067, cald 405.2039.

tert-butyl (4-(4-((1*S, 2*S**)-1-(dimethylamino)-2-((3-methyl-[1,2,4]triazolo[3,4-a]phthalazin-6-yl)amino)propyl)benzamido)butyl)carbamate (79)**

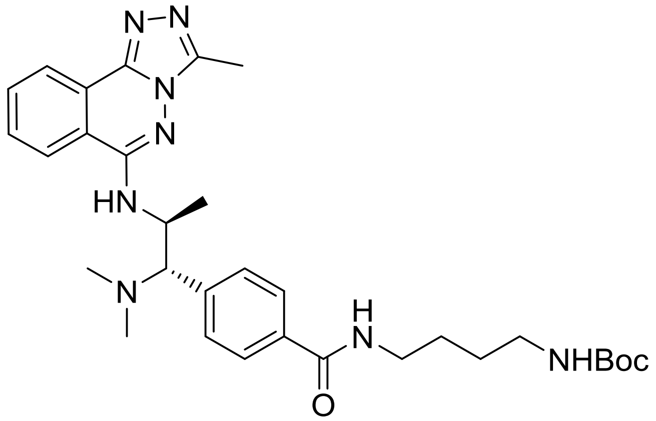

To a stirred solution of **78** (24 mg, 0.059 mmol, 1 eq) in DMF (1.1 mL, 0.05 M, anhydrous) under an inert atmsosphere (N_2) was added TEA (19 µL, 0.142 mmol, 2.4 eq) and HATU (27 mg, 0.071 mmol, 1.2 eq). The solution was allowed to stir for 10 min before tert-butyl (4-aminobutyl)carbamate (12.5 µL, 0.065 mmol, 1.1 eq) was added. The reaction mixture was allowed to stir for 16 h before being concentrated to dryness and flushed through a silica pad with DCM/MeOH (9:1) to remove impurities and **79** was submitted to the next step without further purification.

***N*-(4-aminobutyl)-4-((1*S**, 2*S**)-1-(dimethylamino)-2-((3-methyl-[1,2,4]triazolo[3,4-a]phthalazin-6-yl)amino)propyl)benzamide (80)**

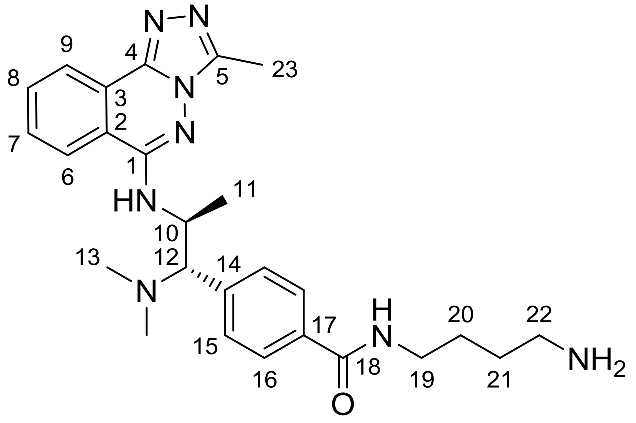

To a stirred solution of **79** (408 mg, 0.710 mmol, 1 eq) in DCM (35 mL, 0.02 M anhydrous) was added TFA (1.4 mL, 17.75 mmol, 25 eq). The reaction mixture was allowed to stir for 16 h at room temperature before being concentrated down to dryness. The crude residue was dissolved in MeOH and purified by SC-X ion exchange chromatography (MeOH to 7N NH_3 in MeOH) to give **80** (150 mg, 0.317 mmol, 45%) as a yellow oil.

ν_{max} (cm^{-1}) 2941, 1670, 1637, 1527, 1420, 1171, 1018, 704; **^{1}H-NMR (400MHz, DMSO-*d*^{6}): δ_H** 8.71 (m, 1H, CON*H*), 8.45 (m, 1H, *H*-9), 8.37 (m, 1H, *H*-6), 8.01 (m, 3H, *H*-18; *H*-16), 7.91 (m, 1H, *H*-7), 7.74 (m, 2H, *H*-15), 7.60 (m, 1H, N*H*), 5.17 (m, 1H, *H*-10), 4.77 (m, 1H, *H*-12), 3.32 (d, 2H, *J*= 5.5 Hz, *H*-19), 2.90 (m, 2H, *H*-22), 2.71 (s, 3H, *H*-23), 2.54 (s, 6H, *H*-13), 1.59 (m, 4H, *H*-20; *H*-21), 1.13 (d, 3H, *J*= 6.4 Hz, *H*-11); **^{13}C-NMR (100MHz DMSO-*d*^{6}): δ_C** 165.4 (*C*-18), 158.2 (*C*-, 157.9 (*C*-, 151.0 (*C*-1), 146.4 (*C*-4), 141.1 (*C*-5), 136.6 (*C*-14), 132.8 (*C*-8), 130.9 (*C*-7), 130.2 (*C*-16), 127.7 (*C*-15), 123.3 (*C*-9), 118.5 (*C*-3), 118.1 (*C*-6), 115.1 (*C*-2), 71.5 (*C*-12), 43.1 (*C*-10), 40.4 (*C*-13), 38.7 (*C*-22), 38.6 (*C*-19), 26.2 (*C*-20), 24.6 (*C*-21), 17.116.8 (*C*-11), 9.5 (*C*-23); **LR-ESI-MS:** $C_{26}H_{35}N_8O$ [M+H]$^+$ *m/z* found 475.3, cald 475.3; **HR-ESI-MS:** $C_{26}H_{35}N_8O$ [M+H]$^+$ *m/z* found 475.2390, cald 475.2934.

2-((3-methyl-[1,2,4]triazolo[3,4-a]phthalazin-6-yl)amino)-1-phenylethan-1-one (81)

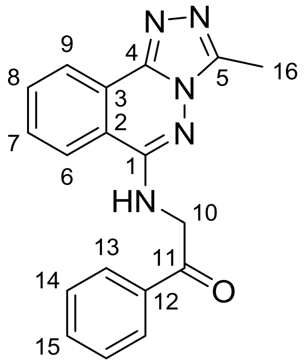

To a stirred solution of **96** (2 g, 6.26 mmol, 1 eq) in DMF (42 mL, 0.15 M, anhydrous) was added Pyridinium Dichromate (11.78 g, 31.3 mmol, 5 eq) portion wise under an inert atmosphere (N_2). The reaction mixture was stirred at room temperature for 48 h after which ice was added to the reaction mixture. A brown precipitate formed which was filtered off and dissolved in $CHCl_3$/MeOH (9:1). The mixture was then filtered through a celite plug to give ketone **81** (1.017 g, 3.20 mmol, 51%) as an off white solid.

Mpt: 156.6-158.6 °C; **ν_{max} (cm^{-1})** 3220, 3074, 2919, 1701, 1571, 1473, 1002, 965, 667; **^{1}H-NMR (400MHz, DMSO-*d^6*): δ_H** 8.40 (d, 2H, *J*= 8.2 Hz, *H*-13), 8.28 (t, 1H, *J*= 5.6 Hz, N*H*), 8.10 (m, 2H, *H*-6; *H*-9), 7.96 (m, 1H, *H*-8), 7.88 (m, 1H, *H*-7), 7.69 (m, 1H, *H*-15), 7.59 (t, 1H, *J*= 7.9 Hz, *H*-14), 4.90 (d, 1H, *J*= 5.6 Hz, *H*-10), 2.25 (s, 3H, *H*-16); **^{13}C-NMR (100MHz, DMSO-*d^6*): δ_C** 196.3 (*C*-11), 151.0 (*C*-1), 145.8 (*C*-4), 141.1 (*C*-5), 135.8 (*C*-12), 133.4 (*C*-15, 133.1 (*C*-8), 130.3 (*C*-7), 128.8 (*C*-13, 127.8 (*C*-14), 124.3 (*C*-9), 123.3 (*C*-3), 122.5 (*C*-6), 118.0 (*C*-2), 79.2 (*C*-10), 8.9 (*C*-16); **LR-ESI-MS:** $C_{18}H_{16}N_5O$ $[M+H]^+$ *m/z* found 318.3, cald 318.1; **HR-ESI-MS:** $C_{18}H_{16}N_5O$ $[M+H]^+$ *m/z* found 318.1387, cald 318.1355.

1-chloro-4-hydrazinylphthalazine (82)

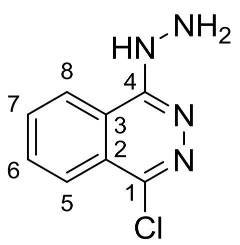

To a stirred solution of 1,4-dichlorophthalazine **4** (884 mg, 4.44 mmol, 1 eq) in EtOH (22 mL, 0.2 M) was added hydrazine.monohydrate (1.637 mL, 33.8 mmol, 7.6 eq) and the reaction mixture was warmed to 120 °C. After 10 mins the reaction mixture completely precipitated out to a yellow solid. The reaction mixture was cooled and filtered to give a yellow solid which was washed with cold Et_2O (25 mL x 3) and dried to give **82** (862 mg, 4.43 mmol, *quant.*) as a yellow solid.

Mpt: 174.5-176.5 °C; **ν_{max} (cm^{-1})** 3240, 3157, 2944, 1640, 1515, 1420, 1294, 1102, 988, 757; **^{1}H-NMR (400MHz, DMSO-*d^6*): δ_H** 8.25 (m, 1H, *H*-8), 7.92 (m, 3H, *H*-5; *H*-6; *H*-7); **^{13}C-NMR (100MHz DMSO-*d^6*): δ_C** 132.6 (*C*-6, *C*-7), 124.7 (*C*-2), 124.5 (*C*-5), 122.5 (*C*-3, *C*-8); **LR-ESI-MS:** $C_8H_8ClN_4$ $[M+H]^+$ *m/z* found 195.0, cald 195.0; **HR-ESI-MS:** $C_8H_8ClN_4$ $[M+H]^+$ *m/z* found 195.0096, cald 195.0437.

(1*S*, 2*S*)-*N_1*,*N_1*-dimethyl-*N_2*-(3-(trifluoromethyl)-[1,2,4]triazolo[3,4-a]phthalazin-6-yl)cyclohexane-1,2-diamine (83)

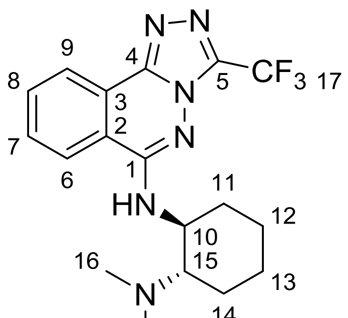

6 (300 mg, 1.1 mmol, 1 eq) and (1*S*,2*S*)-*N_1*,*N_1*-dimethylcyclohexane-1,2-diamine (313 mg, 2.201 mmol, 2 eq) were reacted according to general procedure **A**. The crude material was purified by Isolera Biotage LPLC (DCM/MeOH 90:10 then DCM/MeOH/NH_3 9:1:0.5) to give **83** (194 mg, 0.514 mmol 47%) as a white solid.

Mpt: 216.6-218.6 °C; **ν_{max} (cm^{-1})** 3312, 2939, 2864, 1530, 1477, 1125, 1024, 991, 735; **^{19}F-NMR (376 MHz, $CDCl_3$): δ_F** -64.22; **^{1}H-NMR (400MHz, $CDCl_3$): δ_H** 8.60 (dd, 1H, *J*= 7.9, 0.7 Hz, *H*-9), 8.06 (d, 1H, *J*= 7.9 Hz, *H*-6), 7.86 (m, 1H, *H*-8), 7.79 (m, 1H, *H*-7), 7.07 (br-s, 1H, N*H*),

3.67 (t, 1H, *J*= 10.5 Hz, *H*-10), 2.87 (m, 2H, *H*-11"; *H*-15), 2.41 (s, 6H, *H*-16), 1.99 (m, 2H, *H*-14), 1.81 (d, 1H, *J*= 11 Hz, *H*-11'), 1.35 (m, 4H, *H*-12; *H*-13); **^{13}C-NMR (100MHz CDCl$_3$): δ$_C$** 152.4 (*C*-1), 143.9 (*C*-4), 139.6 (q, *J*= 40.4 Hz, *C*-5), 132.9 (*C*-8), 131.3 (*C*-7), 124.0 (*C*-9), 123.5 (*C*-3), 122.9 (*C*-6), 119.3 (*C*-2), 118.7 (q, *J*= 270.7 Hz, *C*-17), 66.4 (*C*-15), 53.1 (*C*-10), 39.8 (*C*-16), 31.0 (*C*-11), 25.2 (*C*-14), 24.3 (*C*-13, 21.4 (*C*-12); **LR-ESI-MS:** $C_{18}H_{22}F_3N_6$ [M+H]$^+$ *m/z* found 379.2, cald 379.2; **HR-ESI-MS:** $C_{18}H_{22}F_3N_6$ [M+H]$^+$ *m/z* found 379.1913, cald 379.1858.

(1*S, 2*S**)-*N*$_1$,*N*$_1$-dimethyl-*N*$_2$-(3-(trifluoromethyl)-[1,2,4]triazolo[3,4-a]phthalazin-6-yl)cyclopentane-1,2-diamine (84)**

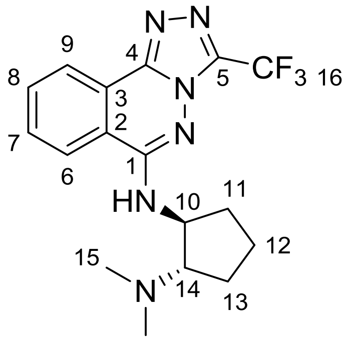

6 (300 mg, 1.1 mmol, 1 eq) and (1*S**,2*S**)-*N*$_1$,*N*$_1$-dimethylcyclopentane-1,2-diamine (212 mg, 1.651 mmol, 1.5 eq) were reacted according to general procedure **A**. The crude material was purified by Isolera Biotage LPLC (DCM/MeOH 90:10 then DCM/MeOH/NH$_3$ 9:1:0.5) to give **84** (306 mg, 0.84 mmol, 76%) as a white solid.

Mpt: 191.1-193.1 °C; **ν$_{max}$ (cm^{-1})** 3483, 3303, 2945, 2775, 1567, 1459, 1184, 1146, 979, 703; **^{19}F-NMR (376 MHz, DMSO-*d^6*): δ$_F$** -63.12; **^{1}H-NMR (400MHz, DMSO-*d^6*): δ$_H$** 8.53 (d, 1H, *J*= 7.8 Hz, *H*-9), 8.49 (dd, 1H, *J*= 7.8, 1.2 Hz, *H*-6), 8.01 (m, 1H, *H*-8), 7.95 (m, 1H, *H*-7), 7.77 (d, 1H, *J*=7.7 Hz, N*H*), 4.31 (m, 1H, *H*-10), 2.97 (m, 1H, *H*-14), 2.18 (s, 6H, *H*-15), 2.08 (m, 1H, *H*-11"), 1.83 (m, 1H, *H*-13"), 1.61 (m, 1H, *H*-11'; *H*-13'; *H*-12); **^{13}C-NMR (100MHz DMSO-*d^6*): δ$_C$** 151.9 (*C*-1), 143.7 (*C*-4), 138.2 (q, *J*= 39.6 Hz, *C*-5), 133.3 (*C*-8), 131.6 (*C*-7), 124.6 (*C*-9), 123.2 (*C*-3), 122.1 (*C*-6), 118.9 (*C*-2), 118.7 (q, *J*= 269.2 Hz, *C*-16), 70.7 (*C*-10), 54.6 (*C*-14), 42.5 (*C*-15), 31.3 (*C*-11), 26.9 (*C*-13), 22.0 (*C*-12); **LR-ESI-MS:** $C_{17}H_{20}F_3N_6$ [M+H]$^+$ *m/z* found 365.3, cald 365.2; **HR-ESI-MS:** $C_{17}H_{20}F_3N_6$ [M+H]$^+$ *m/z* found 365.1742, cald 365.1702.

***N*-benzyl-3-(trifluoromethyl)-[1,2,4]triazolo[3,4-a]phthalazin-6-amine (85)**

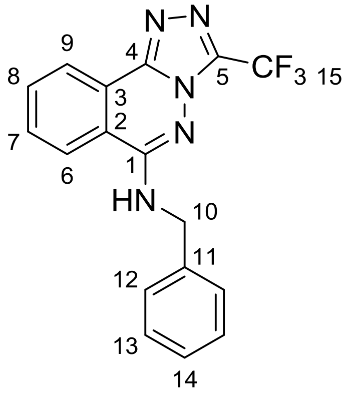

6 (200 mg, 0.734 mmol, 1 eq) and benzylamine (157 mg, 1.467 mmol, 2 eq) were reacted according to general procedure **A**. The crude material was purified by Isolera Biotage LPLC (DCM/MeOH 90:10) to give **85** (134 mg, 0.391 mmol, 53%) as a white solid.

Mpt: 166.8-168.8 °C; **ν$_{max}$ (cm^{-1})** 3888, 1562, 1527, 1289, 1116, 1023, 973, 721, 699, 516; **^{19}F-NMR (376 MHz, CDCl$_3$): δ$_F$** -64.07; **^{1}H-NMR (400MHz, CDCl$_3$): δ$_H$** 8.70 (d, 1H, *J*= 7.9 Hz, *H*-9), 7.92 (ddd, 1H, *J*= 8.1, 5.3, 3 Hz, *H*-6), 7.81 (m, 2H, *H*-8, *H*-7), 7.49 (d, 2H, *J*= 7.2 Hz, *H*-12), 7.38 (m, 3H, *H*-13; *H*-14), 5.61 (br-s, 1H, N*H*), 4.73 (d, 2H, *J*= 5.1 Hz, *H*-10); **^{13}C-NMR (100MHz CDCl$_3$): δ$_C$** 151.5 (*C*-1), 143.9 (*C*-11), 137.4 (*C*-4), 133.2 (*C*-8), 131.3 (*C*-7), 128.9 (*C*-12), 128.6 (*C*-13), 128.1 (*C*-14), 126.5 (*C*-14), 124.6 (*C*-9), 123.3 (*C*-3), 122.2 (*C*-6), 118.6 (q, *J*= 269.2 Hz, *C*-15), 118.5 (*C*-2), 46.5 (*C*-10); **LR-ESI-MS:** $C_{17}H_{13}F_3N_5$ [M+H]$^+$ *m/z* found 344.1, cald 344.1; **HR-ESI-MS:** $C_{17}H_{13}F_3N_5$ [M+H]$^+$ *m/z* found 344.1142, cald 344.1123.

***N*₁,*N*₁,4-trimethyl-*N*₂-(3-(trifluoromethyl)-[1,2,4]triazolo[3,4-a]phthalazin-6-yl)pentane-1,2-diamine (86)**

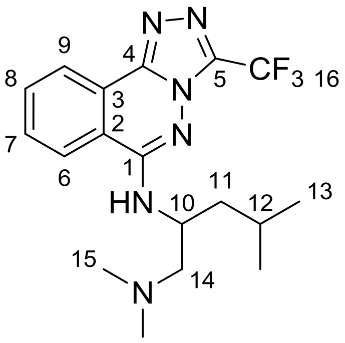

6 (300 mg, 1.1 mmol, 1 eq) and N_1,N_1-1,4-trimethylpentane-1,2-diamine (238 mg, 1.651 mmol, 1.5 eq) were reacted according to general procedure **A**. The crude material was purified by Isolera Biotage LPLC (DCM/MeOH 90:10 then DCM/MeOH/NH_3 9:1:0.5) to give **86** (108 mg, 0.283 mmol, 26%) as a white solid.

Mpt: 218.5-220.5 °C; **ν_{max} (cm^{-1})** 3286, 2957, 2821, 1552, 1493, 1148, 1044, 979, 709, 703; **^{19}F-NMR (376 MHz, DMSO-*d*^{6}): δ_F** -63.29; **^{1}H-NMR (400MHz, DMSO-*d*^{6}): δ_H** 8.49 (m, 2H, *H*-9; *H*-6), 8.01 (m, 1H, *H*-8), 7.95 (m, 1H, *H*-7), 7.95 (m, 1H, *H*-7), 7.55 (d, 1H, *J*= 8.4 Hz, N*H*), 4.42 (m, 1H, *H*-10), 2.51 (m, 1H, *H*-14”), 2.28 (dd, 1H, *J*= 11.8, 6.8 Hz, *H*-14’), 2.18 (s, 6H, *H*-15), 1.66 (m, 2H, *H*-11”; *H*-12), 1.48 (m, 1H, *H*-11’), 0.85 (d, 6H, *J*= 7.1 Hz, *H*-13); **^{13}C-NMR (100MHz DMSO-*d*^{6}): δ_C** 152.5 (*C*-1), 143.6 (*C*-4), 138.1 (q, *J*= 40.3 Hz, *C*-5), 133.4 (*C*-8), 131.6 (*C*-7), 124.5 (*C*-9), 123.3 (*C*-3), 122.2 (*C*-6), 118.7 (*C*-2), 118.7 (q, *J*= 270 Hz, *C*-16), 63.5 (*C*-10), 47.0 (*C*-14), 45.4 (*C*-15), 41.6 (*C*-11), 24.5 (*C*-12), 23.2 (*C*-13”), 21.7 (*C*-13’); **LR-ESI-MS:** $C_{18}H_{24}F_3N_6$ [M+H]$^+$ *m/z* found 381.3, cald 381.2; **HR-ESI-MS:** $C_{18}H_{24}F_3N_6$ [M+H]$^+$ *m/z* found 381.2061, cald 381.2015.

***N*₁,*N*₁-dimethyl-*N*₂-(3-(trifluoromethyl)-[1,2,4]triazolo[3,4-a]phthalazin-6-yl)ethane-1,2-diamine (87)**

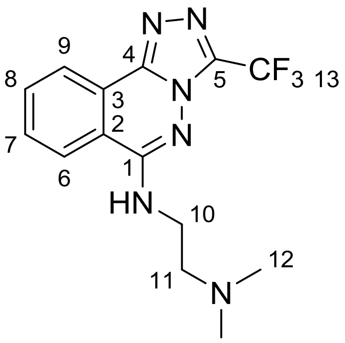

6 (300 mg, 1.1 mmol, 1 eq) and N_1,N_1-dimethylethane-1,2-diamine (146 mg, 1.651 mmol, 1.5 eq) were reacted according to general procedure **A**. The crude material was purified by Isolera Biotage LPLC (DCM/MeOH 90:10 then DCM/MeOH/NH_3 9:1:0.5) to give **87** (339 mg, 1.046 mmol, 95%) as a white solid.

Mpt: 166.1-168.1 °C; **ν_{max} (cm^{-1})** 3380, 2948, 2768, 1551, 1499, 1140, 1035, 979, 720, 693; **^{19}F-NMR (376 MHz, $CDCl_3$): δ_F** -64.19; **^{1}H-NMR (400MHz, $CDCl_3$): δ_H** 8.64 (d, 1H, *J*= 7.9 Hz, *H*-9), 7.90 (m, 2H, *H*-6; *H*-8), 7.82 (m, 1H, *H*-7), 6.50 (br-s, 1H, N*H*), 3.61 (m, 2H, *H*-10), 2.75 (t, 2H, *J*= 5.8 Hz, *H*-11), 2.39 (s, 6H, *H*-12); **^{13}C-NMR (100MHz $CDCl_3$): δ_C** 152.1 (*C*-1), 144.0 (*C*-4), 139.8 (q, *J*= 41.1 Hz, *C*-5), 133.0 (*C*-8), 131.3 (*C*-7), 124.3 (*C*-9), 123.0 (*C*-3), 122.8 (*C*-6), 118.8 (*C*-2), 118.6 (q, *J*= 270.0 Hz, *C*-16), 56.8 (*C*-10), 45.0 (*C*-11), 38.6 (*C*-12); **LR-ESI-MS:** $C_{14}H_{16}F_3N_6$ [M+H]$^+$ *m/z* found 325.3, cald 325.1; **HR-ESI-MS:** $C_{14}H_{16}F_3N_6$ [M+H]$^+$ *m/z* found 325.1396, cald 325.1389.

N_1,N_1-dimethyl-N_2-(3-(trifluoromethyl)-[1,2,4]triazolo[3,4-a]phthalazin-6-yl)butane-1,2-diamine (88)

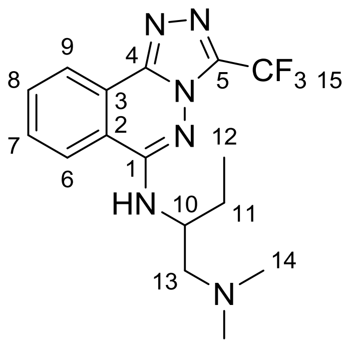

6 (300 mg, 1.1 mmol, 1 eq) and N_1,N_1-dimethylbutane-1,2-diamine (166 mg, 1.431 mmol, 1.3 eq) were reacted according to general procedure **A**. The crude material was purified by Isolera Biotage LPLC (DCM/MeOH 90:10 then DCM/MeOH/NH_3 9:1:0.5) to give **88** (299 mg, 0.849 mmol, 77%) as a white solid.

Mpt: 175.1-177.1 °C; **ν_{max} (cm^{-1})** 3298, 2937, 2821, 2773, 1554, 1492, 1287, 1136, 978, 781; **^{19}F-NMR (376 MHz, DMSO-*d^6*): δ_F** -63.35; **^{1}H-NMR (400MHz, DMSO-*d^6*): δ_H** 8.49 (d, 1H, *J*= 8.1 Hz, *H*-9), 8.44 (d, 1H, *J*= 7.7 Hz, *H*-6), 7.95 (m, 1H, *H*-8), 7.89 (m, 1H, *H*-7), 7.49 (d, 1H, *J*= 7.8 Hz, N*H*), 4.11 (m, 1H, *H*-10), 2.54 (m, 1H, *H*-13”), 2.26 (m, 1H, *H*-13’), 2.15 (s, 6H, *H*-14), 1.76 (m, 1H, *H*-11”), 1.61 (m, 1H, *H*-11’), 0.91 (t, 3H, *J*= 7.3 Hz, *H*-12); **^{13}C-NMR (100MHz, DMSO-*d^6*): δ_C** 152.5 (*C*-1), 143.6 (*C*-4), 138.1 (q, *J*= 39.6 Hz, *C*-5), 133.2 (*C*-8), 131.4 (*C*-7), 124.5 (*C*-9), 123.1 (*C*-3), 122.1 (*C*-6), 118.7 (*C*-2), 118.7 (q, *J*= 271.43 Hz, *C*-15), 62.5 (*C*-10), 54.8 (*C*-13), 50.9 (*C*-11), 45.4 (*C*-14), 25.0 (*C*-11), 10.6 (*C*-12); **LR-ESI-MS:** $C_{16}H_{20}F_3N_6$ $[M+H]^+$ *m/z* found 353.2, cald 353.2; **HR-ESI-MS:** $C_{16}H_{20}F_3N_6$ $[M+H]^+$ *m/z* found 353.1731, cald 353.1702.

N_2,N_2-dimethyl-N_1-(3-(trifluoromethyl)-[1,2,4]triazolo[3,4-a]phthalazin-6-yl)butane-1,2-diamine (89)

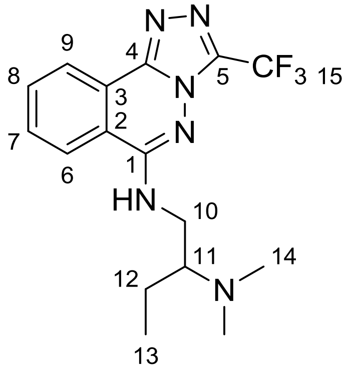

6 (300 mg, 1.1 mmol, 1 eq) and N_2,N_2-dimethylbutane-1,2-diamine (192 mg, 1.651 mmol, 1.5 eq) were reacted according to general procedure **A**. The crude material was purified by Isolera Biotage LPLC (DCM/MeOH 90:10 then DCM/MeOH/NH_3 9:1:0.5) to give **89** (247 mg, 0.702 mmol, 64%) as a white solid.

Mpt: 130.2-132.2 °C; **ν_{max} (cm^{-1})** 3263, 2930, 1565, 1501, 1454, 1178, 1074, 980, 770, 719; **^{19}F-NMR (376 MHz, DMSO-*d^6*): δ_F** -63.20; **^{1}H-NMR (400MHz, DMSO-*d^6*): δ_H** 8.48 (d, 1H, *J*= 7.6 Hz, *H*-9), 8.36 (d, 1H, *J*= 7.9 Hz, *H*-6), 8.03 (m, 1H, *H*-8), 7.89 (m, 2H, N*H*; *H*-7), 7.49 (d, 1H, *J*= 7.8 Hz, N*H*), 4.23 (m, 1H, *H*-10”), 3.32 (m, 1H, *H*-10’), 2.78 (m, 1H, *H*-11), 2.25 (s, 6H, *H*-14), 1.52 (m, 1H, *H*-12”), 1.31 (m, 2H, *H*-12’), 0.88 (t, 3H, *J*= 7.3 Hz, *H*-13); **^{13}C-NMR (100MHz DMSO-*d^6*): δ_C** 152.6 (*C*-1), 144.0 (*C*-4), 138.4 (q, *J*= 39.6 Hz, *C*-5), 133.6 (*C*-8), 132.0 (*C*-7), 124.5 (*C*-9), 123.5 (*C*-3), 122.3 (*C*-6), 119.1 (*C*-2), 118.9 (q, *J*= 269.20 Hz, *C*-15), 62.6 (*C*-10), 55.0 (*C*-11), 48.8 (*C*-14), 21.1 (*C*-12), 11.6 (*C*-13); **LR-ESI-MS:** $C_{16}H_{20}F_3N_6$ $[M+H]^+$ *m/z* found 353.3, cald 353.2; **HR-ESI-MS:** $C_{16}H_{20}F_3N_6$ $[M+H]^+$ *m/z* found 353.1722, cald 353.1702.

***N*₁,*N*₁-diethyl-*N*₂-(3-(trifluoromethyl)-[1,2,4]triazolo[3,4-a]phthalazin-6-yl)propane-1,2-diamine (90)**

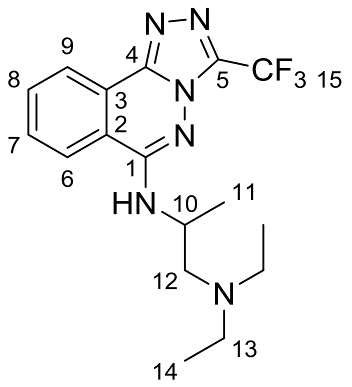

6 (300 mg, 1.1 mmol, 1 eq) and N_1,N_1-diethylpropane-1,2-diamine (215 mg, 1.651 mmol, 1.5 eq) were reacted according to general procedure **A**. The crude material was purified by Isolera Biotage LPLC (DCM/MeOH 90:10 then DCM/MeOH/NH_3 9:1:0.5) to give **90** (171 mg, 0.467 mmol, 42%) as a white solid.

Mpt: 121.1.2-123.1 °C; $\mathbf{v_{max}}$ **(cm^{-1})** 3341, 2970, 2809, 1552, 1492, 1177, 1142, 703, 467; **^{19}F-NMR (376 MHz, DMSO-*d*^{6}): δ_F** -63.16; **^{1}H-NMR (400MHz, DMSO-*d*^{6}): δ_H** 8.47 (m, 2H, *H*-9; *H*-6), 8.00 (m, 1H, *H*-8), 7.94 (m, 1H, *H*-7), 6.52 (d, 1H, *J*= 7.7 Hz, N*H*), 4.19 (m, 1H, *H*-10), 2.59 (m, 1H, *H*-12"), 2.40 (m, 1H, *H*-12'), 1.31 (d, 3H, *J*= 6.5 Hz, *H*-11), 0.93 (t, 6H, *J*= 7.1 Hz, *H*-14); **^{13}C-NMR (100MHz DMSO-*d*^{6}): δ_C** 152.0 (*C*-1), 143.7 (*C*-4), 138.1 (q, *J*= 39.61 Hz, *C*-5), 133.3 (*C*-8), 131.5 (*C*-7), 124.4 (*C*-9), 123.2 (*C*-3), 122.2 (*C*-6), 118.8 (*C*-2), 118.7 (q, *J*= 269.2 Hz, *C*-15), 57.7 (*C*-10), 47.1 (*C*-12), 45.8 (*C*-13), 18.0 (*C*-11), 12.0 (*C*-14); **LR-ESI-MS:** $C_{17}H_{22}F_3N_6$ $[M+H]^+$ *m/z* found 367.3, cald 367.2; **HR-ESI-MS:** $C_{17}H_{22}F_3N_6$ $[M+H]^+$ *m/z* found 367.1884, cald 367.1858.

***N*-((1-ethylpyrrolidin-2-yl)methyl)-3-(trifluoromethyl)-[1,2,4]triazolo[3,4-a]phthalazin-6-amine (91)**

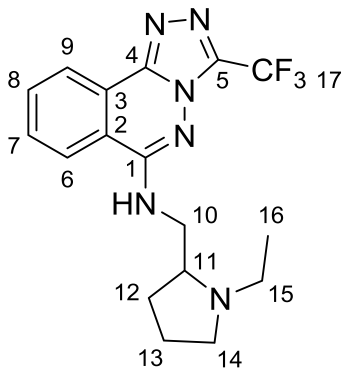

6 (300 mg, 1.1 mmol, 1 eq) and (1-ethylpyrrolidin-2-yl)methanamine (212 mg, 1.651 mmol, 1.5 eq) were reacted according to general procedure **A**. The crude material was purified by Isolera Biotage LPLC (DCM/MeOH 90:10 then DCM/MeOH/NH_3 9:1:0.5) to give **91** (336 mg, 0.929 mmol, 84%) as a white solid.

Mpt: 166.1-168.1 °C; $\mathbf{v_{max}}$ **(cm^{-1})** 3404, 2932, 2800, 1562, 1497, 1526, 1137, 976, 401; **^{19}F-NMR (376 MHz, $CDCl_3$): δ_F** -64.18; **^{1}H-NMR (400MHz, $CDCl_3$): δ_H** 8.66 (d, 1H, *J*= 7.8 Hz, *H*-9), 7.90 (m, 1H, *H*-8), 7.83 (m, 2H, *H*-6; *H*-7), 6.62 (br-s, 1H, N*H*), 3.69 (ddd, 1H, *J*= 13.6, 6.8, 2.5 Hz, *H*-10"), 3.47 (m, 1H, *H*-10'), 3.34 (dt, 1H, *J*= 9.2, 4.7 Hz, *H*-11), 2.89 (m, 2H, *H*-14), 2.35 (m, 2H, *H*-15), 2.03 (m, 1H, *H*-12"), 1.79 (m, 3H, *H*-12'; *H*-13), 1.16 (t, 3H, *J*= 7.2 Hz, *H*-16); **^{13}C-NMR (100MHz $CDCl_3$): δ_C** 152.4 (*C*-1), 144.0 (*C*-4), 139.8 (q, *J*= 41.1 Hz, *C*-5), 132.9 (*C*-8), 131.4 (*C*-7), 124.3 (*C*-9), 123.1 (*C*-3), 122.5 (*C*-6), 119.0 (*C*-2), 118.6 (q, *J*= 268.5 Hz, *C*-15), 61.8 (*C*-11), 53.5 (*C*-14), 48.0 (*C*-15), 42.1 (*C*-10), 28.8 (*C*-12), 23.0 (*C*-13), 13.9 (*C*-16); **LR-ESI-MS:** $C_{17}H_{22}F_3N_6$ $[M+H]^+$ *m/z* found 365.3, cald 365.2; **HR-ESI-MS:** $C_{17}H_{22}F_3N_6$ $[M+H]^+$ *m/z* found 365.1773, cald 365.1702.

tert-butyl ((1*R*,2*S*)-1-hydroxy-1-phenylpropan-2-yl)carbamate (92)

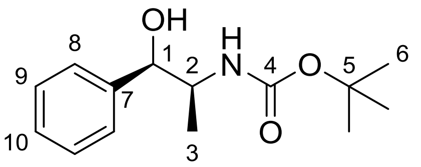

(1*R*,2*S*)-(-)-Norephedrine (4.02 g, 26.6 mmol, 1 eq) was dissolved in CH_2Cl_2 (89 mL, 0.3 M, anhydrous) under an inert atmosphere (N_2). DIPEA (6.95 mL, 39.9 mmol, 1.5 eq) was then added dropwise before the solution was cooled to 0 °C and Boc_2O (9.16 mL, 39.9 mmol, 1.5 eq) was added dropwise. The solution was allowed to stir at ambient temperature for 16 h. The reaction mixture was then concentrated to dryness and the crude material was purified by Isolera Biotage LPLC (DCM/MeOH 1:0 then DCM/MeOH 9:1) to give **92** (3.374 g, 13.42 mmol, 51%) as a white solid.

Mpt: 88.9-90.9 °C; **ν_{max} (cm^{-1})** 3358, 2984, 1679, 1523, 1448, 1160, 1013, 856, 697; **^{1}H-NMR (400MHz, $CDCl_3$):** δ_H 7.23 (m, 5H, *H*-8, *H*-9, *H*-10), 4.78 (br-s, 1H, *H*-1), 4.60 (br-s, 1H, N*H*), 3.94 (br-s, 1H, O*H*), 3.33 (br-s, 1H, *H*-2), 1.40 (s, 9H, *H*-6), 0.92 (d, 3H, *J*= 6.8 Hz, *H*-3) **^{13}C-NMR (100MHz $CDCl_3$): δ_C** 156.4 (*C*-4), 140.8 (*C*-7), 128.1 (*C*-8), 127.4 (*C*-9), 126.3 (*C*-10), 79.8 (*C*-1), 51.9 (*C*-2), 28.4 (*C*-6), 14.8 (*C*-3);

LR-ESI-MS: $C_{14}H_{22}NO_3$ $[M+H]^+$ *m/z* found 252.1, cald 252.2; **HR-ESI-MS:** $C_{14}H_{21}NNaO_3$ $[M+Na]^+$ *m/z* found 274.1432, cald 274.1419.

tert-butyl (4*S*,5*R*)-4-methyl-5-phenyl-1,2,3-oxathiazolidine-3-carboxylate 2,2-dioxide (47)

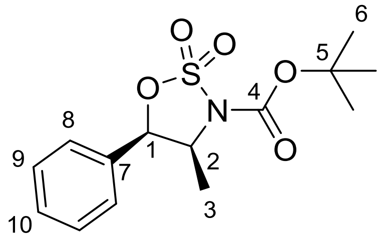

Part one:

92 (1.033g, 4.11 mmol, 1.0 eq) was dissolved in MeCN (10 mL, anhydrous) under an inert atmosphere (N_2). The solution was then added dropwise to a solution of $SOCl_2$ (373 µL, 5.14 mmol, 1.25 eq) in MeCN (10 mL, anhydrous, 0.2 M final concentration) with stirring at -40 °C. After 10 mins of stirring Pyridine (1.324 mL, 16.44 mmol, 4 eq) was then added and the mixture was stirred at -40 °C for 1 h. The mixture was then warmed to 0 °C and stirred for 1 h before H_2O (15 mL) and EtOAc (15 mL) were added. The layers were separated and the organic layers were washed with 1*N* HCl (30 mL). The combined aqueous phases were extracted with EtOAc (x3) before the organic layers were combined and washed with sat. $NaHCO_3$ (x1), brine and then dried over Na_2SO_4 before being concentrated to a brown oil.

Part two:

The residue was taken up in MeCN (20 mL) and cooled to 0 °C. The mixture then had $RuCl_3.3H_2O$ (20 mg, 0.041 mmol, 2 mol%), $NaIO_4$ (1.319 g, 6.17 mmol, 1.5 eq) and H_2O (20 mL) added sequentially before being stirred vigorously for 1 h. The mixture was then diluted with H_2O (20 mL) and Et_2O (20 mL) before the layers were separated and the organic layer was washed successively with H_2O until the brown/black colour had been completely removed. The solution was dried over Na_2SO_4 and concentrated to a residue which was triturated out of cyclohexane to give **47** (0.628 g, 2.00 mmol, 49%) as an off white solid.

Mpt: 111.3-113.3 °C; **ν_{max} (cm^{-1})** 2990, 1731, 1364, 1327, 1184, 1159, 801, 697, 629; **^{1}H-NMR (400MHz, $CDCl_3$): δ_H** 7.43 (m, 3H, *H*-8, *H*-10), 7.33 (m, 2H, *H*-9), 5.97 (d, 1H, *J*= 5.3 Hz, *H*-1), 4.58 (dq,

1H, *J*= 6.7, 5.5 Hz, *H*-2), 1.58 (s, 9H, *H*-6), 1.09 (d, 3H, *J*= 6.6 Hz, *H*-3) **^{13}C-NMR (100MHz CDCl$_3$): δ$_C$** 148.4 (*C*-4), 131.3 (*C*-7), 129.4 (*C*-8), 128.9 (*C*-9), 125.4 (*C*-10), 85.5 (*C*-1), 82.3 (*C*-5), 58.6 (*C*-2), 27.9 (*C*-6), 14.2 (*C*-3); **LR-ESI-MS:** $C_{14}H_{23}N_2O_5S$ $[M+NH_4]^+$ *m/z* found 313.1, cald 313.1; **HR-ESI-MS:** $C_{14}H_{19}NNaO_5S$ $[M+Na]^+$ *m/z* found 336.0883, cald 336.0882.

tert-butyl ((1*S*,2*S*)-1-(dimethylamino)-1-phenylpropan-2-yl)carbamate (48)

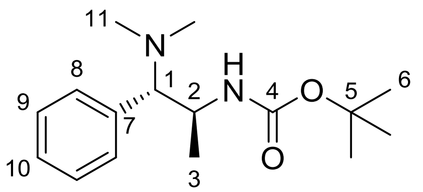

47 (0.627 g, 2.00 mmol, 1.0 eq) was dissolved in THF (2 mL, anhydrous) under an inert atmosphere (N_2) in a sealed tube. Dimethylamine (3 mL, 6.00 mmol, 3 eq, 2 M) was then added dropwise at room temperature and the mixture was allowed to stir at 80 °C for 16 h. The mixture was then concentrated down to dryness and purified by Isolera Biotage LPLC (CH/EA 9:1 then CH/EA 2:8) to give **48** (0.396 g, 1.422 mmol, 71%) as a white solid.

Mpt: 83.4-85.4 °C; **v_{max} (cm^{-1})** 3373, 2974, 1698, 1485, 1345, 1163, 1060, 863, 703; **^{1}H-NMR (400MHz, CDCl$_3$): δ$_H$** 7.33 (m, 3H, *H*-9, *H*-10), 7.19 (m, 2H, *H*-8), 4.97 (s, 1H, N*H*), 4.02 (m, 1H, *H*-2), 3.21 (d, 1H, *J*= 8.6 Hz, *H*-1), 2.14 (s, 6H, N(C*H*$_3$)$_2$), 1.48 (s, 9H, *H*-6), 1.02 (d, 3H, *H*-3); **^{13}C-NMR (100MHz CDCl$_3$): δ$_C$** 156.0 (*C*-1), 135.3 (*C*-7), 129.5 (*C*-8), 127.9 (*C*-9), 127.5 (*C*-10), 79.0 (*C*-1), 73.7 (*C*-5), 46.4 (*C*-2), 41.9 (*C*-11), 28.5 (*C*-6), 17.4 (*C*-3); **LR-ESI-MS:** $C_{16}H_{27}N_2O_2$ $[M+H]^+$ *m/z* found 279.0, cald 279.2; **HR-ESI-MS:** $C_{16}H_{27}N_2O_2$ $[M+H]^+$ *m/z* found 279.2060, cald 279.2073.

(1*S*,2*S*)-*N*$_1$,*N*$_1$-dimethyl-1-phenylpropane-1,2-diamine (93)

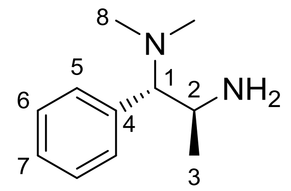

47 (0.396 g, 1.422 mmol, 1.0 eq) was dissolved in CH_2Cl_2 (3.56 mL, 0.4 M, anhydrous) under an inert atmosphere (N_2). TFA (1.09 mL, 14.22 mmol, 10 eq) was then added dropwise before the mixture was allowed to stir for 2 h. The mixture was then concentrated to dryness before being redissolved in a minimal amount of MeOH and purified by a Flash SC-X column eluting with MeOH then 7*N* in MeOH to give **93** (0.266 g, 1.492 mmol, *quant.*) as a colourless oil.

^{1}H-NMR (400MHz, CDCl$_3$): δ$_H$ 7.46 (m, 2H, *H*-7, *H*-8), 7.27 (m, 2H, *H*-6), 3.56 (m, 1H, *H*-2), 3.19 (d, 1H, *J*= 9.7 Hz, *H*-3), 2.31 (s, 6H, *H*-4), 1.03 (d, 3H, *J*= 6.2 Hz, *H*-1); **^{13}C-NMR (100MHz CDCl$_3$): δ$_C$** 130.9 (*C*-5), 129.4 (*C*-6), 128.3 (*C*-7), 71.6 (*C*-1), 46.9 (*C*-2), 40.6 (*C*-8), 16.2 (*C*-3); **LR-ESI-MS:** $C_{11}H_{19}N_2$ $[M+H]^+$ *m/z* found 179.2, cald 179.2; *Data in agreement with that found for* **32**.

(1*S*, 2*S*)-*N*$_1$,*N*$_1$-dimethyl-*N*$_2$-(3-methyl-[1,2,4]triazolo[3,4-a]phthalazin-6-yl)-1-phenylpropane-1,2-diamine (*L*-Moses)

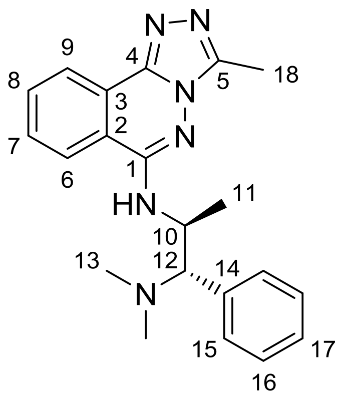

5 (35 mg, 0.160 mmol, 1 eq) and **93** (43 mg, 0.240 mmol, 1.5 eq) were reacted according to general procedure **A**. The crude material was purified by Isolera Biotage LPLC (DCM/MeOH 90:10 then DCM/MeOH/NH_3 9:1:0.5) to give ***L*-Moses** (17 mg, 0.047 mmol, 29.5%) as a white solid.

***L*-Moses** (Chiralpak®OG CH/IPA 8:2, 1 mL/min, R_t= 14.60 min, >99% *ee*)

^{1}H-NMR (400 MHz, DMSO-*d*^{6}): δ$_H$ 8.40 (d, 1H, *J*= 7.8 Hz, *H*-9), 8.28 (d, 1H, *J*= 8.3 Hz, *H*-6), 7.93 (m, 1H, *H*-8), 7.82 (m, 1H, *H*-7), 7.37 (m, 3H, *H*-15; *H*-17), 7.22 (d, 2H, *J*= 7.2 Hz, *H*-16), 7.07 (d, 1H, *J*= 5.7 Hz, N*H*), 4.70 (m, 1H, *H*-10), 3.71 (d, 1H, *J*= 8.6 Hz, *H*-12), 2.61 (s, 3H, *H*-18), 2.11 (s, 6H, *H*-13), 1.11 (d, 3H, *J*= 6.4 Hz, *H*-11); **^{13}C-NMR (100 MHz, DMSO-*d*^{6}): δ$_C$** 150.7 (*C*-1), 146.1 (*C*-4), 141.1 (*C*-5), 135.7 (*C*-14), 132.8 (*C*-8), 130.3 (*C*-7), 129.4 (*C*-15), 127.8 (*C*-16), 127.3 (*C*-17), 124.1 (*C*-9), 123.4 (*C*-3), 122.6 (*C*-6), 118.4 (*C*-2), 71.7 (*C*-12), 46.6 (*C*-10), 41.8 (*C*-13), 16.5 (*C*-11), 9.4 (*C*-18); **LR-ESI-MS:** $C_{21}H_{25}N_6$ [M+H]$^+$ *m/z* found 361.3, cald 361.2; *Data in agreement with that found for* **74**.

6-chloro-3-methyl-[1,2,4]triazolo[3,4-a]phthalazine (94)

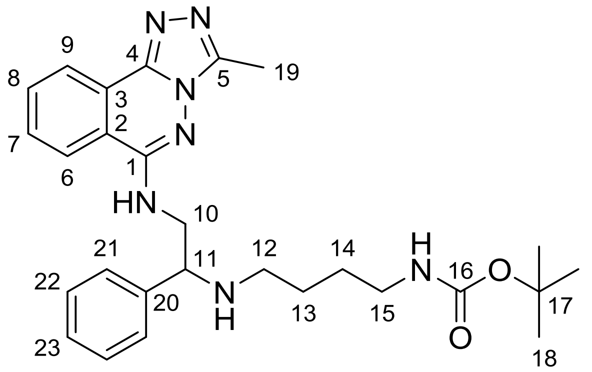

81 (163 mg, 0.514 mmol, 1 eq) was solubilised in $CHCl_3$/MeOH (5.14 mL, 2:1) under a N_2 atmosphere. tert-butyl (4-aminobutyl)carbamate (484 mg, 491 µL, 2.57 mmol, 5 eq) was then added to the solution followed by 8 drops of AcOH. NaB(CN)H_3 (38.7 mg, 0.616 mmol, 1.2 eq) was then added and the solution was warmed to 65 °C and allowed to stir for 24 h. Following completion the reaction mixture was concentrated to dryness and the crude material was purified by Isolera Biotage LPLC (DCM/MeOH 90:10) to give **94** (25 mg, 0.051 mmol, 9.9%) as a white powder.

Mpt: 212.3-214.3 °C; **v_{max} (cm^{-1})** 3309, 3217, 3067, 2927, 1678, 1515, 1250, 669; **^{1}H-NMR (400MHz, DMSO-*d*^{6}): δ$_H$** 8.37 (d, *J* = 8.7 Hz, 1H, *H*-9), 8.27 (d, *J* = 8.2 Hz, 1H, *H*-6), 7.91 (m, 1H, *H*-8), 7.81 (m, 1H, *H*-7), 7.74 (s, 1H, N*H*Boc), 7.74 (d, *J*= 7.3 Hz, 2H, *H*-21), 7.33 (t, *J*= 7.5 Hz, 2H, *H*-22), 7.23 (m, 1H, *H*-23), 6.73 (s, 1H, C-11-N*H*-C-12), 4.15 (s, 1H, *H*-11), 3.62 (s, 1H, H-10"), 3.46 (s, 1H, *H*-10'), 2.84 (m, 2H, *H*-15), 2.59 (s, 3H, *H*-19), 2.36 (s, 2H, *H*-12), 1.35 (m, 4H, *H*-13/*H*-14), 1.33 (s, 9H); **^{13}C-NMR (100 MHz DMSO-*d*^{6}): δ$_C$** 155.5, 151.1, 146.0, 141.1, 132.8, 130.1, 128.2, 127.1, 124.1, 123.3, 122.5, 118.2, 77.2, 60.6, 48.5, 47.0, 28.2, 27.4, 26.9, 9.3; **LR-ESI-MS:** $C_{27}H_{36}N_7O_2$ [M+H]$^+$ *m/z* found 490.6, cald 490.3; **HR-ESI-MS:** $C_{27}H_{36}N_7O_2$ [M+H]$^+$ *m/z* found 490.2962, cald 490.2930.

N_1-(2-((3-methyl-[1,2,4]triazolo[3,4-a]phthalazin-6-yl)amino)-1-phenylethyl)butane-1,4-diamine, 2TFA (95)

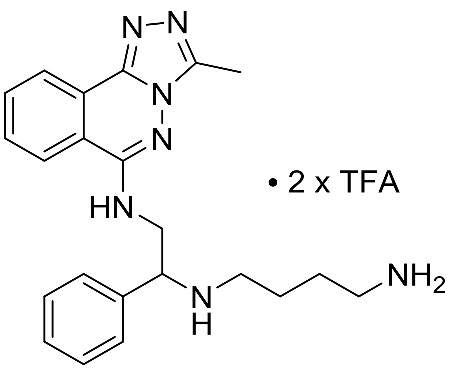

To a stirred solution of **94** (55 mg, 0.112 mmol, 1 eq) in DCM (anhydrous) (1.1 mL, 0.1 M) at room temperature was added TFA (215 µL, 2.81 mmol, 25 eq) and allowed to stir for 16 h. Upon reaction completion the reaction mixture was concentrated down to dryness to afford **95** 2xTFA salt which was submitted to the next step without further purification (66 mg, 0.108 mmol, 96%).

***N*-(4-((2-((3-methyl-[1,2,4]triazolo[3,4-a]phthalazin-6-yl)amino)-1-phenylethyl)amino)butyl)-1-(5-(2-oxohexahydro-1H-thieno[3,4-d]imidazol-4-yl)pentanamido)-3,6,9,12-tetraoxapentadecan-15-amide (46)**

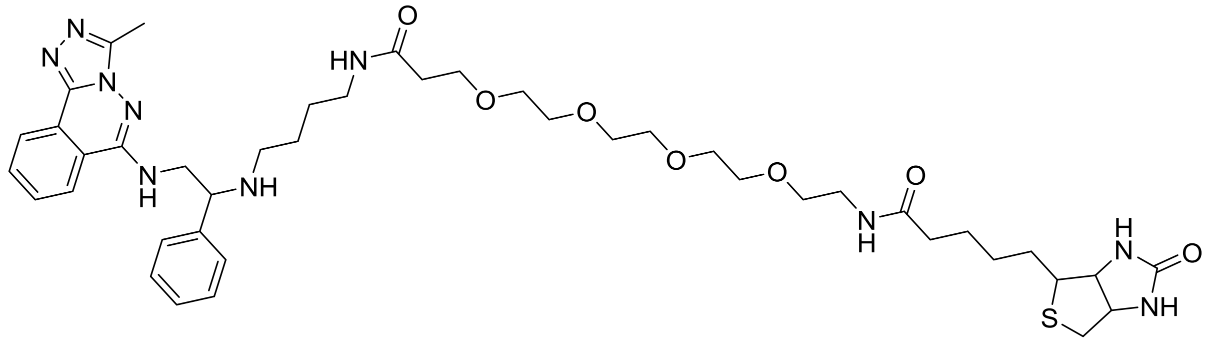

To a stirred solution of **95** (2.5 mg, 6.42 µmol, 1 eq) in DCM (642 µL, 0.01 M, anhydrous) was added 2,5-dioxopyrrolidin-1-yl 17-oxo-21-(2-oxohexahydro-1H-thieno[3,4-d]imidazol-4-yl)-4,7,10,13-tetraoxa-16-azahenicosanoate (3.8 mg, 6.42 µmol, 1 eq). The reaction mixture was stirred for 16 h after which TLC/LCMS analysis confirmed reaction completion. The reaction mixture was concentrated down to dryness and puirified via HPLC (pH 8, MeCN:H_2O) to afford **46** (2.3 mg, 2.66 µmol, 41.5%) as a white solid.

LR-ESI-MS: $C_{43}H_{63}N_{10}O_7S$ $[M+H]^+$ *m/z* found 864.1, cald 863.4. **ELSD-LCMS** 96% purity.

Protein Expression and Purification

Human bromodomains were cloned, expressed and purified as previously described.[1]

Differential Scanning Fluorimetry (DSF)

Thermal melting points were recorded using an Mx3005p Real Time PCR Machine (Stratagene). Subject proteins were buffered in 10 mM HEPES at pH 7.5, 500 mM NaCl and assayed in a 96-well plate at a final concentration of 2 µM in 20 µL volume. Compounds were then added to a final concentration of 10 µM. SPYRO Orange (Molecular Probes) was used as a fluorescent probe at a dilution of 1:1000. Excitation and emission filters for the SPYRO-Orange dye were set to 465 nm and 590 nm, respectively. The temperature was raised with a step of 3 °C/min from 25 °C to 96 °C and fluorescence readings were taken at each interval. Data was analysed as previously described.[2]

Bromodomain affinity selectivity panel of (*S*)-11

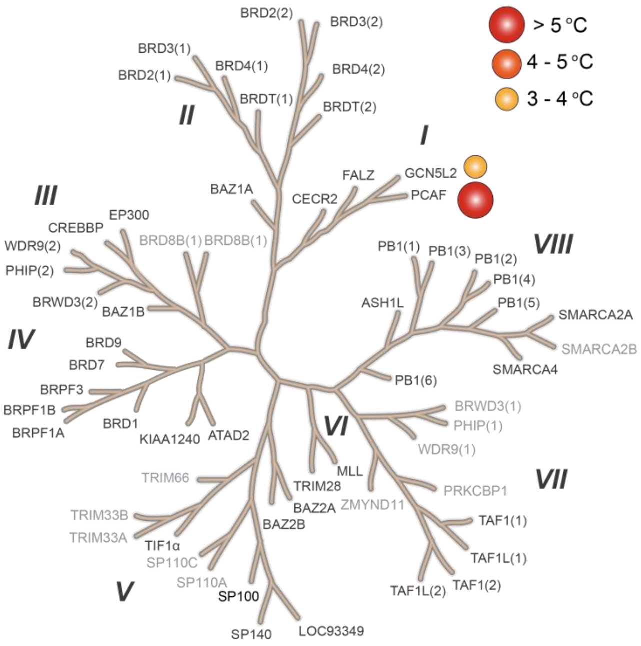

Supplemental Figure 1. DSF Selectivity panel of (*S*)-**11** against 48 human Bromodomains.

Supplemental Table 1.

Compound	PCAF ΔT_m(°C)†	GCN5L2 ΔT_m(°C)†
(*S*)-**11**	+5.4 ±0.1	+3.7 ±0.2

†Mean ΔT_m ± SEM (number of measurements= 2). Compound concentration: 10 µM

Supplemental Table 2. DSF results of selected compounds against PCAF and GCN5

Compound (10 μM)	PCAF ΔT_m (°C)	GCN5L2 ΔT_m (°C)
**9**	0.9	0.3
**11**	5.6	2.5
(S)-**11**	7.4	4.0
**12**	3.3	1.1
**13**	0.9	1.1
**14**	0	0
**15**	4.6	1.0
(S)-**17**	0.7	0.2
**50**	1.1	0.1
**51**	1.0	0
**56**	0.6	0.1
**57**	0.4	0.1
**58**	2.7	0.4
**59**	3.7	0.7
**60**	2.9	1.1
**61**	0.5	0
**62**	0.2	0.3
**63**	0.2	0
**64**	1.1	0.5
**66**	0.6	0.1
**67**	0.7	0.3
**68**	0.8	0.1
**69**	0.9	0
**70**	0	0.1
**73**	0.5	0.2
**75**	0.4	0.2
**76**	7.3	2.4
**77**	0.3	0.2
**39**	8.3	4.4
**40**	9.6	6.3
**41**	7.8	4.3
**42**	6.2	2.7
**43**	9.1	7.2
**44**	10.3	6.8
**45**	7.9	5.0
**81**	1.0	0.3
**83**	0.8	0
**84**	0.7	0.2
**85**	3.7	0.9
**86**	0.02	0.2
**88**	0.4	0.1
**89**	0.4	0.2
**90**	0.4	0.1
**91**	0.1	0.1
**94**	0	0.2
**97**	2.8	0.9
**98**	0.7	0.2
**99**	3.8	1.5

Isothermal Titration Calorimetry (ITC)

Experiments were carried out on a Nano-ITC Standard Volume Instrument (TA Instruments). All experiments were carried out at 25 °C in 20 mM HEPES pH 7.5, 150 mM NaCl, 0.5 mM TCEP and 5% glycerol. Protein solutions were buffer exchanged by gel filtration. The titrations were conducted using an initial injection of 2 µL followed by 32 injections of 8 µL. Background dilution heat was substracted from each experiment. Thermodynamic parameters were calculated using $\Delta G = \Delta H - T\Delta S = -RT\ln K_B$, where $K_D = 1/K_B$. ΔG, ΔH and ΔS are changes in free energy, enathalpy and entropy resepectively. Independent single site binding models were employed in data analysis.

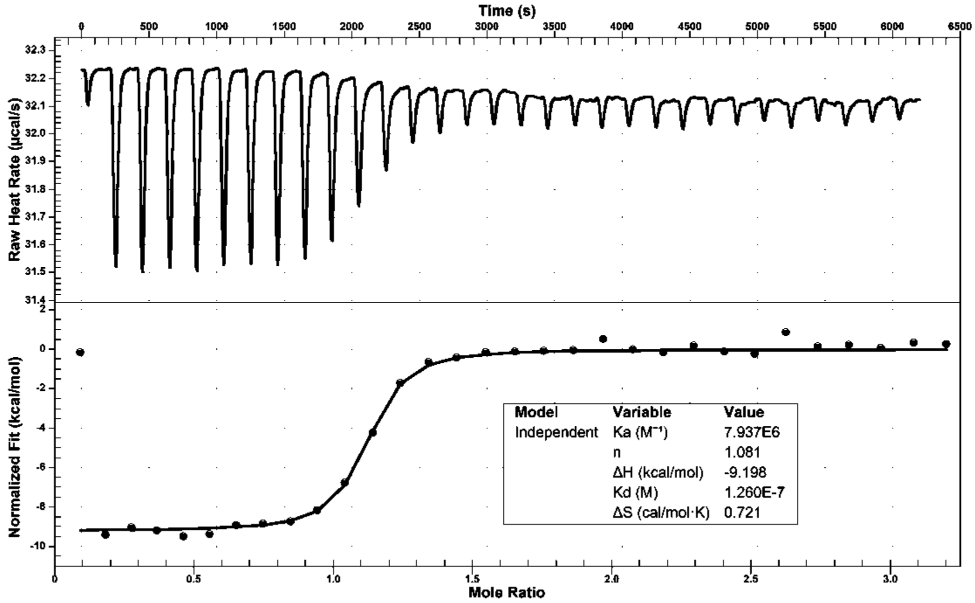

Supplemental Figure 2. ITC trace of ***L*-Moses** and PCAF Brd

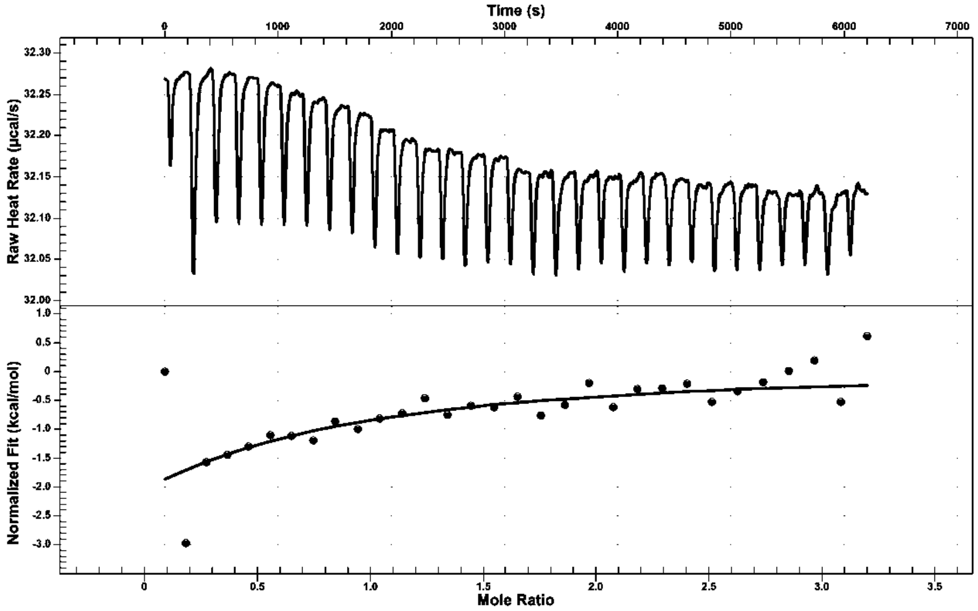

Supplemental Figure 3. ITC trace of ***D*-Moses** and PCAF Brd

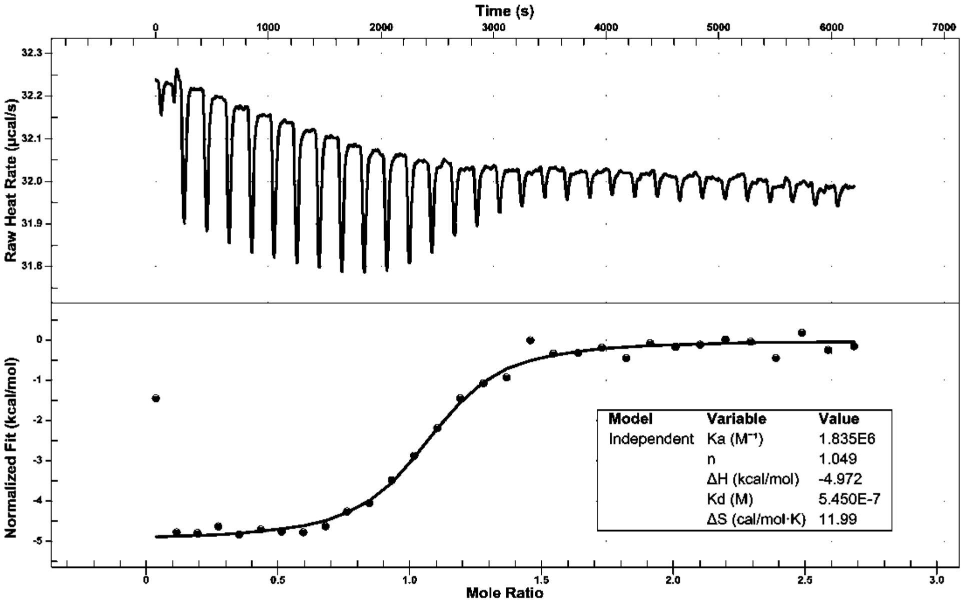

Supplemental Figure 4. ITC trace of ***L*-Moses** and *H*GCN5 Brd

ITC *Pf*GCN5

The binding of compound to the bromodomain of *Pf*GCN5 was assessed using isothermal titration calorimetry. The assay was performed at at 25 °C using a VP-ITC instrument (MicroCal, Northanpton, MA, USA) with 0.2 mM recombinant *Pf*GCN5 and 0.01 mM ***L*-Moses**, both in a buffer of 20 mM HEPES, 100 mM NaCl , pH 7.5. The experimental data were fitted to a theoeretical titration curve using the software package Origin (MicroCal), resulting in a calculated K_D of (2.8 ± 0.4) x 10^{-7} M.

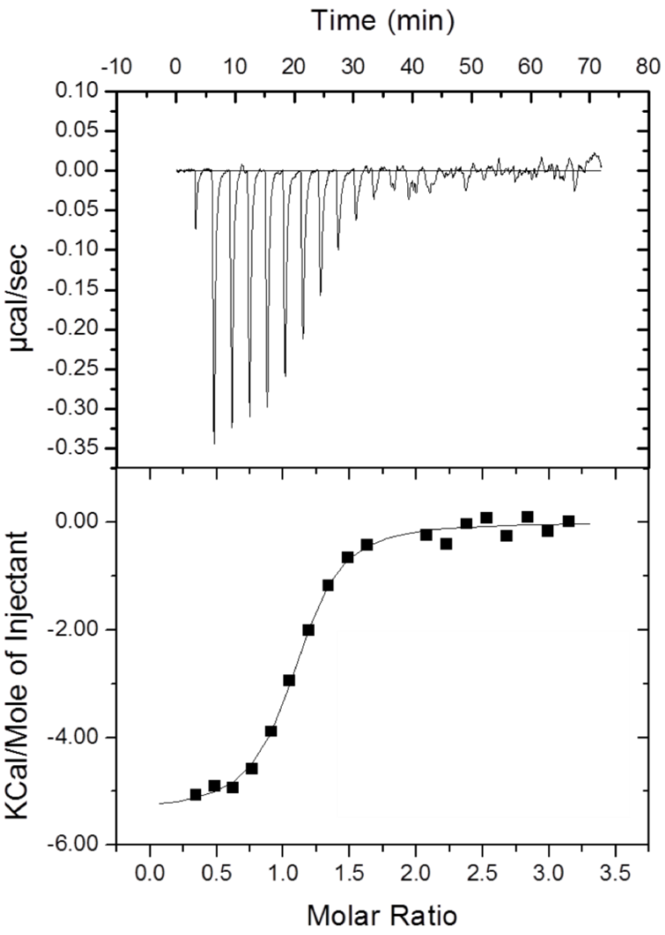

Supplemental Figure 5. ITC trace of ***L*-Moses** and *Pf*GCN5 Brd

Supplemental Table 3. Binding affinity of selected compounds (ITC).

Compound	PCAF K_D (µM)┼	
**7**	7.98	±0.65
**8**	>30	-
**9**	>30	-
**11**	0.298	±0.039
**(*S*)-11**	0.284	±0.029
**12**	1.76	±0.23
**13**	>30	-
**14**	>30	-
**15**	7.30	±1.1
**16**	6.90	±1.4
**(*S*)-17**	>30	-
**56**	>30	-
**57**	>30	-
**59**	4.30	±0.31
**60**	1.13	±0.24
**62**	>30	-
**64**	>30	-
**65**	>30	-
**66**	>30	-
**67**	0.321	±0.039
**68**	>30	-
**69**	>30	-
**70**	>30	-
**71**	2.3	±0.93
**72**	>30	-
**73**	>30	-
**75**	>30	-
**76**	0.168	±0.023
**39**	0.195	±0.040
**40**	0.133	±0.015
**41**	0.160	±0.054
**42**	0.223	±0.078
**43**	0.163	±0.117
**44**	0.179	±0.048
**45**	0.168	±0.027
**83**	>30	-
**84**	>30	-
**85**	>30	-
**86**	>30	-
**87**	>30	-
**88**	>30	-
**89**	>30	-
**90**	>30	-
**91**	>30	-
**97**	18.2	±0.13
***L*-45/*L*-Moses**	**0.126**	**±0.015**
***L*-45/*D*-Moses**	>30	-

┼ Compound concentration: 30 µM, Protein concentration: 280-320 µM.

NanoLuciferase Bioluminescent Resonance Energy Transfer (NanoBRET) Assay

Methods

HEK293 cell (8 x 10^5) were plated in each well of a 6-well plate and co-transfected with Histone H3.3-HaloTag (NM_002107) and a NanoLuciferase fusion of the isolated bromodomain of PCAF. Twenty hours post-transfection, cells were collected, washed with PBS, and exchanged into media containing phenol red-free DMEM and 4% FBS in the absence (control sample) or the presence (experimental sample) of 100 nM NanoBRET 618 fluorescent ligand (Promega). Cell density was adjusted to 2 x 10^5 cells/ml and then re-plated in a 96-well assay white plate (Corning Costar #3917). Compounds were then added directly to media at final concentrations 5 µM or an equivalent amount of DMSO as a vehicle control, and the plates were incubated for 18 h at 37 ^{o}C in the presence of 5% CO_2. NanoBRET Nano-Glo substrate (Promega) was added to both control and experimental samples at a final concentration of 10 µM. Readings were performed within 5 minutes using a Glo-MAX Discover instrument (Promega) equipped with 450/8 nm bandpass and 600 nm longpass filters with a 0.3 sec reading setting. A corrected BRET ratio was calculated and is defined as the ratio of the emission at 600 nm/450 nm for experimental samples (i.e. those treated with NanoBRET fluorescent ligand) subtracted by and the emission at 600 nm/450 nm for control samples (not treated with NanoBRET fluorescent ligand). BRET ratios are expressed as milliBRET units (mBU), where 1 mBU corresponds to the corrected BRET ratio multiplied by 1000.

***In vitro* Metabolism Studies**

Metabolic stability studies were carried out exposing nominated compounds to samples of liver microsomes. Compounds were analysed by LC-MS for loss of compound at 0, 5, 15, 30 and 45 minute time points. The rate of metabolic degradation of compounds was used to calculate CL_{int}, SE CL_{int}, $t_{1/2}$ (summarised in supplemental table 2.)

Supplemental Table 4. *In vitro* metabolic stability of compounds ***DL*-45/*DL*-Moses** and **39**

Compound	Metbolic Stability (Human)				Metbolic Stability (Mouse)			
	CL_{int} (µL/min/mg protein)	SE CL_{int}	$t_{1/2}$	n	CL_{int} (µL/min/mg protein)	SE CL_{int}	$t_{1/2}$	n
**45/*DL*-Moses**	35	2.2	40	5	37	2.6	38	5
**39**	29	2.0	48	5	21	1.3	65	5

Cytotoxicity Studies

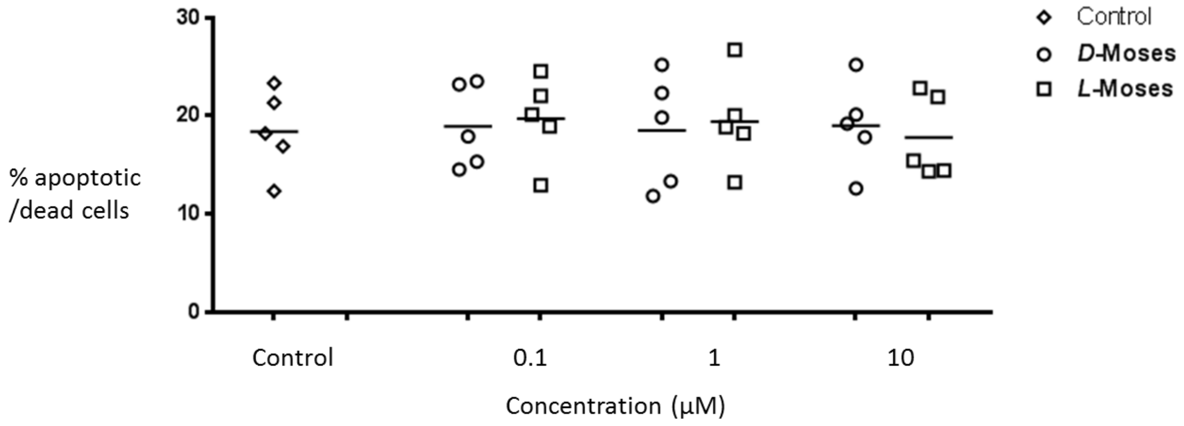

Supplemental Figure 6. Cytotoxicity studies on PMBC cells of ***L*-Moses** and ***D*-Moses**

Toxicity of ***D*-Moses** and ***L*-Moses** were tested on peripheral blood mononuclear cells (PBMC) obtained from 5 healthy donors. PBMC were cultured either with ***D*-Moses** or ***L*-Moses** at concentrations of 0.1, 1 and 10 µM or with a control (DMSO) for 24 hours. Viability of PBMC were then checked using LIVE/DEAD Fixable Aqua Dead Cell Stain Kit (ThermoFisher Scientific).

High Throughput Time Resolved Fluorescence Assay (HTRF)

HTRF assay was carried out using a Cisbio Epigenous kit B (62BDBPEH) using the standard assay protocol with GST-PCAF$_{BRD}$.[3] Final assay concentrations: GST-PCAF$_{BRD}$ (20 nM), SA-XL665 (62.5 nM), α-GST donor (62.5 nM), compound **46** (500 nM). K_i was calculated using the Cheng-Prussof equation with a value of 400 nM for the K_D of compound **46**.

Crystallography

Recombinant *Pf*GCN5 (bromodomain only) was expressed in *E. coli* and purified using a previously described system [4]. The protein crystallized with the compound ***L*-45** at 293 K in 0.1M $CaCl_2$, 30% PEG 8k, 0.2 M NH_4SO_4. The concentrations of the protein sample and the compound were 10 mg/ml and 2 mM, respectively.

X-ray diffraction data for the crystal was collected at 100 K at the beamline 08ID-1 of the Canadian Macromolecular Crystallography Facility. The dataset was processed using the HKL-2000 suite [5], and the structure solved using Phaser [6], a component of the CCP4 suite [7] with molecular replacement model 4QNS. Iterative model building using the graphics program COOT [8], the refinement program Buster-TNT [9] and the validation tools of MOLPROBITY [10] led to a model with a working R value of 0.185 and a R-free value of 0.236 over a resolution range between 37.5-2.10 Å, with excellent stereochemistry.

Supplemental Table 4. Crystallography data and refinement statistics

**PDB Code**		5TPX
**Data collection**		
Space group		$P42_12$
Cell dimensions		
a, *b*, *c* (Å)		75.00 75.00 49.62
α, β, γ (°)		90.0, 90.0, 90.0
Resolution (Å) (highest resolution shell)		37.50-2.10 (2.14-2.10)
Measured reflections		65686
Unique reflections		8411
R_{meas}(%)		10.0(72.5)
$I/\sigma I$		20.6 (2.2)
Completeness(%)		96.0(89.0)
Redundancy		7.8(7.8)
**Refinement**		
Resolution (Å)		37.5-2.1
No. reflections (test set)		8368(390)
R_{work}/ R_{free} (%)		18.5/23.6
No. atoms		
Protein		870
Water		66
Heterogen		43
**Average B-factors (Å²)**		
Protein	45.06	
Water	49.12	
Heterogen	54.5	
**Geometry (RMSD)**		
Bond lengths (Å)		0.010
Bond angles (º)		0.67
**Ramachandran plot % residues**		
Favored		100.0
Additional allowed		0.0
Disallowed		0

Chiral HPLC Traces

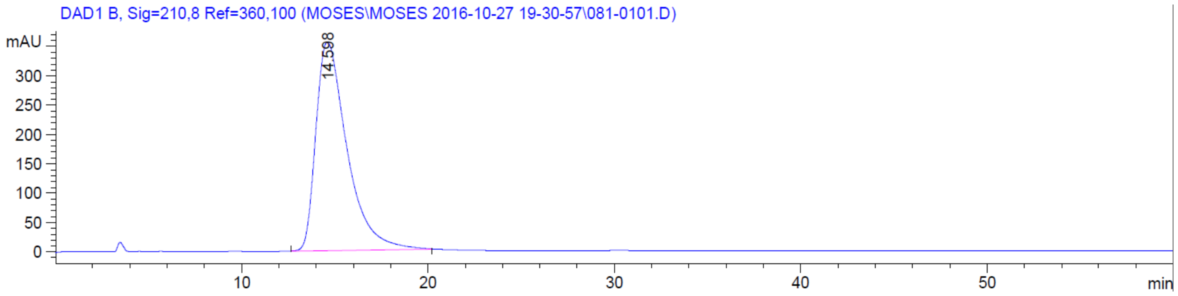

Supplemental Figure 7. HPLC trace of ***L*-45**/***L*-Moses** after asymmetric synthesis from (1*R*,2*S*)-(-)-Norephedrine. (Chiralpak®OG CH/IPA 8:2, 1 mL/min, R_t= 14.59 min, >99% *ee*)

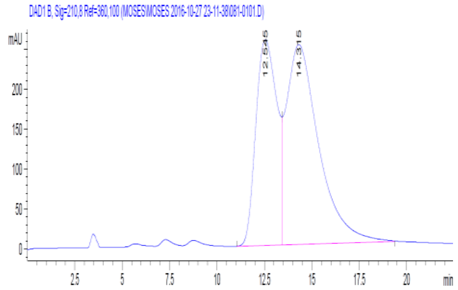

Supplemental Figure 8. HPLC trace of **45/*DL*-Moses**. Chiralpak®OG CH/IPA 8:2, 1 mL/min, R_t= 12.54 min and 14.32 min, 0% *ee*)

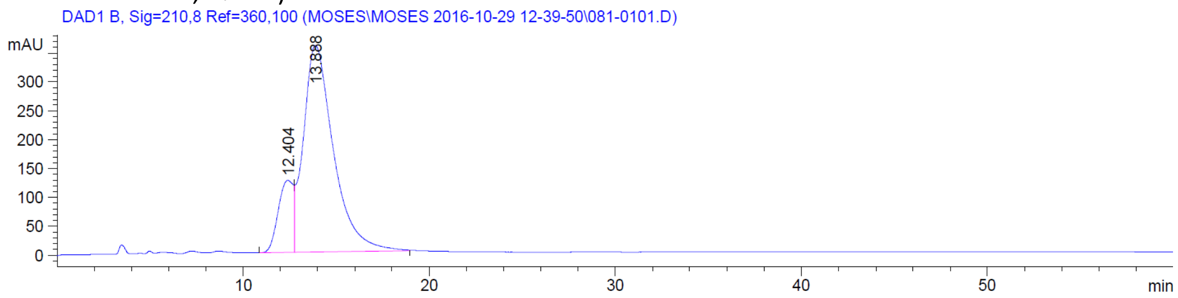

Supplemental Figure 9. HPLC trace of ***L*-45**/***L*-Moses** spiked with ~15% **45**/***DL*-Moses**. Chiralpak®OG CH/IPA 8:2, 1 mL/min, R_t= 12.40 min and 13.88 min, 70% *ee*)

Supplemental References

[1] P. Filippakopoulos, S. Picaud, M. Mangos, *et al.*, *Cell* **2012**, *149*, 214-231.

[2] P. Filippakopoulos, J. Qi, S. Picaud, *et al.*, *Nature* **2010**, *468*, 1067-1073.

[3] http://www.cisbio.com/drug-discovery/epigeneous-binding-domain-assays.

[4] M. Vedadi, J. Lew, J. Artz, *et al.*, *Mol. Biochem. Parasitol.* **2007**, *151*, 100-110.

[5] Z. Otwinowski, W. Minor, et al., *Methods Enzymol.* **1997**, *276*, 307-326.

[6] A. J. McCoy, R. W. Grosse-Kunstleve, P. D. Adams, *et al.*, *J. Appl. Crystallogr.* **2007**, *40*, 658-674.

[7] M. D. Winn, C. C. Ballard, K. D. Cowtan, *et al.*, *Acta crystallogr. D, Biol. Crystallogr.* **2011**, *67*, 235-242.

[8] P. Emsley, K. Cowtan, *Acta crystallogr. D, Biol. Crystallogr.* **2004**, *60*, 2126-2132.

[9] E. Blanc, P. Roversi, C. Vonrhein, *et al.*, *Acta crystallogr. D, Biol. Crystallogr.* **2004**, *60*, 2210-2221.

[10] V. B. Chen, W. B. Arendall, 3rd, J. J. Headd, *et al.*, *Acta crystallogr. D, Biol. Crystallogr.* **2010**, *66*, 12-21.
